# Co-catalyzed *ortho*-C–H functionalization/annulation of arenes and alkenes with alkynylsilanes: access to isoquinolone and pyridone motifs†

**DOI:** 10.1039/c9ra06963a

**Published:** 2019-09-26

**Authors:** Cong Lin, Liang Shen

**Affiliations:** College of Chemistry and Chemical Engineering, Jiangxi Science & Technology Normal University Nanchang 330013 China conglin0127@jxstnu.com.cn; Jiangxi Engineering Laboratory of Waterborne Coatings, College of Chemistry and Chemical Engineering, Jiangxi Science & Technology Normal University Nanchang 330013 China

## Abstract

A method for cobalt-catalyzed *ortho*-C–H functionalization annulation of arenes and alkenes with alkynylsilanes assisted by 8-aminoquinolyl auxiliary has been described. Alkynylsilanes were employed as the coupling partners to react with a broad range of benzamides and acrylamides, affording the corresponding isoquinolone and pyridone derivatives in moderate to high yields. It is worth noting that the silyl group in the final products can be retained or removed by switching the reaction conditions.

## Abstract 2

A method for cobalt-catalyzed *ortho*-C–H functionalization annulation of arenes and alkenes with alkynylsilanes assisted by 8-aminoquinolyl auxiliary.

## Graphical abstract

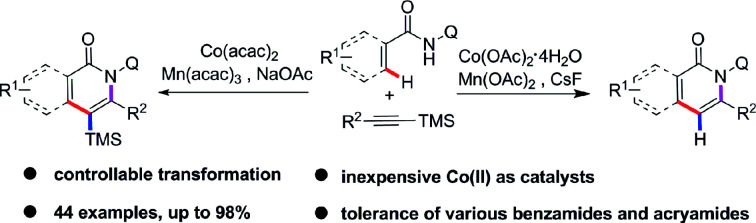

Isoquinolone represents a ubiquitous structural motif that occurs in a broad range of biologically active compounds and pharmaceuticals.^1^ Consequently, the development of efficient preparative methods for isoquinolone derivatives has attracted considerable attention from synthetic chemists. Over the past few decades, transition-metal-catalyzed direct functionalization of inert C–H bonds has aroused tremendous attention for the efficient construction of structurally diversified and synthetically useful heterocycles in a step- and atom-economical manner.^2^ Among various reaction patterns of C–H bond functionalization, the annulation of C(sp^2^)–H bonds with alkynes and alkenes has attracted considerable interest owing to the huge value of the corresponding isoquinolone products in both academic research and industry.^3^ Nevertheless, the involvement of expensive fifth and sixth row metal-based complexes, including palladium, rhodium, ruthenium and iridium, was indispensable in these transformations. Despite great achievements in the realm of C–H annulation, the challenge of realizing the direct C–H annulation of arenes and alkenes by using inexpensive metal catalyst still remains.^4^

Cobalt has been widely used as a catalyst in various C–H bond functionalization reactions due to its unique catalytic reactivity and being inexpensive with low toxicity.^5^ Significant efforts have been made to achieve Co-catalyzed C–H activation to construct C–C and C–X bonds, as pioneered by Yoshikai,^6^ Matsunaga/Kanai,^7^ Daugulis,^8^ Ackermann^9^ and others,^10^ further proving the excellent catalytic properties of cobalt catalysts. Over the past decade, strategies involving bidentate directing groups have proved to be highly effective in Pd-, Ru-, Ni-, Cu-, Fe-, and Co-catalyzed C(sp^2^)–H and C(sp^3^)–H bond functionalization reactions to realize a variety of useful transformations.^11,12^ In 2014, Daugulis first reported a Co-catalyzed, aminoquinoline and picolinamide-directed annulation of C(sp^2^)–H bond with alkynes^8*a*,*b*^ and alkenes^8*c*^ to form the corresponding isoquinolone derivatives. Later on, the cobalt(ii)-catalyzed decarboxylative C–H activation/annulation of benzamides and alkynyl carboxylic acids has been described by Song.^12*d*^ Employing the same catalytic system, Nicholls developed the regioselective C(sp^2^)–H activation of amides with 1,3-diynes by cobalt catalysis, affording alkynylated isoquinolinones.^13^ Recently, Zhai and Jeganmohan demonstrated cobalt-catalyzed annulation of C(sp^2^)–H with both internal and terminal alkynes to give an isoquinoline backbone by using 2-(1-methylhydrazinyl)pyridine and 8-aminoquinolyl as a bidentate directing group.^14^ More recently, Ilies and Nakamura achieved the oxidative C–H activation approach to pyridone and isoquinolone through an iron-catalyzed coupling of amides with alkynylsilanes.^15*c*^ Inspired by the above encouraging achievements, we are intrigued by the reaction of cobalt-catalyzed annulation of C(sp^2^)–H bonds with readily accessible alkynylsilanes.^15^ It was observed that the direct annulation of arenes and alkenes could be realized by the assistance of an 8-aminoquinolyl auxiliary to lead to the corresponding isoquinolone and pyridine motifs^16^ in moderate to high yields. Therefore, we report an efficient and straightforward strategy of cobalt-catalyzed *ortho*-C–H functionalization/alkynylsilanes annulation of arenes and alkenes assisted by a bidentate directing group (Scheme 1). To our delight, the silyl group on the final products can be retained or removed by switching the reaction conditions.

**Scheme 1:**
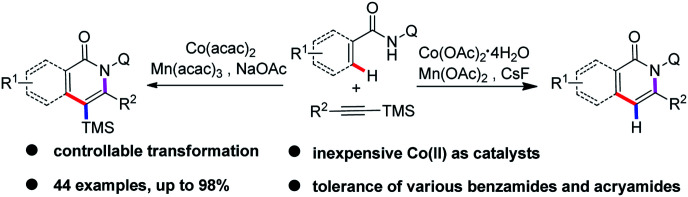
Co-catalyzed *ortho*-C–H functionalization/annulation of arenes and alkenes with alkynylsilanes.

We chose the *N*-(quinolin-8-yl)benzamide 1a and trimethyl(phenylethynyl)silane 2a as model substrates to our investigation (see the ESI†). The reaction was performed by the employment of Co(OAc)_2_ as catalyst, Mn(OAc)_2_ as oxidant, and CsF as additive in TFE at 100 °C under O_2_ atmosphere. To our delight, the reaction proceeded smoothly to afford the desired product 3a in 85% yield, and along with generating a small amount of 7a. Then, other common cobalt catalysts were screened, including CoCl_2_, CoI_2_, CoBr_2_, Co(OAc)_2_·4H_2_O, Co(acac)_2_, and Co(acac)_3_. It was found that Co(OAc)_2_·4H_2_O showed the best catalytic reactivity and gave the acyloxylated product 3a in the highest yield. Among other desilylation additives, CsF turned out to be the most essential in the formation of desired product 3a. An investigation of alternative oxidants that the combination of Mn(OAc)_2_ and O_2_ enhanced the yield of the cobalt catalytic. The control experiments indicated that the reaction did not occur in the absence of cobalt catalyst or oxidants, respectively. Further optimization towards the solvents revealed that TFE was superior to other solvents. Intriguingly, treatment of 1a (0.1 mmol) with 2a (2, 0.3 mmol) in the presence of a catalytic amount of Co(acac)_2_, Mn(acac)_3_ (2 equiv.) and NaOAc (2 equiv.) as oxidant and base respectively, at 100 °C under O_2_ atmosphere for 24 h, led to the trimethylsilyl substituted isoquinolone 7a in 76% yield.

With the optimized reaction conditions in hand, we first investigated the scope of alkynylsilanes (Table 1). Gratifyingly, the results showed that different substituted alkynylsilanes 2, including phenyl, heterocyclic, and alkyl groups, were all compatible in the present reaction (3a–3c, 3e–3f). It was worth mentioning that the 1,2-bis(trimethylsilyl)ethyne were also applicable under the current reaction conditions, affording the desired products containing trimethylsilyl (3f, 64%).^17^ However, the reason for the mono-desilylation and the regioselectivity of desilylation in 3f was not clear.

**Table 1:** Substrate scope of alkynylsilanes^a^^,^^b^

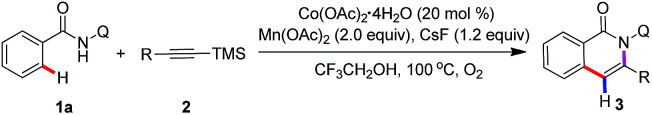
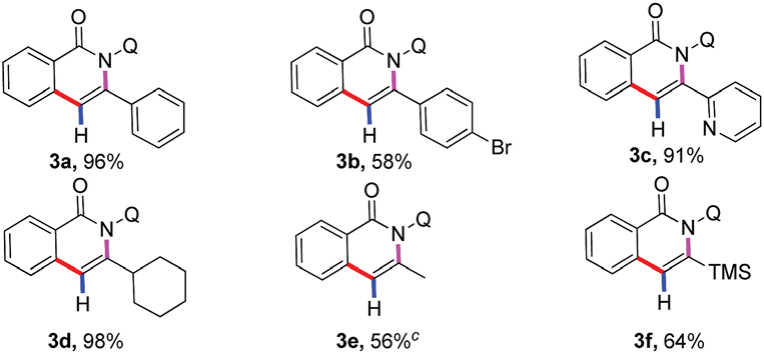

a Reactions were carried out by using 1a (0.1 mmol), 2 (0.3 mmol), Co(OAc)_2_·4H_2_O (0.02 mmol), Mn(OAc)_2_ (0.2 mmol), CsF (0.12 mmol), TFE (1.0 ml), 100 °C, O_2_, 24 h.

b Isolated yield.

c CsF (0.3 mmol), 120 °C.

After examining the compatibility of the annulation reaction with a variety of alkynylsilane substrates, our attention was turned towards the reactivity of different benzamides 1 (Table 2). The aryl ring possessing both electron-donating (4a–4f) and electron-withdrawing (4g–4l) substituents were reactive in the reaction, producing the isoquinolones in moderate to good yields. It was observed that halogenated substituents, such as iodo (4g), bromo (4h), chloro (4i–4k), fluoro (4l), were well tolerated in this transformation to give the corresponding annulated products in moderate to high yields, which provided the opportunity of further derivatization of the obtained products. More importantly, several fused-cyclic and heterocyclic moieties, such as naphthyl, thiophenyl, and furyl group, could serve as viable substrates in the reaction for the successful construction of the corresponding annulated products (4m–4p).

**Table 2:** Substrate scope of benzamides^a^^,^^b^

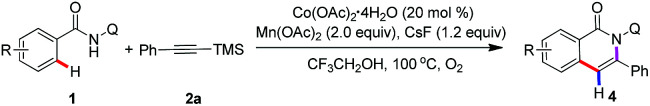
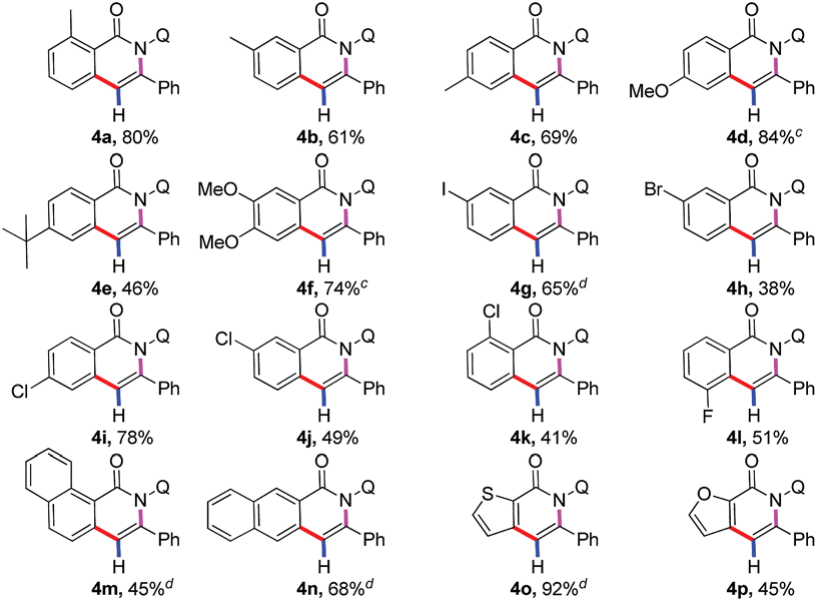

a Reactions were carried out by using 1a (0.1 mmol), 2 (0.3 mmol), Co(OAc)_2_·4H_2_O (0.02 mmol), Mn(OAc)_2_ (0.2 mmol), CsF (0.12 mmol), TFE (1.0 ml), 100 °C, O_2_, 24 h.

b Isolated yield.

c CsF (0.3 mmol).

d CsF (0.3 mmol), 120 °C.

Much progress has been made in the realm of C–H activation reaction of arenes in recent years. In contrast, direct functionalization involving alkene substrates remains less to some extent. The direct C–H functionalization by simple extension of the substrates from arenes to alkenes has proven to be difficult. Gratifyingly, it was found that our cobalt catalytic system was also effective for the more challenging C(sp^2^)–H bond of acrylamide substrates as shown in Table 3. Highly regioselective direct annulation of alkenes could be achieved without modification to the protocol developed for arenes. Various α- and β-substituted acrylamides participated in the reaction smoothly to give the pyridone motifs (6a–6j), which are the privileged structural motif in terms of many complex natural products and pharmaceutical compounds with a broad range of biological activities. It is worth noting that α,β-disubstituted alkenes showed good reactivity to deliver the multiple-substituted pyridone derivatives (6a–6d).

**Table 3:** Substrate scope of acrylamides^a^^,^^b^

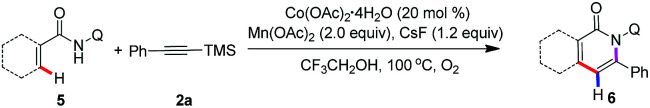
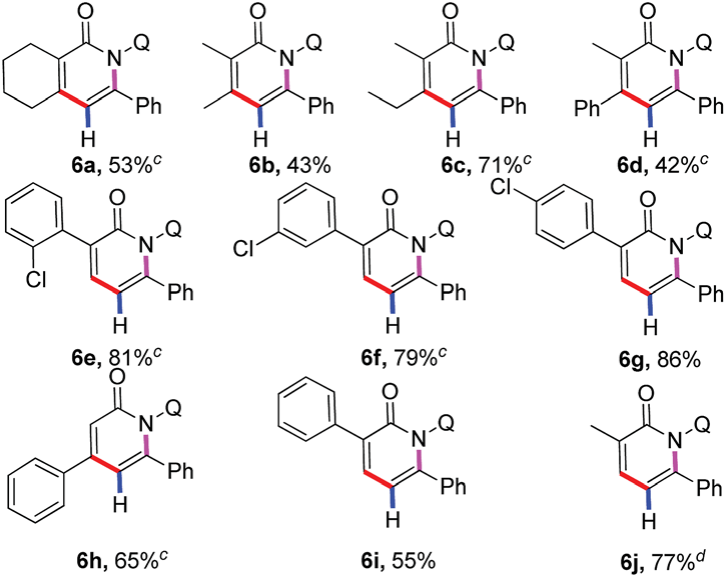

a Reactions were carried out by using 1a (0.1 mmol), 2 (0.3 mmol), Co(OAc)_2_·4H_2_O (0.02 mmol), Mn(OAc)_2_ (0.2 mmol), CsF (0.12 mmol), TFE (1.0 ml), 100 °C, O_2_, 24 h.

b Isolated yield.

c CsF (0.3 mmol).

d CsF (0.3 mmol), 120 °C.

Subsequently, the applicability of this protocol to establish trimethylsilyl substituted^18^ isoquinolones and pyridones was investigated (Table 4). Various substituted alkynylsilanes, including aryl, heterocyclic, alkyl, and conjugated alkynylsilanes, were also viable in the standard condition to give the trimethylsilyl substituted isoquinolones in moderate to good yields (7a–7f). Of note, the annulation between *N*-(quinolin-8-yl)benzamide and ((3-chlorophenyl)ethynyl)trimethylsilane or trimethyl(3-methylbut-3-en-1-yn-1-yl)silane proceed to form a mixture of regioisomeric products (7c and 7c′, 7e and 7e′) in 1 : 1 ratio as a result of the same steric bulk of the two groups substituted on the ethyne. It is worth mentioning that iodo and chloro substituted benzamides showed moderate reactivity to deliver the annulation products (7f–7g). Other amides, such as thiophene amide, participated in the reaction successfully to afford the relevant isoquinolone 7h in good yields. Importantly, the acrylamides displayed moderate reactivity and high regioselectivity to afford trimethylsilyl substituted pyridones (7i–7j), the regioselectivity between TMS and aryl group was according to the previous work, which reported by Nakamura.^15*c*^

**Table 4:** The protocol to establish trimethylsilyl substituted isoquinolones and pyridones^a^^,^^b^

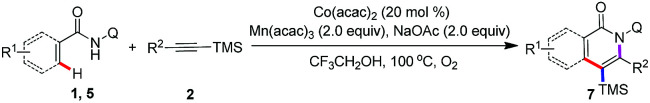
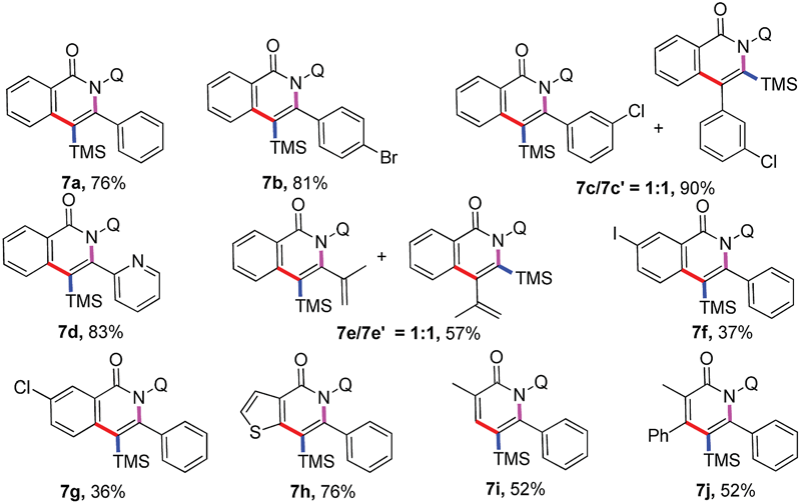

a Reactions were carried out by using 1 or 5 (0.1 mmol), 2 (0.3 mmol), Co(acac)_2_ (0.02 mmol), Mn(acac)_3_ (0.2 mmol), NaOAc (0.2 mmol), TFE (1.0 ml), 100 °C, O_2_, 24 h.

b Isolated yield.

This protocol is readily scalable, and when the reaction was scaled up to 1 mmol with a sub-gram scale, the isoquinolone motifs 3a and 7a was isolated in 93% and 71% yield, respectively (Scheme 2). This observation further demonstrated the synthetic applications of this method. Unfortunately, we attempted to transform of silyl group and remove of directing group, and it does not succeed.

**Scheme 2:**
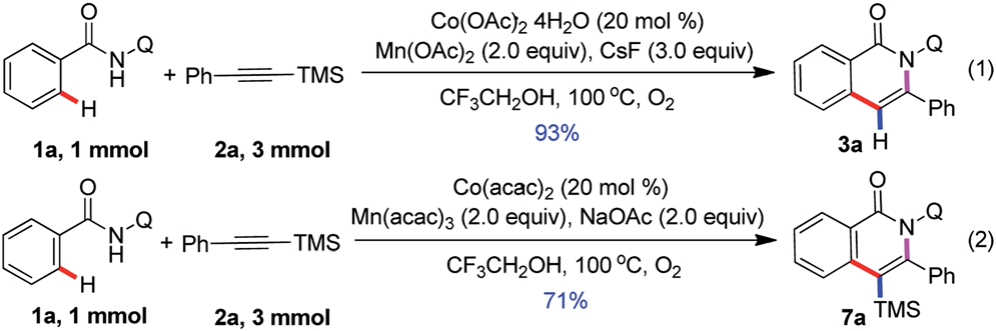
Sub-gram-scale synthesis of 3a and 7a.

To gain insight into the mechanism, we performed deuterium-labeling experiments (Scheme 3). The reaction of substrate 1a with the D_2_O in the absence of trimethyl(phenylethynyl)silane under the standard reaction conditions was performed and no deuterium–proton exchange was observed (eqn (3)). The result illustrated that the C–H cleavage step was irreversible. In addition, the parallel intermolecular kinetic isotopic experiments were conducted and the KIE value was determined to be 1.3 and 1.2, respectively, (eqn (4) and (5)) thus implying that C–H cleavage was not involved in the rate-determining step.

**Scheme 3:**
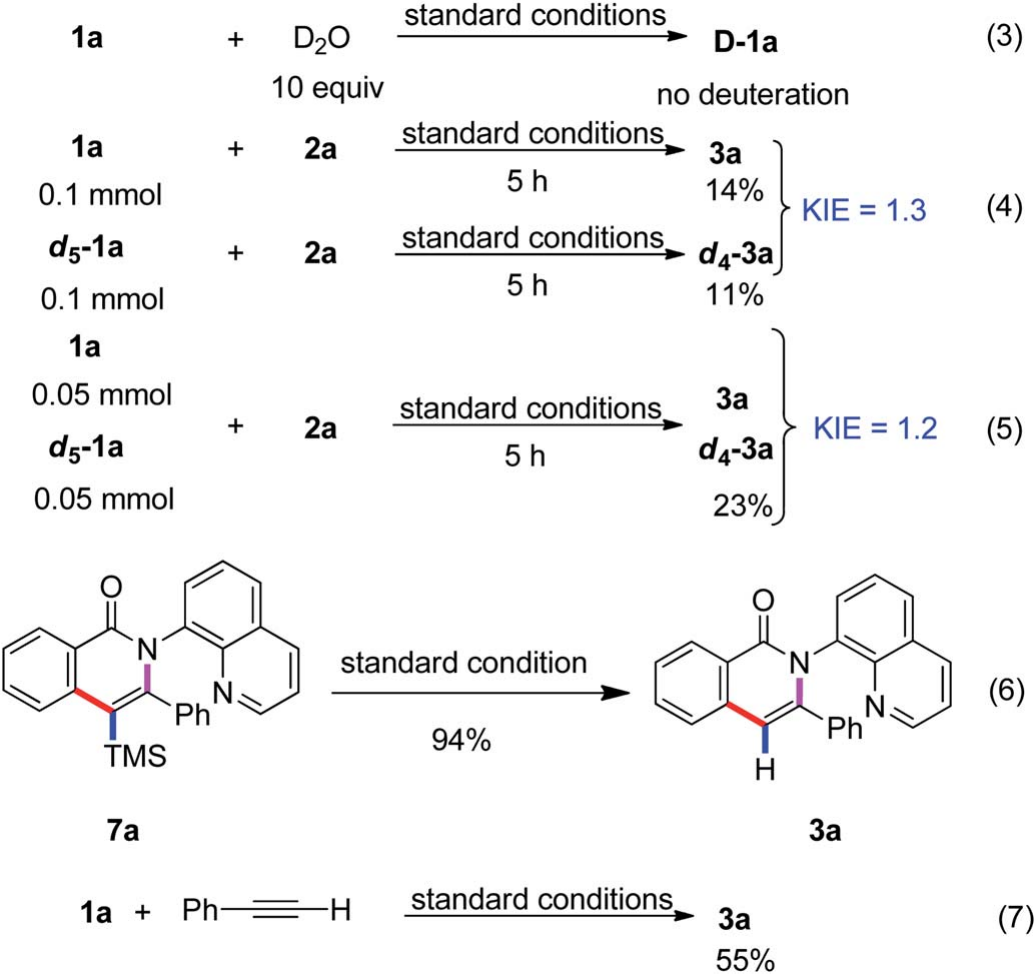
Deuterium labelling experiments.

Based on the above experimental results and previous reports,^5–14^ a plausible reaction pathway is proposed as depicted in Scheme 4. First, the oxidation of Co(ii) by Mn^II^ or Mn^III^ and O_2_ to give Co(iii) species, which coordinates with the nitrogen atom in 8-aminoquinolyl auxiliary followed by a ligand exchange to form the intermediate A. The cleavage of C(sp^2^)–H bond of intermediate A binds to the Co(iii) center to lead to the cyclometalated intermediate B. Then, the Co(iii) coordinates with trimethyl(phenylethynyl)silane followed by insertion to generate species C, which undergoes the reductive elimination to enable the formation of the trimethylsilyl substituted isoquinolone 7a and liberate the Co(i) species. The oxidation of Co(i) to Co(iii) by Mn^II^ or Mn^III^ and O_2_ fulfills the catalytic cycle. The removal of the trimethylsilyl group can be accomplished to give the isoquinolone 3a under the reaction condition (Scheme 3, eqn (6)). In addition, the replacement of terminal alkyne with alkynylsilane as a coupling partner under standard conditions was also performed and give rise to the product 3a in 55% yield, revealed that the terminal alkyne was also reactive. Herein, the first desilylation step of the reaction could not be excluded (Scheme 3, eqn (7)).

**Scheme 4:**
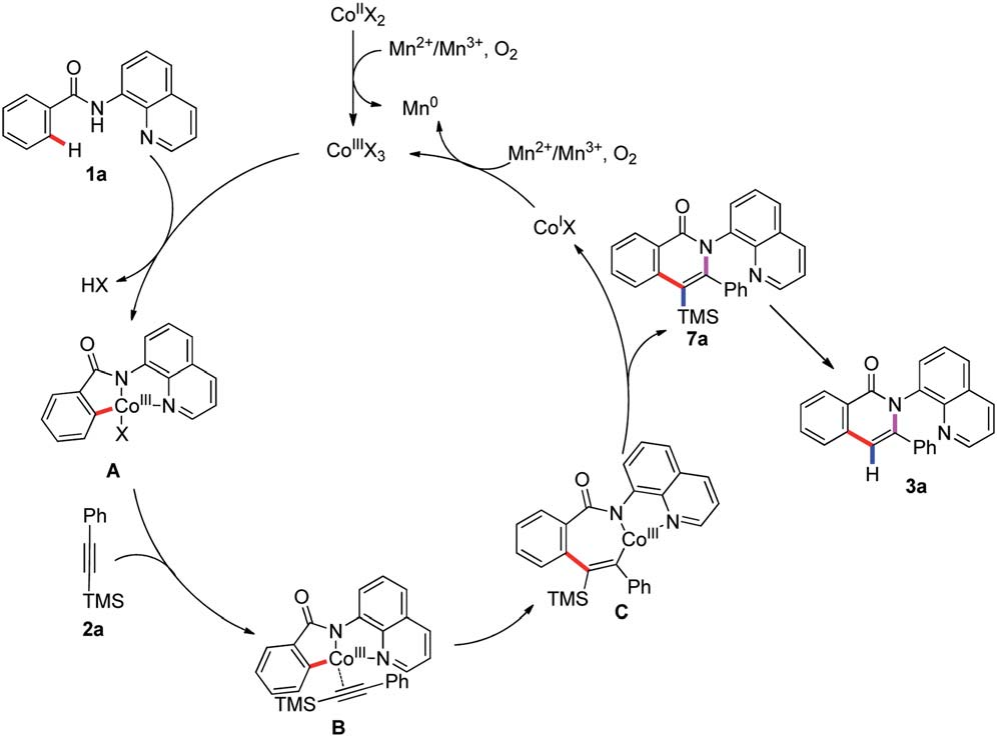
Proposed reaction mechanism.

In summary, we have developed a highly efficient approach for the direct cobalt catalyzed annulation of arenes and alkenes with alkynylsilanes using 8-aminoquinolyl auxiliary as directing group. Notable features of this protocol include broad substrate scopes, excellent regioselectivity and good functional group compatibility. More significantly, the trimethylsilyl group in the final products, including isoquinolones and pyridones, can be readily removed and retained by using different catalytic systems.

## Conflicts of interest

There are no conflicts to declare.

## Supplementary Material

RA-009-C9RA06963A-s001
