# Supplementary material for: Co-catalyzed ortho-C–H functionalization/annulation of arenes and alkenes with alkynylsilanes: access to isoquinolone and pyridone motifs
Source: RSC Adv. 2019 Sep 26;9(53):30650–4. doi: 10.1039/c9ra06963a (PMC9072155; doi:10.1039/c9ra06963a)

Electronic Supplementary Material (ESI) for RSC Advances.
This journal is © The Royal Society of Chemistry 2019

Electronic Supplementary Information

Co-Catalyzed *ortho*-C-H Functionalization/ Annulation of Arenes and Alkenes with Alkynylsilanes: Access to Isoquinolone and Pyridone Motifs

Cong Lin, *[a,b] and Liang Shen*[a,b]

[a] *College of Chemistry and Chemical Engineering, Jiangxi Science & Technology Normal University, Nanchang 330013, China*

[b] *College of Chemistry and Chemical Engineering, Jiangxi Engineering Laboratory of Waterborne Coatings, Jiangxi Science & Technology Normal University, Nanchang 330013, China*

E-mail: *conglin0127@jxstnu.com.cn*

Table of Contents

General Information....S2
Optimization of Reaction Conditions[a]....S3
Typical Procedure for the Synthesis of Isoquinolones....S4
Typical Procedure for the Synthesis of Timethylsilane Isoquinolones....S4
Analytical Data for Products....S5
Gram-Scale Synthesis of 3a and 7a....S26
The H/D Exchange Experiments....S27
The Kinetic Isotope Experiments....S28
References....S30
Copies of ^{1}H and ^{13}C NMR Spectra....S31

General Information

All reactions were performed under O_2 atmosphere in a 25 mL sealed tube. The materials and solvents were purchased from common commercial sources and used without additional purification, if there is no special version. Starting materials were synthesized according to literature procedures.[1-7] ^{1}H NMR spectra were recorded at 400 MHz using TMS as internal standard, ^{13}C NMR spectra was recorded at 100 MHz using TMS as internal standard. The multiplicities are reported as follows: singlet (s), doublet (d), doublet of doublets (dd), multiplet (m), and andtriplet (t). Mass spectroscopy data of the products were collected on an HRMS-TOF instrument.

Optimization of Reaction Conditions[a]

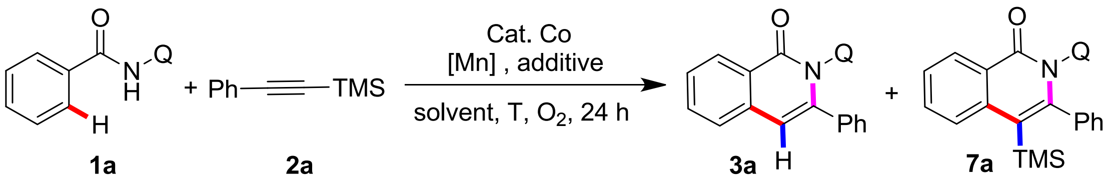

Entry	Cat.Co	[Mn]	additive	solvent	T(°C)	**3a**[b]	**7a**[b]
1	$Co(OAc)_2$	$Mn(OAc)_2$	CsF	TFE	100	85%	<5%
2	$CoCl_2$	$Mn(OAc)_2$	CsF	TFE	100	63%	<5%
3	CoI_2	$Mn(OAc)_2$	CsF	TFE	100	30%	15%
4	$CoBr_2$	$Mn(OAc)_2$	CsF	TFE	100	34%	11%
**5**	**$Co(OAc)_2·4H_2O$**	**$Mn(OAc)_2$**	**CsF**	**TFE**	**100**	**96%**	**trace**
6	$Co(acac)_2$	$Mn(OAc)_2$	CsF	TFE	100	55%	26%
7	$Co(acac)_3$	$Mn(OAc)_2$	CsF	TFE	100	57%	20%
8	-	$Mn(OAc)_2$	CsF	TFE	100	0	0
9	$Co(OAc)_2·4H_2O$	$Mn(OAc)_2$	CsF, NaOAc(2.0)	TFE	100	73%	15%
10	$Co(OAc)_2·4H_2O$	$Mn(OAc)_2$	CsF(2.0)	TFE	100	83%	trace
11	$Co(OAc)_2·4H_2O$	$Mn(OAc)_2$	CsF(4.0)	TFE	100	77%	trace
12	$Co(OAc)_2·4H_2O$	$Mn(OAc)_2$	KF	TFE	100	75%	10%
13	$Co(OAc)_2·4H_2O$	$Mn(OAc)_2$	LiF	TFE	100	22%	trace
14	$Co(OAc)_2·4H_2O$	$Mn(OAc)_2$	NaF	TFE	100	21%	trace
15	$Co(OAc)_2·4H_2O$	$Mn(OAc)_2$	-	TFE	100	33%	21%
16[c]	$Co(OAc)_2·4H_2O$	$Mn(OAc)_2$	CsF	TFE	100	0	0
17[d]	$Co(OAc)_2·4H_2O$	$Mn(OAc)_2$	CsF	TFE	100	21%	<10%
18	$Co(OAc)_2·4H_2O$	$Mn(OAc)_2$	CsF	TFE	80	trace	trace
19	$Co(OAc)_2·4H_2O$	$Mn(OAc)_2$	CsF	TFE	90	66%	15%
20	$Co(OAc)_2·4H_2O$	$Mn(OAc)_2$	CsF	TFE	110	72%	<10%
21	$Co(OAc)_2·4H_2O$	$Mn(OAc)_2$	CsF	DCE	100	0	0
22	$Co(OAc)_2·4H_2O$	$Mn(OAc)_2$	CsF	HFIP	100	0	0
23	$Co(OAc)_2·4H_2O$	$Mn(OAc)_2$	CsF	DMF	100	0	0
24	$Co(OAc)_2·4H_2O$	$Mn(OAc)_2$	CsF	Dioxane	100	0	0
25	$Co(OAc)_2·4H_2O$	$Mn(OAc)_2$	CsF	$PhCF_3$	100	0	0
26	$Co(OAc)_2·4H_2O$	$Mn(OAc)_2·4H_2O$	CsF	TFE	100	83%	trace
27	$Co(OAc)_2·4H_2O$	$Mn(OAc)_3·2H_2O$	CsF	TFE	100	56%	11%
28	$Co(OAc)_2·4H_2O$	$Mn(acac)_2$	CsF	TFE	100	23%	39%
29	$Co(OAc)_2·4H_2O$	$Mn(acac)_3$	CsF	TFE	100	18%	56%
30	$Co(OAc)_2·4H_2O$	-	CsF	TFE	100	trace	trace
**31**	**$Co(acac)_2$**	**$Mn(acac)_3$**	**NaOAc (0.2)**	**TFE**	**100**	**<5**	**76**
32	$Co(acac)_3$	$Mn(acac)_3$	NaOAc (0.2)	TFE	100	trace	trace

[a] Reactions were carried out by using **1a** (0.1 mmol), **2a** (0.3 mmol), Cat. Co (0.02 mmol), [Mn] (0.2 mmol), additive (0.12 mmol), solvent (1.0 ml), T, O_2, 24 h. [b] Isolated yield. [c] N_2. [d] air.

Typical Procedure for the Synthesis of Isoquinolones

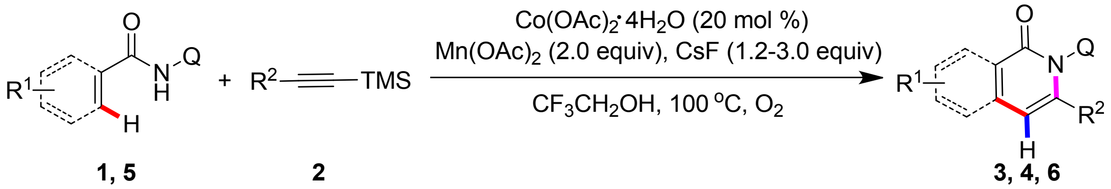

A 25 mL sealed tube was charged with amide **1, 5** (0.1 mmol), alkynylsilanes **2** (0.3 mmol), $Co(OAc)_2·4H_2O$ (5 mg, 0.02 mmol), $Mn(OAc)_2$ (34.6 mg, 0.2 mmol), CsF (18.2-45.6 mg, 0.12-0.3 mmol) and CF_3CH_2OH (1.0 mL). The vial was evacuated and filled with O_2 atmosphere for five times, and stirred at 100 °C or 120 °C for 24 h. The mixture was then cooled to room temperature, diluted with EtOAc, filtered through a celite pad, and concentrated in vacuo. The residue was purified by flash column chromatography on silica gel, eluting with EtOAc/hexane (1:5 ~ 1:2, v/v), to afford the desired products **3, 4** and **6**.

Typical Procedure for the Synthesis of Timethylsilane Isoquinolones

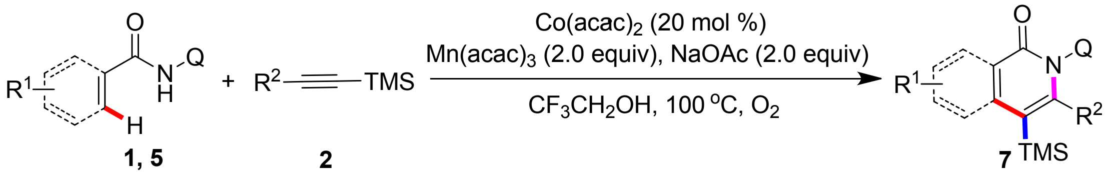

A 25 mL sealed tube was charged with amide **1, 5** (0.1 mmol), alkynylsilanes **2** (0.3 mmol), $Co(acac)_2$ (5.2 mg, 0.02 mmol), $Mn(acac)_3$ (70.4 mg, 0.2 mmol), NaOAc (16.4 mg, 0.2 mmol) and CF_3CH_2OH (1.0 mL). The vial was evacuated and filled with O_2 atmosphere for five times, and stirred at 100 °C for 24 h. The mixture was then cooled to room temperature, diluted with EtOAc, filtered through a celite pad, and concentrated in vacuo. The residue was purified by flash column chromatography on silica gel, eluting with EtOAc/hexane (1:5 ~ 1:2, v/v), to afford the desired products **7**.

Analytical Data for Products

3-phenyl-2-(quinolin-8-yl)isoquinolin-1(*2H*)-one (3a)

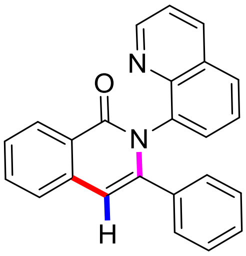

Rf 0.24 (hexane/EtOAc = 2/1). 96%, 33.4 mg. White solid; ^{1}H NMR ($CDCl_3$, 400 MHz) δ 8.91 (dd, J_1 = 1.6 Hz; J_2 = 4.0 Hz, 1H), 8.47 (d, J = 8.0 Hz, 1H), 8.09 (dd, J_1 = 1.6 Hz; J_2 = 8.4 Hz, 1H), 7.68-7.30 (m, 2H), 7.61 (d, J = 7.6 Hz, 1H), 7.48-7.53 (m, 2H), 7.41 (t, J = 8.0 Hz, 1H), 7.37 (dd, J_1 = 4.0 Hz; J_2 = 8.0 Hz, 1H), 7.09-7.12 (m, 2H), 7.00-7.04 (m, 1H), 6.95 (t, J = 7.2 Hz, 2H), 6.66 (s, 1H).^{13}C NMR ($CDCl_3$, 100 MHz) δ 163.3, 151.0, 144.7, 144.5, 137.3, 137.3, 136.3, 136.2, 132.7, 130.8, 128.8, 128.7, 128.5, 128.0, 127.4, 126.8, 126.2, 125.9, 125.5, 121.6, 107.6. HRMS (EI-TOF) calcd for$C_{24}H_{16}N_2O$ (M^+):348.1263, found:348.1263.

3-(4-bromophenyl)-2-(quinolin-8-yl)isoquinolin-1(*2H*)-one (3b)

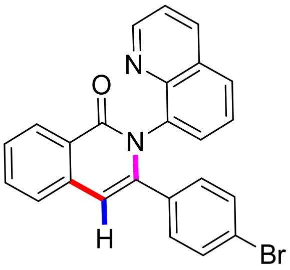

Rf 0.24 (hexane/EtOAc = 2/1). 58%, 24.7 mg. White solid; ^{1}H NMR ($CDCl_3$, 400 MHz) δ 8.89 (dd, J_1 = 1.6 Hz; J_2 = 4.0 Hz, 1H), 8.46 (d, J = 8.0 Hz, 1H), 8.12 (dd, J_1 = 1.6 Hz; J_2 = 8.4 Hz, 1H), 7.77 (dd, J_1 = 1.2 Hz; J_2 = 8.0 Hz, 1H), 7.69-7.73 (m, 1H), 7.60 (d, J= 7.6 Hz, 1H), 7.50-7.54 (m, 2H), 7.46 (t, J= 7.6 Hz, 1H), 7.39 (dd, J_1 = 4.4 Hz; J_2 = 8.4 Hz, 1H), 7.09 (d, J= 7.6 Hz, 2H), 7.00 (d, J= 8.8 Hz, 2H), 6.62 (s, 1H). ^{13}C NMR ($CDCl_3$, 100 MHz) δ 163.2, 151.0, 144.6, 143.3, 137.1, 137.0, 136.3, 135.3, 132.8, 130.8, 130.6, 130.4, 129.0, 128.9, 128.5, 127.0, 126.2, 126.0, 125.6, 122.3, 121.7, 107.7. HRMS (EI-TOF) calcd for$C_{24}H_{15}BrN_2O$ (M^+):426.0368,

found:426.0366

.

3-(pyridin-2-yl)-2-(quinolin-8-yl)isoquinolin-1(*2H*)-one (**3c**)

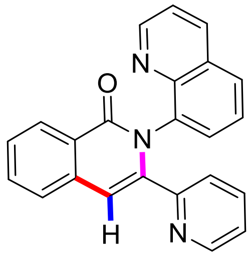

Rf 0.24 (hexane/EtOAc = 10/1). 91%, 31.8 mg. White solid; ^{1}H NMR ($CDCl_3$, 400 MHz) δ 8.90-8.91 (m, 1H), 8.49 (d, *J* = 8.0 Hz, 1H), 8.31 (d, *J* = 4.4 Hz, 1H), 8.11 (d, *J* = 8.4 Hz, 1H), 7.70-7.75 (m, 2H), 7.65 (d, *J* = 9.6 Hz, 2H), 7.52-7.56 (m, 1H), 7.45 (t, *J* = 8.0 Hz, 1H), 7.37 (dd, J_1 = 4.0 Hz; J_2 = 8.0 Hz, 1H), 7.20-7.24 (m, 1H), 7.08 (d, *J* = 7.6 Hz, 1H), 6.93 (dd, J_1 = 5.2 Hz; J_2 = 7.2 Hz, 1H), 6.86 (s, 1H). ^{13}C NMR ($CDCl_3$, 100 MHz) δ 163.2, 154.2, 150.9, 148.5, 144.5, 142.9, 137.0, 136.9, 136.3, 135.6, 132.8, 131.1, 128.7, 128.5, 127.2, 126.6, 126.0, 123.9, 122.6, 121.5, 108.4. HRMS (EI-TOF) calcd for $C_{23}H_{15}N_3O$ (M^+): 349.1215, found: 349.1211.

3-cyclohexyl-2-(quinolin-8-yl)isoquinolin-1(*2H*)-one (**3d**)

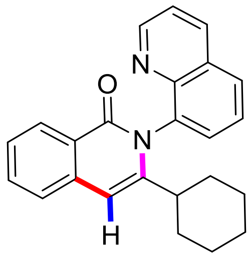

Rf 0.24 (hexane/EtOAc = 10/1). 98%, 34.7 mg. White solid; ^{1}H NMR ($CDCl_3$, 400 MHz) δ 8.88 (dd, J_1 = 1.6 Hz; J_2 = 4.0 Hz, 1H), 8.39 (d, *J* = 8.0 Hz, 1H), 8.24 (dd, J_1 = 2.0 Hz; J_2 = 8.0 Hz, 1H), 7.95 (dd, J_1 = 1.6 Hz; J_2 = 8.0 Hz, 1H), 7.70-7.75 (m, 2H), 7.61-7.68 (m, 1H), 7.52 (d, *J* = 8.0 Hz, 1H), 7.40-7.45 (m, 2H), 6.43 (s, 1H), 1.16-1.26 (m, 5H), 0.84-0.96 (m, 2H), 0.64-0.71 (m, 1H), 0.56-0.63 (m, 1H), 0.45-0.51 (m, 1H), 0.27-0.34 (m, 1H). ^{13}C NMR ($CDCl_3$, 100 MHz) δ 163.7, 151.3, 145.2, 144.7, 137.6, 137.1, 136.3, 132.5, 130.3, 129.3, 129.0, 128.3, 126.3, 126.1, 125.7, 125.2, 121.7, 103.2, 31.5, 30.2, 29.7. HRMS (EI-TOF) calcd for $C_{24}H_{22}N_3O$ (M^+): 354.1732, found:

354.1735.

3-methyl-2-(quinolin-8-yl)isoquinolin-1(*2H*)-one (3e)

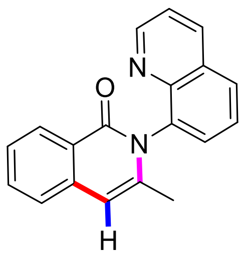

Rf 0.24 (hexane/EtOAc = 10/1). 56%, 16.0 mg. White solid; ^{1}H NMR ($CDCl_3$, 400 MHz) δ 8.89 (dd, J_1 = 1.6 Hz; J_2 = 4.4 Hz, 1H), 8.38 (d, J = 8.0 Hz, 1H), 8.24 (dd, J_1 = 1.6 Hz; J_2 = 5.2 Hz, 1H), 7.97 (dd, J_1 = 2.4 Hz; J_2 = 6.4 Hz, 1H), 7.62-7.76 (m, 3H), 7.51-7.55 (m, 1H), 7.44 (dd, J_1 = 4.0 Hz; J_2 = 8.4 Hz, 2H), 6.54 (s, 1H), 1.93 (s, 3H). ^{13}C NMR ($CDCl_3$, 100 MHz) δ 161.8, 149.5, 134.4, 130.5, 130.3, 128.0, 127.2, 127.1, 126.3, 125.1, 124.4, 124.4, 123.9, 123.3, 120.9, 119.9, 119.7, 103.5, 19.1. HRMS (EI-TOF) calcd for $C_{19}H_{14}N_2O$ (M^+): 286.1106, found: 286.1100.

2-(quinolin-8-yl)-3-(trimethylsilyl)isoquinolin-1(*2H*)-one (3f)

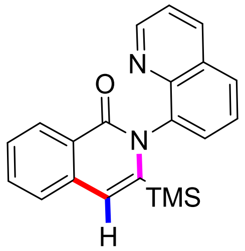

Rf 0.24 (hexane/EtOAc = 10/1). 64%, 22.0 mg. White solid; ^{1}H NMR ($CDCl_3$, 400 MHz) δ 8.88 (dd, J_1 = 1.6 Hz; J_2 = 4.0 Hz, 1H), 8.42 (d, J = 8.0 Hz, 1H), 8.22 (dd, J_1 = 1.6 Hz; J_2 = 8.4 Hz, 1H), 7.97 (dd, J_1 = 1.6 Hz; J_2 = 8.0 Hz, 1H), 7.65-7.73 (m, 3H), 7.59 (d, J = 8.0 Hz, 1H), 7.49-7.53 (m, 1H), 7.42 (dd, J_1 = 4.0 Hz; J_2 = 8.0 Hz, 1H), 6.91 (s, 1H), -0.27 (s, 9H). ^{13}C NMR ($CDCl_3$, 100 MHz) δ 163.9, 151.5, 147.0, 145.7, 139.5, 137.1, 136.4, 132.6, 130.7, 129.7, 129.4, 128.2, 127.5, 126.6, 126.4, 126.2, 122.1, 115.7, 0.02. HRMS (EI-TOF) calcd for $C_{21}H_{20}N_2OSi$ (M^+): 344.1345, found: 344.1345.

8-methyl-3-phenyl-2-(quinolin-8-yl)isoquinolin-1(2*H*)-one (**4a**)

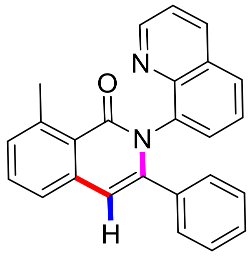

Rf 0.39 (hexane/EtOAc = 5/1). 80%, 29.0 mg. White solid; ^{1}H NMR ($CDCl_3$, 400 MHz) δ 8.91 (dd, J_1 = 1.6 Hz; J_2 = 4.0 Hz, 1H), 8.07 (dd, J_1 = 1.6 Hz; J_2 = 8.0 Hz, 1H), 7.70 (dd, J_1 = 1.2 Hz; J_2 = 8.0 Hz, 1H), 7.50-7.54 (m, 2H), 7.40-7.44 (m, 2H), 7.35 (dd, J_1= 4.0 Hz; J_2 = 8.0 Hz, 1H), 7.24-7.26 (m, 1H), 7.09-7.11 (m, 2H), 6.98-7.00 (m, 1H), 6.91-6.95 (m, 2H), 6.58 (s, 1H), 2.90 (s, 3H). ^{13}C NMR ($CDCl_3$, 100 MHz) δ 164.1, 150.9, 144.9, 144.3, 142.5, 139.1, 137.8, 136.3, 136.1, 132.0, 130.9, 129.9, 128.9, 128.7, 128.5, 127.9, 127.3, 125.9, 124.6, 123.9, 121.6, 107.9, 24.0. HRMS (EI-TOF) calcd for $C_{25}H_{18}N_2O$ (M^+): 362.1419, found: 362.1413.

7-methyl-3-phenyl-2-(quinolin-8-yl)isoquinolin-1(2*H*)-one (**4b**)

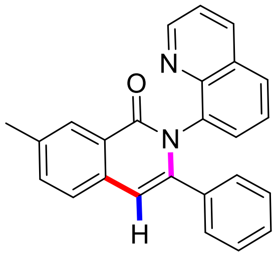

Rf 0.39 (hexane/EtOAc = 5/1). 61%, 22.1 mg. White solid; ^{1}H NMR ($CDCl_3$, 400 MHz) δ 8.90 (dd, J_1 = 1.6 Hz; J_2 = 4.0 Hz, 1H), 8.27 (s, 1H), 8.08 (dd, J_1 = 1.6 Hz; J_2 = 8.0 Hz, 1H), 7.70-7.72 (m, 1H), 7.51 (s, 2H), 7.42-7.49 (m, 1H), 7.40 (d, J = 8.0 Hz, 1H), 7.35 (dd, J_1= 4.0 Hz; J_2 = 8.0 Hz, 1H), 7.08-7.12 (m, 2H), 6.99-7.03 (m, 1H), 6.93 (t, J = 7.2 Hz, 2H), 6.62 (s, 1H), 2.50 (s, 3H). ^{13}C NMR ($CDCl_3$, 100 MHz) δ 161.0, 148.7, 148.7, 142.6, 141.3, 135.2, 134.5, 134.2, 133.9, 132.7, 131.9, 128.5, 126.6, 126.4, 125.8, 125.6, 125.0, 123.9, 123.6, 123.2, 119.3, 105.2, 19.4. HRMS (EI-TOF) calcd for $C_{25}H_{18}N_2O$ (M^+): 362.1419, found: 362.1412.

6-methyl-3-phenyl-2-(quinolin-8-yl)isoquinolin-1(2*H*)-one (**4c**)

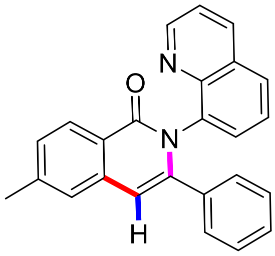

Rf 0.26 (hexane/EtOAc = 5/1). 69%, 25.0 mg. Yellow solid; ^{1}H NMR ($CDCl_3$, 400 MHz) δ 8.91 (dd, J_1 = 1.6 Hz; J_2 = 4.0 Hz, 1H), 8.36 (d, J = 8.0 Hz, 1H), 8.09 (dd, J_1 = 1.6 Hz; J_2 = 8.4 Hz, 1H), 7.71 (dd, J_1 = 1.2 Hz; J_2 = 8.0 Hz, 1H), 7.45-7.49 (m, 1H), 7.38-7.43 (m, 2H), 7.32-7.37 (m, 2H), 7.10 (d, J = 7.2 Hz, 2H), 7.00-7.03 (m, 1H), 6.92-6.96 (m, 2H), 6.58 (s, 1H), 2. 52 (s, 3H). ^{13}C NMR ($CDCl_3$, 100 MHz) δ 163.3, 150.9, 144.8, 144.5, 143.2, 137.4, 136.4, 136.1, 130.8, 128.8, 128.6, 128.4, 128.4, 127.9, 127.3, 126.7, 126.2, 125.9, 125.8, 123.3, 121.5, 107.4, 21.9. HRMS (EI-TOF) calcd for $C_{25}H_{18}N_2O$ (M^+): 362.1419, found: 362.1419.

6-methoxy-3-phenyl-2-(quinolin-8-yl)isoquinolin-1(*2H*)-one (**4d**)

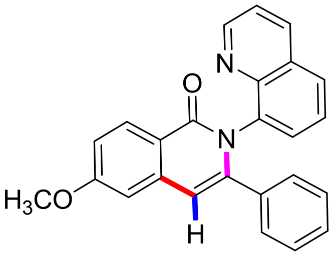

Rf 0.15 (hexane/EtOAc = 10/1). 84%, 31.8 mg.White solid; ^{1}H NMR ($CDCl_3$, 400 MHz) δ 8.91 (dd, J_1 = 1.6 Hz; J_2 = 4.4 Hz, 1H), 8.38 (d, J = 8.8 Hz, 1H), 8.07 (dd, J_1 = 1.6 Hz; J_2 = 4.4 Hz, 1H), 7.70 (dd, J_1 = 1.2 Hz; J_2 = 8.4 Hz, 1H), 7.48 (dd, J_1 = 1.6 Hz; J_2 = 7.2 Hz, 1H), 7.34-7.42 (m, 2H), 7.06-7.11 (m, 3H), 7.01 (t, J = 7.6 Hz, 1H), 6.92-6.96 (m, 3H), 6.57 (s, 1H), 3.93 (s, 3H). ^{13}C NMR ($CDCl_3$, 100 MHz) δ 163.2, 163.0, 150.9, 145.1, 144.8, 139.3, 137.3, 136.4, 136.2, 130.9, 130.6, 128.8, 128.8, 128.7, 128.0, 127.3, 125.8, 121.5, 119.3, 116.2, 107.4, 107.0, 55.6. HRMS (EI-TOF) calcd for $C_{25}H_{18}N_2O_2$ (M^+): 378.1368, found: 378.1365.

6-(tert-butyl)-3-phenyl-2-(quinolin-8-yl)isoquinolin-1(*2H*)-one (**4e**)

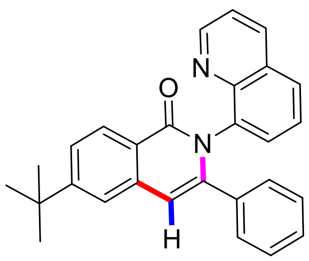

Rf 0.12 (hexane/EtOAc = 10/1). 46%, 18.6 mg. White solid; ^{1}H NMR ($CDCl_3$, 400 MHz) δ 8.89 (dd, J_1 = 1.6 Hz; J_2 = 4.0 Hz, 1H), 8.40 (d, J = 7.6 Hz, 1H), 8.08 (dd, J_1 = 1.6 Hz; J_2 = 8.4 Hz, 1H), 7.71 (dd, J_1 = 1.6 Hz; J_2 = 8.0 Hz, 1H), 7.57-7.59 (m, 2H), 7.45-7.50 (m, 1H), 7.39-7.43 (m, 1H), 7.36 (dd, J_1 = 4.0 Hz; J_2 = 8.4 Hz, 1H), 7.09-7.11 (m, 2H), 7.01 (d, J = 7.6 Hz, 1H), 6.92-6.96 (m, 2H), 6.95 (s, 1H), 1.42 (s, 9H). ^{13}C NMR ($CDCl_3$, 100 MHz) δ 163.2, 156.2, 150.9, 144.4, 137.2, 136.5, 136.1, 130.8, 130.0, 128.8, 128.8, 128.6, 128.6, 128.2, 127.9, 127.3, 125.8, 124.9, 123.2, 122.2, 121.5, 107.8, 35.2, 31.2. HRMS (EI-TOF) calcd for $C_{28}H_{24}N_2O$ (M^+): 404.1889, found: 404.1889.

6,7-dimethoxy-3-phenyl-2-(quinolin-8-yl)isoquinolin-1(*2H*)-one (4f)

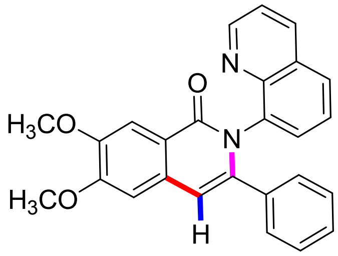

Rf 0.13 (hexane/EtOAc = 10/1). 74%, 30.2 mg. White solid; ^{1}H NMR ($CDCl_3$, 400 MHz) δ 8.92 (dd, J_1 = 1.6 Hz; J_2 = 4.0 Hz, 1H), 8.10 (dd, J_1 = 1.6 Hz; J_2 = 8.0 Hz, 1H), 7.87 (s, 1H), 7.72 (dd, J_1 = 1.6 Hz; J_2 = 8.0 Hz, 1H), 7.42-7.47 (m, 2H), 7.36-7.40 (m, 2H), 7.08-7.10 (m, 2H), 7.02 (t, J = 7.2 Hz, 1H), 6.93-6.97 (m, 2H), 6.59 (s, 1H), 4.02 (s, 3H), 3.99 (s, 3H). ^{13}C NMR ($CDCl_3$, 100 MHz) δ 160.3, 151.6, 148.7, 142.7, 140.9, 135.3, 134.2, 133.9, 130.5, 128.4, 131.9, 126.6, 126.5, 126.4, 125.6, 125.1, 123.6, 119.2, 117.1, 106.1, 104.8, 104.1, 53.9, 53.9. HRMS (EI-TOF) calcd for $C_{26}H_{20}N_2O_3$ (M^+): 408.1474, found: 408.1468.

7-iodo-3-phenyl-2-(quinolin-8-yl)isoquinolin-1(*2H*)-one (4g)

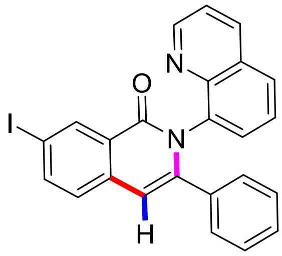

Rf 0.12 (hexane/EtOAc = 10/1). 65%, 30.8 mg. White solid; ^{1}H NMR ($CDCl_3$, 400 MHz) δ 8.90 (dd, J_1 = 1.6 Hz; J_2 = 4.0 Hz, 1H), 8.80 (d, J = 1.6 Hz, 1H), 8.10 (dd, J_1 = 1.6 Hz; J_2 = 8.4 Hz, 1H), 7.96 (dd, J_1 = 1.6 Hz; J_2 = 8.4 Hz, 1H), 7.73 (dd, J_1 = 1.2 Hz; J_2 = 8.0 Hz, 1H), 7.33-7.48 (m, 4H), 7.13 (d, J = 8.4 Hz, 2H), 7.01-7.09 (m, 1H), 6.95 (t, J = 7.6 Hz, 2H), 6.59 (s, 1H). ^{13}C NMR ($CDCl_3$, 100 MHz) δ 161.8, 150.9, 145.4, 144.5, 141.3, 137.3, 137.0, 136.4, 136.2, 136.0, 130.6, 128.9, 128.8, 128.7, 128.2, 127.8, 127.4, 127.0, 125.9, 121.6, 106.9, 91.2. HRMS (EI-TOF) calcd for $C_{24}H_{15}IN_2O$ (M^+): 474.0229, found: 474.0227.

7-bromo-3-phenyl-2-(quinolin-8-yl)isoquinolin-1(*2H*)-one (4h)

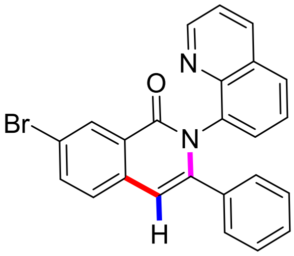

Rf 0.12 (hexane/EtOAc = 10/1). 38%, 16.2 mg. White solid; ^{1}H NMR ($CDCl_3$, 400 MHz) δ 8.91 (dd, J_1 = 1.6 Hz; J_2 = 4.0 Hz, 1H), 8.26 (d, J = 8.0 Hz, 1H), 8.10 (dd, J_1 = 1.6 Hz; J_2 = 8.0 Hz, 1H), 7.74 (dd, J_1 = 1.6 Hz; J_2 = 8.0 Hz, 1H), 7.37-7.50 (m, 5H), 7.10-7.12 (m, 2H), 7.02-7.06 (m, 1H), 6.94-6.98 (m, 2H), 6.87 (s, 1H). ^{13}C NMR ($CDCl_3$, 100 MHz) δ 159.2, 151.0, 145.2, 144.6, 137.1, 136.1, 136.1, 130.6, 128.9, 128.8, 128.2, 127.4, 126.8, 126.8, 125.8, 124.1, 124.1, 121.6, 117.6, 117.4, 99.9, 99.8. HRMS (EI-TOF) calcd for $C_{24}H_{15}BrN_2O$ (M^+): 426.0368, found: 426.0367.

6-chloro-3-phenyl-2-(quinolin-8-yl)isoquinolin-1(*2H*)-one (4i)

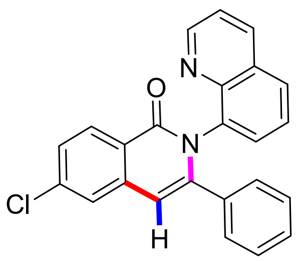

Rf 0.12 (hexane/EtOAc = 10/1). 78%, 29.8 mg. White solid; ^{1}H NMR ($CDCl_3$, 400 MHz) δ 8.92 (dd, J_1 = 1.6 Hz; J_2 = 4.0 Hz, 1H), 8.40 (d, J = 8.8 Hz, 1H), 8.10 (dd, J_1 = 1.6 Hz; J_2 = 8.4 Hz, 1H), 7.74 (dd, J_1 = 1.6 Hz; J_2 = 8.0 Hz, 1H), 7.59 (d, J = 2.0 Hz, 1H), 7.38-7.50 (m, 4H), 7.09-7.11 (m, 2H), 7.02-7.06 (m, 1H), 6.96 (t, J = 7.2 Hz, 2H), 6.57 (s, 1H). ^{13}C NMR ($CDCl_3$, 100 MHz) δ 162.7, 151.0, 146.0, 144.6, 139.1, 138.5, 137.0, 136.2, 135.9, 130.7, 130.3, 128.9, 128.8, 128.7, 128.2, 127.4, 127.2, 125.9, 125.4, 123.8, 121.6, 106.4. HRMS (EI-TOF) calcd for $C_{24}H_{15}ClN_2O$ (M^+): 382.0873, found: 382.0873.

7-chloro-3-phenyl-2-(quinolin-8-yl)isoquinolin-1(*2H*)-one (**4j**)

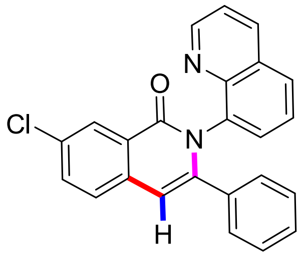

Rf 0.12 (hexane/EtOAc = 10/1). 49%, 18.7 mg. White solid; ^{1}H NMR ($CDCl_3$, 400 MHz) δ 8.91 (dd, J_1 = 1.6 Hz; J_2 = 4.0 Hz, 1H), 8.44 (d, J = 2.0 Hz, 1H), 8.10 (dd, J_1 = 1.6 Hz; J_2 = 8.4 Hz, 1H), 7.73 (dd, J_1 = 1.2 Hz; J_2 = 8.0 Hz, 1H), 7.64 (dd, J_1 = 2.0 Hz; J_2 = 7.6 Hz, 1H), 7.55 (d, J = 8.4 Hz, 1H), 7.37-7.49 (m, 3H), 7.07-7.09 (m, 2H), 7.03 (t, J = 7.6 Hz, 1H), 6.95 (t, J = 7.6 Hz, 2H), 6.62 (s, 1H). ^{13}C NMR ($CDCl_3$, 100 MHz) δ 162.2, 151.0, 144.9, 144,6, 137.0, 136.2, 136.0, 135.7, 133.2, 132.6, 130.7, 128.9, 128.8, 128.7, 128.2, 127.9, 127.8, 127.4, 126.6, 125.9, 121.6, 106.8. HRMS (EI-TOF) calcd for $C_{24}H_{15}ClN_2O$ (M^+): 382.0873, found: 382.0870.

8-chloro-3-phenyl-2-(quinolin-8-yl)isoquinolin-1(*2H*)-one (**4k**)

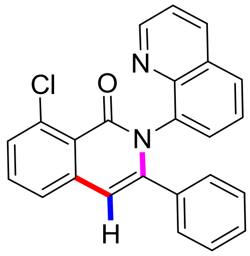

Rf 0.12 (hexane/EtOAc = 10/1). 41%, 15.7 mg. White solid; ^{1}H NMR ($CDCl_3$, 400 MHz) δ 8.91 (dd, J_1 = 1.6 Hz; J_2 = 4.0 Hz, 1H), 8.09 (dd, J_1 = 1.2 Hz; J_2 = 8.4 Hz, 1H), 7.72 (dd, J_1 = 1.2 Hz; J_2 = 8.0 Hz, 1H), 7.36-7.53 (m, 6H), 7.10-7.12 (m, 2H), 7.02 (d, J = 7.2 Hz, 1H), 6.93-6.97 (m, 2H), 6.59 (s, 1H). ^{13}C NMR ($CDCl_3$, 100 MHz) δ 161.5, 150.9, 145.5, 144.6, 140.4, 140.0, 137.1, 136.1, 135.9, 132.4, 131.0, 129.9. 128.9, 128.7, 128.6, 128.2, 127.4, 125.9, 125.5, 121.9, 121.6, 107.0. HRMS (EI-TOF) calcd for $C_{24}H_{15}ClN_2O$ (M^+): 382.0873, found: 382.0873.

5-fluoro-3-phenyl-2-(quinolin-8-yl)isoquinolin-1(*2H*)-one (4l)

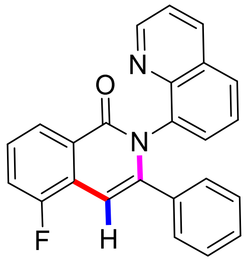

Rf 0.12 (hexane/EtOAc = 10/1). 51%, 18.7 mg. White solid; ^{1}H NMR ($CDCl_3$, 400 MHz) δ 8.91 (dd, J_1 = 1.6 Hz; J_2 = 4.4 Hz, 1H), 8.26 (d, J = 7.6 Hz, 1H), 8.10 (dd, J_1 = 1.6 Hz; J_2 = 8.0 Hz, 1H), 7.74 (dd, J_1 = 1.6 Hz; J_2 = 8.0 Hz, 1H), 7.37-7.50 (m, 5H), 7.10-7.12 (m, 2H), 7.02-7.04 (m, 1H), 6.94 (t, J = 7.2 Hz, 2H), 6.87 (s, 1H). ^{13}C NMR ($CDCl_3$, 100 MHz) δ 162.4, 162.3, 157.9 (d, $J_{C\text{-}F}$ = 250.1 Hz), 151.0, 144.9 (d, $J_{C\text{-}F}$ = 65.5 Hz), 137.0, 136.2, 136.1, 130.7, 128.9, 128.8, 128.2. 127.4, 127.2, 127.1, 126.8 (d, $J_{C\text{-}F}$ = 7.3 Hz), 125.9, 124.1, 124.1, 121.6, 117.5 (d, $J_{C\text{-}F}$ = 20.3 Hz), 99.8 (d, $J_{C\text{-}F}$ = 5.3 Hz). HRMS (EI-TOF) calcd for $C_{24}H_{15}FN_2O$ (M^+): 366.1168, found: 366.1160.

According to the ^{1}H and ^{13}C NMRs, there is no single peak in aromatic area of ^{1}H NMR, herein, the C-H activation occur at the *ortho*-position to F.

3-phenyl-2-(quinolin-8-yl)benzo[*h*]isoquinolin-1(*2H*)-one (**4m**)

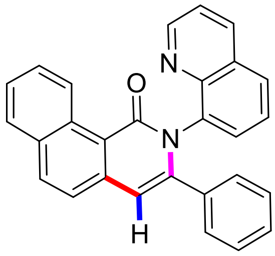

Rf 0.12 (hexane/EtOAc = 10/1). 45%, 17.9 mg. White solid; ^{1}H NMR ($CDCl_3$, 400 MHz) δ 10.12 (d, *J* = 8.8 Hz, 1H), 8.98-9.04 (m, 2H), 8.12-8.16 (m, 2H), 8.02-8.07 (m, 2H), 7.87-7.92 (m, 2H), 7.36-7.60 (m, 3H), 7.03-7.18 (m, 3H), 6.98 (t, *J* = 7.6 Hz, 2H), 6.80 (s, 1H). ^{13}C NMR ($CDCl_3$, 100 MHz) δ 163.7, 151.0, 147.6, 147.1, 145.8, 139.2, 134.3, 134.1, 136.5, 132.5, 131.0, 128.8, 128.6, 128.4, 128.2, 127.9, 127.4, 126.2, 126.1, 126.0, 125.0, 124.5, 124.0, 121.7, 119.1, 108.3. HRMS (EI-TOF) calcd for $C_{28}H_{18}N_2O$ (M^+): 398.1419, found: 398.1422.

3-phenyl-2-(quinolin-8-yl)benzo[*g*]isoquinolin-1(*2H*)-one (**4n**)

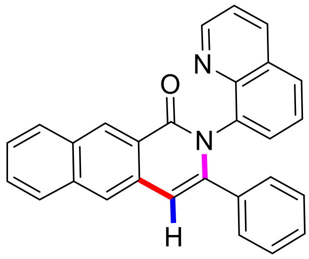

Rf 0.12 (hexane/EtOAc = 10/1). 68%, 27.1 mg. White solid; ^{1}H NMR ($CDCl_3$, 400 MHz) δ 9.08 (s, 1H), 8.91 (dd, J_1 = 1.6 Hz; J_2 = 4.0 Hz, 1H), 8.06-8.10 (m, 3H), 7.96 (d, *J* = 8.0 Hz, 1H), 7.72 (d, *J* = 8.0 Hz, 1H), 7.59 (t, *J* = 7.6 Hz, 1H), 7.48-7.57 (m, 2H), 7.35-7.44 (m, 2H), 7.12-7.15 (m, 2H), 7.03 (t, *J* = 7.6 Hz, 1H), 6.96 (t, *J* = 7.6 Hz, 2H), 6.76 (s, 1H). ^{13}C NMR ($CDCl_3$, 100 MHz) δ 163.8, 150.9, 144,9, 143.5, 137.4, 136.6, 136.1, 135.8, 133.3, 132.0, 130.9, 131.6, 129.9, 129.8, 128.8, 128.7, 128.1, 127.9, 127.6, 127.3, 125.9, 125.8, 124.2, 121.5, 107.6. HRMS (EI-TOF) calcd for $C_{28}H_{18}N_2O$ (M^+): 398.1419, found: 398.1413.

5-phenyl-6-(quinolin-8-yl)thieno[2,3-c]pyridin-7(*6H*)-one (**4o**)

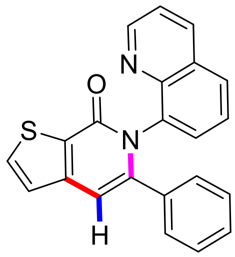

Rf 0.12 (hexane/EtOAc = 10/1). 92%, 32.4 mg. White solid; ^{1}H NMR ($CDCl_3$, 400 MHz) δ 8.91 (dd, J_1 = 1.6 Hz; J_2 = 4.0 Hz, 1H), 8.08 (dd, J_1 = 1.2 Hz; J_2 = 8.4 Hz, 1H), 7.75 (d, J = 5.2 Hz, 1H), 7.72 (dd, J_1 = 1.2 Hz; J_2 = 8.4 Hz, 1H), 7.50 (dd, J_1 = 1.2 Hz; J_2 = 7.2 Hz, 1H), 7.34-7.43 (m, 2H), 7.29 (d, J = 5.2 Hz, 1H), 7.08-7.10 (m, 2H), 7.00-7.02 (m, 1H), 6.94 (t, J = 7.2 Hz, 2H), 6.79 (s, 1H). ^{13}C NMR ($CDCl_3$, 100 MHz) δ 159.2, 151.0, 146.1, 145.4, 144.7, 136.9, 136.3, 136.1, 134.0, 130.9, 129.4, 128.9, 128.9, 128.8, 128.0, 127.3, 125.8, 124.6, 121.6, 104.5. HRMS (EI-TOF) calcd for $C_{22}H_{14}N_2OS$ (M^+): 354.0827, found: 354.0833.

5-phenyl-6-(quinolin-8-yl)furo[2,3-c]pyridin-7(*6H*)-one (**4p**)

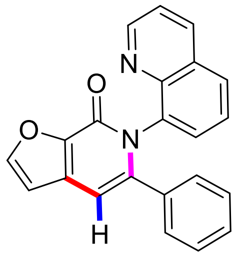

Rf 0.12 (hexane/EtOAc = 10/1). 45%, 15.2 mg. White solid; ^{1}H NMR ($CDCl_3$, 400 MHz) δ 8.91 (dd, J_1 = 1.6 Hz; J_2 = 4.4 Hz, 1H), 8.09 (dd, J_1 = 1.6 Hz; J_2 = 8.0 Hz, 1H), 7.82 (d, J = 2.0 Hz, 1H), 7.73 (dd, J_1 = 1.2 Hz; J_2 = 8.0 Hz, 1H), 7.50 (dd, J_1 = 1.2 Hz; J_2 = 7.2 Hz, 1H), 7.36-7.44 (m, 2H), 6.98-7.05 (m, 3H), 6.92 (t, J = 7.2 Hz, 2H), 6.77 (d, J = 2.0 Hz, 1H), 6.60 (s, 1H). ^{13}C NMR ($CDCl_3$, 100 MHz) δ 154.0, 151.0, 148.6, 145.5, 144.6, 143.0, 136.6, 136.3, 136.2, 133.1, 131.0, 129.0, 128.9, 128.8, 128.0, 127.2, 125.8, 121.6, 107.6, 101.7. HRMS (EI-TOF) calcd for $C_{22}H_{14}N_2O_2$ (M^+): 338.1005, found: 338.1008.

3-phenyl-2-(quinolin-8-yl)-5,6,7,8-tetrahydroisoquinolin-1(*2H*)-one (6a)

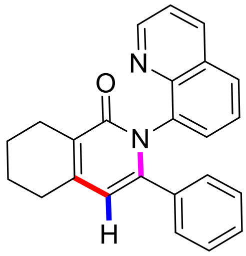

Rf 0.24 (hexane/EtOAc = 10/1). 53%, 18.7 mg. White solid; ^{1}H NMR ($CDCl_3$, 400 MHz) δ 8.94 (dd, J_1 = 1.2 Hz; J_2 = 4.0 Hz, 1H), 8.08 (dd, J_1 = 1.2 Hz; J_2 = 8.4 Hz, 1H), 7.69-7.73 (m, 1H), 7.35-7.44 (m, 3H), 6.98-7.04 (m, 3H), 6.92 (t, J = 7.6 Hz, 2H), 6.12 (s, 1H), 2.63-2.67 (m, 4H), 1.83 (s, 4H). ^{13}CNMR ($CDCl_3$, 100 MHz) δ 163.3, 151.0, 147.6, 145.7, 144.6, 137.2, 136.2, 136.0, 130.5, 128.8, 128.6, 128.6, 128.0, 127.3, 126.4, 125.8, 121.5, 109.9, 29.5, 23.9, 22.3, 22.1. HRMS (EI-TOF) calcd for $C_{24}H_{20}N_2O$ (M^+): 352.1576, found: 352.1577.

3,4-dimethyl-6-phenyl-1-(quinolin-8-yl)pyridin-2(*1H*)-one (6b)

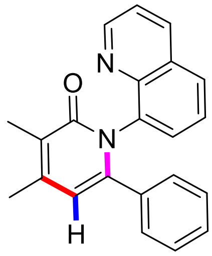

Rf 0.24 (hexane/EtOAc = 10/1). 43%, 14.0 mg. White solid; ^{1}H NMR ($CDCl_3$, 400 MHz) δ 8.94 (dd, J_1 = 1.6 Hz; J_2 = 4.0 Hz, 1H), 8.08 (dd, J_1 = 1.6 Hz; J_2 = 8.0 Hz, 1H), 7.71 (dd, J_1 = 2,0 Hz; J_2 = 7.6 Hz, 1H), 7.35-7.44 (m, 3H), 7.00-7.04 (m, 3H), 6.93 (t, J = 7.6 Hz, 2H), 6.20 (s, 1H), 2.30 (s, 3H), 2.20 (s, 3H). ^{13}C NMR ($CDCl_3$, 100 MHz) δ 163.5, 151.1, 146.4, 145.8, 144.6, 137.5, 136.1, 135.9, 130.3, 128.8, 128.6, 128.6, 128.0, 127.3, 125.8, 125.2, 121.5, 111.0, 20.1, 12.8. HRMS (EI-TOF) calcd for $C_{22}H_{18}N_2O$ (M^+): 326.1419, found: 326.1425.

4-ethyl-3-methyl-6-phenyl-1-(quinolin-8-yl)pyridin-2(*1H*)-one (6c)

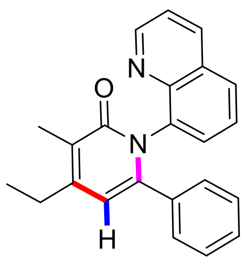

Rf 0.24 (hexane/EtOAc = 10/1). 71%, 24.1 mg. White solid; ^{1}H NMR ($CDCl_3$, 400 MHz) δ 8.93 (dd, J_1 = 2.0 Hz; J_2 = 4.4 Hz, 1H), 8.07 (dd, J_1 = 1.6 Hz; J_2 = 8.0 Hz, 1H), 7.69 (dd, J_1 = 1.6 Hz; J_2 = 8.0 Hz, 1H), 7.34-7.43 (m, 3H), 6.98-7.05 (m, 3H), 6.91-6.95 (m, 2H), 6.21 (s, 1H), 2.56-2.73 (m, 2H), 2.21 (s, 3H), 1.28 (t, J = 8.0 Hz, 3H). ^{13}C NMR ($CDCl_3$, 100 MHz) δ 163.8, 151.7, 151.0, 146.2, 144.6, 137.6, 136.1, 136.1, 130.3, 128.8, 128.6, 128.6, 128.0, 127.3, 125.8, 124.4, 121.5, 109.2, 26.8, 13.4, 12.3. HRMS (EI-TOF) calcd for $C_{23}H_{20}N_2O$ (M^+): 340.1576, found: 340.1576

3-methyl-4,6-diphenyl-1-(quinolin-8-yl)pyridin-2(*1H*)-one (6d)

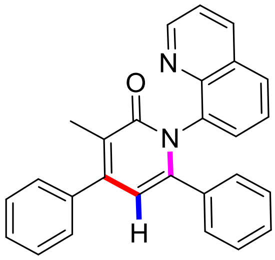

Rf 0.24 (hexane/EtOAc = 10/1). 42%, 16.3 mg. White solid; ^{1}H NMR ($CDCl_3$, 400 MHz) δ 9.00 (dd, J_1 = 1.6 Hz; J_2 = 4.0 Hz, 1H), 8.12 (dd, J_1 = 1.6 Hz; J_2 = 8.4 Hz, 1H), 7.74 (dd, J_1 = 1.2 Hz; J_2 = 8.0 Hz, 1H), 7.39-7.51 (m, 8H), 7.07-7.09 (m, 2H), 7.01 (d, J = 7.2 Hz, 1H), 6.92-6.96 (m, 2H), 6.34 (s, 1H), 2.21 (s, 3H). ^{13}C NMR ($CDCl_3$, 100 MHz) δ 164.1, 151.1, 149.5, 146.2, 144.6, 139.8, 137.5, 136.2, 135.9, 130.3, 128.8, 128.6, 128.6, 128.4, 128.1, 128.0, 127.4, 125.9, 125.1, 121.6, 110.1, 14.6. HRMS (EI-TOF) calcd for $C_{27}H_{20}N_2O$ (M^+): 388.1576, found: 388.1577.

3-(2-chlorophenyl)-6-phenyl-1-(quinolin-8-yl)pyridin-2(*1H*)-one (6e)

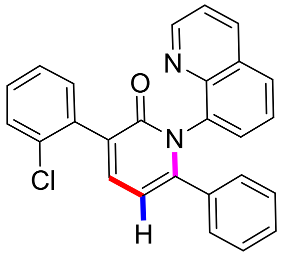

Rf 0.28 (hexane/EtOAc = 10/1). 81%, 33.0 mg. White solid; ^{1}H NMR ($CDCl_3$, 400 MHz) δ 8.93 (dd, J_1 = 1.6 Hz; J_2 = 7.6 Hz, 1H), 8.06 (dd, J_1 = 1.6 Hz; J_2 = 8.0 Hz, 1H), 7.67-7.72 (m, 2H), 7.50-7.57 (m, 2H), 7.35-7.45 (m, 3H), 7.21-7.27 (m, 2H), 7.11-7.13 (m, 2H), 7.05 (t, J = 7.2 Hz, 1H), 6.96-6.99 (m, 2H), 6.43 (d, J = 7.2 Hz, 1H). ^{13}C NMR ($CDCl_3$, 100 MHz) δ 161.8, 151.0, 150.6, 140.1, 137.0, 136.0, 135.8, 135.7, 133.7, 132.1, 130.3, 129.6, 129.0, 129.0, 128.9, 128.8, 128.8, 128.5, 128.4, 127.5, 126.4, 125.8, 121.6, 106.9. HRMS (EI-TOF) calcd for $C_{26}H_{17}ClN_2O$ (M^+): 408.1029, found: 408.1035.

3-(3-chlorophenyl)-6-phenyl-1-(quinolin-8-yl)pyridin-2(*1H*)-one (6f)

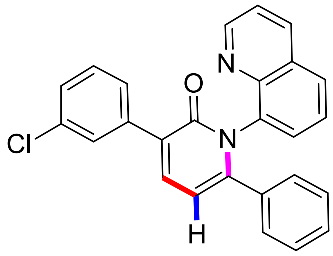

Rf 0.28 (hexane/EtOAc = 10/1). 79%, 32.2 mg. White solid; ^{1}H NMR ($CDCl_3$, 400 MHz) δ 8.92 (dd, J_1 = 1.6 Hz; J_2 = 4.0 Hz, 1H), 8.06 (dd, J_1 = 1.6 Hz; J_2 = 8.0 Hz, 1H), 7.66-7.71 (m, 2H), 7.50-7.56 (m, 2H), 7.34-7.45 (m, 3H), 7.21-7.29 (m, 2H), 7.11-7.13 (m, 2H), 7.04 (t, J = 7.2 Hz, 1H), 6.97 (t, J = 7.2 Hz, 2H), 6.43 (d, J = 6.8 Hz, 1H). ^{13}C NMR ($CDCl_3$, 100 MHz) δ 161.7, 150.9, 150.5, 144.2, 140.0, 136.9, 135.9, 135.6, 135.6, 133.6, 132.0, 130.2, 129.4, 128.9, 128.7, 128.7, 128.6, 128.4, 128.3, 127.3, 126.2, 125.6, 121.5, 106.7. HRMS (EI-TOF) calcd for $C_{26}H_{17}ClN_2O$ (M^+): 408.1029, found: 408.1022.

3-(4-chlorophenyl)-6-phenyl-1-(quinolin-8-yl)pyridin-2(*1H*)-one (6g)

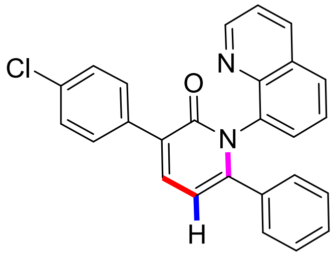

Rf 0.29 (hexane/EtOAc = 5/1). 86%, 35.1 mg. White solid; ^{1}H NMR ($CDCl_3$, 400 MHz) δ 8.94 (dd, J_1 = 1.6 Hz; J_2 = 4.4 Hz, 1H), 8.09 (dd, J_1 = 1.6 Hz; J_2 = 8.4 Hz, 1H), 7.67-7.79 (m, 4H), 7.37-7.49 (m, 4H), 6.95-7.10 (m, 6H), 6.45 (d, J = 7.2 Hz, 1H). ^{13}C NMR ($CDCl_3$, 100 MHz) δ 161.7, 151.2, 151.1, 144.2, 140.6, 140.5, 136.9, 136.2, 135.4, 130.1, 129.1, 129.0, 128.8, 128.6, 128.4, 127.5, 126.0, 125.8, 122.8, 121.8, 119.8, 107.2. HRMS (EI-TOF) calcd for $C_{26}H_{17}ClN_2O$ (M^+): 408.1029, found: 408.1029.

4,6-diphenyl-1-(quinolin-8-yl)pyridin-2(*1H*)-one (6h)

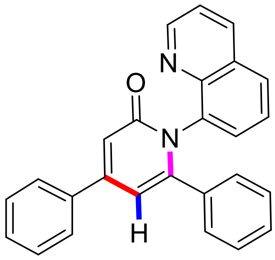

Rf 0.29 (hexane/EtOAc = 5/1). 65%, 24.3 mg. White solid; ^{1}H NMR ($CDCl_3$, 400 MHz) δ 8.96 (s, 1H), 8.09 (d, J = 8.0 Hz, 1H), 7.72 (d, J = 4.4 Hz, 3H), 7.38-7.50 (m, 6H), 6.94-7.11 (m, 6H), 6.60 (s, 1H). ^{13}C NMR ($CDCl_3$, 100 MHz) δ 151.8 151.1, 150.4, 148.4, 144.4, 137.8, 136.2, 135.9, 130.5, 129.5, 129.0, 129.0, 128.9, 128.5, 128.4, 127.4, 127.0, 125.9, 122.1, 121.7, 116.7, 107.5. HRMS (EI-TOF) calcd for $C_{26}H_{18}N_2O$ (M^+): 374.1419, found: 374.1420.

3,6-diphenyl-1-(quinolin-8-yl)pyridin-2(*1H*)-one (6i)

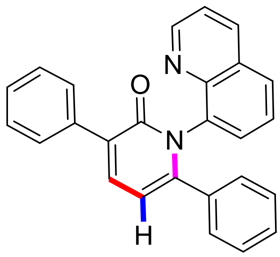

Rf 0.22 (hexane/EtOAc = 10/1). 55%, 20.6 mg. White solid; ^{1}H NMR ($CDCl_3$, 400 MHz) 8.94 (dd, J_1 = 1.6 Hz; J_2 = 4.4 Hz, 1H), 8.09 (dd, J_1 = 1.6 Hz; J_2 = 8.4 Hz, 1H), 7.82 (d, J = 7.2 Hz, 2H). 7.71-7.76 (m, 2H), 7.50 (dd, J_1 = 0.8 Hz; J_2 = 3.2 Hz, 1H), 7.26-7.44 (m, 5H), 7.03-7.10 (m, 3H), 6.96 (t, J = 7.2 Hz, 2H), 6.44 (d, J = 7.2 Hz, 1H). ^{13}C NMR ($CDCl_3$, 100 MHz) δ 162.4, 151.1, 149.7, 144.4, 137.9, 137.3, 136.9, 136.1, 135.8, 130.3, 129.0, 128.8, 128.5, 128.3, 128.1, 128.0, 127.5, 127.5, 126.0, 125.8, 121.6, 107.6. HRMS (EI-TOF) calcd for $C_{26}H_{18}N_2O$ (M^+): 374.1419, found: 374.1423.

3-methyl-6-phenyl-1-(quinolin-8-yl)pyridin-2(*1H*)-one (6j)

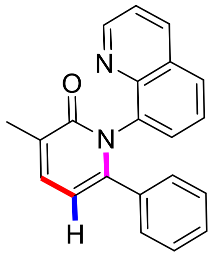

Rf 0.44 (hexane/EtOAc = 10/1). 77%, 24.0 mg. White solid; ^{1}H NMR ($CDCl_3$, 400 MHz) δ 8.94 (dd, J_1 = 1.6 Hz; J_2 = 4.0 Hz, 1H), 8.08 (d, J = 7.6 Hz, 1H), 7.71 (dd, J_1 = 1.6 Hz; J_2 = 8.0 Hz, 1H). 7.36-7.45 (m, 4H), 6.99-7.03 (m, 3H), 6.91-6.94 (m, 2H), 6.27 (d, J = 7.2 Hz, 1H), 2.25 (s, 3H). ^{13}C NMR ($CDCl_3$, 100 MHz) δ 163.8, 151.0, 147.9, 144.4, 137.3, 137.3, 136.2, 135.9, 131.0, 130.3, 128.8, 128.7, 128.6, 128.1, 127.4, 125.8, 121.6, 107.2, 17.3. HRMS (EI-TOF) calcd for $C_{21}H_{16}N_2O$ (M^+): 312,1263, found: 312.1267.

3-phenyl-2-(quinolin-8-yl)-4-(trimethylsilyl)isoquinolin-1(*2H*)-one (7a)

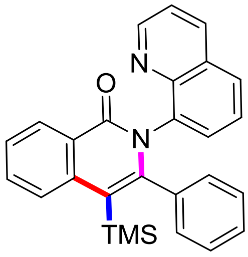

Rf 0.44 (hexane/EtOAc = 10/1). 76%, 31.9 mg. White solid; ^{1}H NMR ($CDCl_3$, 400 MHz) δ 8.93 (dd, J_1 = 1.6 Hz; J_2 = 4.0 Hz, 1H), 8.56 (dd, J_1 = 1.6 Hz; J_2 = 8.0 Hz, 1H),

8.07 (d, J = 7.6 Hz, 1H). 8.00 (d, J = 8.4 Hz, 1H), 7.69-7.74 (m, 1H), 7.64 (t, J = 4.8 Hz, 1H), 7.49-7.3 (m, 1H), 7.33-7.39 (m, 3H), 7.18 (d, J = 7.6 Hz, 1H), 7.04-7.08 (m, 1H), 6.95 (t, J = 7.6 Hz, 1H), 6.87 (d, J = 7.6 Hz, 1H), 6.63 (t, J = 7.2 Hz, 1H), 0.00 (s, 9H). ^{13}C NMR ($CDCl_3$, 100 MHz) δ 160.9, 148.4, 147.2, 138.5, 135.5, 135.2, 134.2, 129.6, 129.2, 129.1, 127.9, 126.7, 126.5, 126.3, 126.1, 125.5, 124.8, 124.5, 124.0, 123.7, 119.3, 109.5, 0.01. HRMS (EI-TOF) calcd for$C_{27}H_{24}N_2OSi$ (M^+): 420.1658, found: 420.1654.

3-(4-bromophenyl)-2-(quinolin-8-yl)-4-(trimethylsilyl)isoquinolin-1(*2H*)-one (7b)

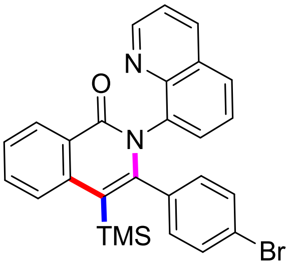

Rf 0.20 (hexane/EtOAc = 10/1). 81%, 40.3 mg. White solid; ^{1}H NMR ($CDCl_3$, 400 MHz) δ 8.88 (dd, J_1 = 1.6 Hz; J_2 = 4.0 Hz, 1H), 8.54 (dd, J_1 = 1.2 Hz; J_2 = 8.0 Hz, 1H), 8.06 (dd, J_1 = 1.6 Hz; J_2 = 8.4 Hz, 1H). 7.98 (d, J = 8.0 Hz, 1H), 7.66-7.71 (m, 2H), 7.51 (t, J = 7.2 Hz, 1H), 7.34-7.37 (m, 3H), 7.20 (d, J = 8.4 Hz, 1H), 7.05 (d, J = 8.0 Hz, 1H), 6.77 (s, 2H), 0.02 (s, 9H). ^{13}C NMR ($CDCl_3$, 100 MHz) δ 160.6, 148.5, 145.7, 142.4, 138.1, 135.2, 134.0, 133.9, 130.6, 129.5, 129.4, 128.7, 127.9, 127.5, 126.6, 126.5, 125.3, 124.1, 123.5, 120.2, 119.3, 109.6, 0.01. HRMS (EI-TOF) calcd for $C_{27}H_{23}BrN_2OSi$ (M^+): 498.0763, found: 498.0763.

3-(3-chlorophenyl)-2-(quinolin-8-yl)-4-(trimethylsilyl)isoquinolin-1(*2H*)-one (7c)
4-(3-chlorophenyl)-2-(quinolin-8-yl)-3-(trimethylsilyl)isoquinolin-1(2H)-one (7c′)

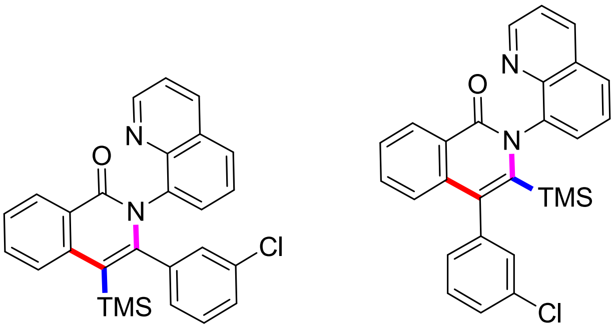

The ratio of two isomers was 1:1 as determined by ^{1}H NMR. Rf 0.20 (hexane/EtOAc

= 10/1). 90%, 40.9 mg. White solid; ^{1}H NMR (CDCl$_3$, 400 MHz, a mixture of two isomer) δ 8.93 (dd, J_1 = 1.6 Hz; J_2 = 4.0 Hz, 1H), 8.88 (dd, J_1 = 1.6 Hz; J_2 = 4.0 Hz, 1H), 8.54-8.57 (m, 2H), 8.04-8.08 (m, 2H), 8.00 (d, J = 8.4 Hz, 2H), 7.70-7.74 (m, 2H), 7.65-7.67 (m, 2H), 7.50-7.54 (m, 2H), 7.34-7.40 (m, 6H), 7.19 (t, J = 1.6 Hz, 1H), 7.03 (d, J = 7.6 Hz, 1H), 6.90-6.97 (m, 4H), 6.77 (d, J = 8.0 Hz, 1H), 6.56 (t, J = 8.0 Hz, 1H), 0.03 (s, 18H). ^{13}C NMR (CDCl$_3$, 100 MHz) δ 160.7, 160.6, 148.6, 148.6, 145.5, 145.4, 142.5, 142.4, 138.1, 136.7, 136.5, 135.2, 133.9, 130.6, 130.4, 129.6, 129.1, 128.9, 128.7, 128.3, 127.1, 126.6, 126.6, 126.5, 126.5, 126.5, 126.1, 126.1, 126.0, 125.8, 125.7, 125.4, 124.2, 124.0, 123.9, 123.6, 123.5, 119.4, 119.3, 109.7, 109.5, 0.003. HRMS (EI-TOF) calcd for $C_{27}H_{23}ClN_2OSi$ (M$^+$): 454.1268, found: 454.1267.

3-(pyridin-2-yl)-2-(quinolin-8-yl)-4-(trimethylsilyl)isoquinolin-1(*2H*)-one (7d)

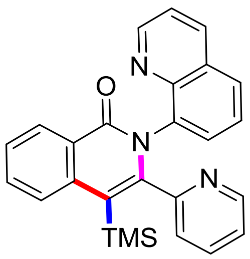

Rf 0.20 (hexane/EtOAc = 5/1). 83%. 34.9 mg. White solid; ^{1}H NMR (CDCl$_3$, 400 MHz) δ 8.89 (dd, J_1 = 1.6 Hz; J_2 = 4.0 Hz, 1H), 8.57 (dd, J_1 = 1.2 Hz; J_2 = 8.0 Hz, 1H), 8.32 (d, J = 4.0 Hz, 1H), 8.03 (dd, J_1 = 2.8 Hz; J_2 = 8.4 Hz, 2H). 7.70-7.74 (m, 1H), 7.61-7.63 (m, 1H), 7.52 (t, J = 7.2 Hz, 2H), 7.31-7.36 (m, 2H), 7.02 (d, J = 6.8 Hz, 2H), 6.84-6.88 (m, 1H), 0.00 (s, 9H). ^{13}C NMR (CDCl$_3$, 100 MHz) δ 161.1, 153.7, 149.0, 146.4, 145.9, 142.9, 138.7, 135.5, 134.3, 132.9, 130.0, 130.0, 127.1, 126.8, 126.0, 124.7, 124.6, 124.3, 124.0, 121.2, 119.7, 109.9, 0.01. HRMS (EI-TOF) calcd for $C_{26}H_{23}N_3OSi$ (M$^+$): 421.1610, found: 421.1605.

3-(prop-1-en-2-yl)-2-(quinolin-8-yl)-4-(trimethylsilyl)isoquinolin-1(*2H*)-one (7e)
4-(prop-1-en-2-yl)-2-(quinolin-8-yl)-3-(trimethylsilyl)isoquinolin-1(*2H*)-one (7e′)

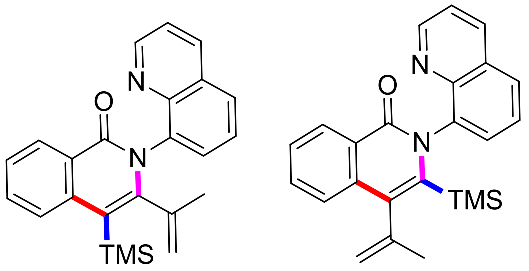

The ratio of two isomers was 1:1 as determined by ^{1}H NMR. Rf 0.20 (hexane/EtOAc = 5/1). 57%. 21.9 mg. White solid; ^{1}H NMR ($CDCl_3$, 400 MHz, a mixture of two isomer) δ 8.93 (dd, J_1 = 1.6 Hz; J_2 = 4.4 Hz, 1H), 8.83 (dd, J_1 = 1.6 Hz; J_2 = 4.4 Hz, 1H), 8.47-8.50 (m, 2H), 8.15-8.22 (m, 2H), 7.88-7.97 (m, 4H), 7.55-7.72 (m, 5H), 7.35-7.47 (m, 5H), 5.20 (s, 1H), 5.07 (s, 1H), 4.84 (s, 1H), 4.54 (s, 1H), 1.74 (s, 3H), 1.15 (s, 3H), 0.43 (d, J = 3.2 Hz, 18H). ^{13}C NMR ($CDCl_3$, 100 MHz) δ 163.4, 162.9, 151.4, 151.1, 151.1, 150.7, 145.4, 145.1, 142.2, 140.8, 140.8, 139.5, 137.6, 137.3, 136.3, 136.0, 131.9, 131.5, 130.3, 129.2, 129.1, 128.8, 128.8, 128.7, 128.7, 127.5, 127.4, 126.0, 125.9, 125.8, 125.8, 125.7, 123.0, 121.9, 121.6, 121.5, 109.9, 108.7, 26.0, 23.3, 3.32, 3.31. HRMS (EI-TOF) calcd for $C_{24}H_{24}N_2OSi$ (M^+): 384.1658, found: 384.1655.

7-iodo-3-phenyl-2-(quinolin-8-yl)-4-(trimethylsilyl)isoquinolin-1(*2H*)-one (7f)

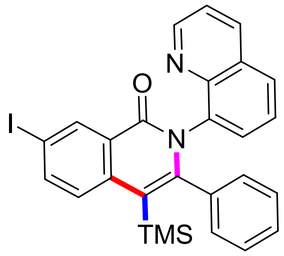

Rf 0.20 (hexane/EtOAc = 5/1). 37%. 20.2 mg. White solid; ^{1}H NMR ($CDCl_3$, 400 MHz) δ 8.87-8.90 (m, 2H), 8.04 (dd, J_1 = 1.6 Hz; J_2 = 4.0 Hz, 1H), 7.97 (dd, J_1 = 2.0 Hz; J_2 = 8.8 Hz, 1H), 7.72 (d, J = 8.4 Hz, 1H), 7.64 (dd, J_1 = 4.4 Hz; J_2 = 6.0 Hz, 1H), 7.32-7.38 (m, 3H), 7.15 (d, J = 7.6 Hz, 1H), 7.04-7.07 (m, 1H), 6.93-6.96 (m, 1H), 6.81 (d, J = 8.0 Hz, 1H), 6.60-6.64 (m, 1H), -0.03 (s, 9H). ^{13}C NMR ($CDCl_3$, 100 MHz) δ 159.6, 148.7, 148.1, 142.6, 138.3, 137.8, 135.6, 135.0, 134.0, 129.1, 128.9, 127.9, 127.3, 126.6, 126.4, 125.8, 125.0, 124.7, 123.7, 119.5, 109.2, 88.9, 0.01. HRMS (EI-TOF) calcd for $C_{27}H_{23}IN_2OSi$ (M^+): 546.0624, found: 546.0622.

7-chloro-3-phenyl-2-(quinolin-8-yl)-4-(trimethylsilyl)isoquinolin-1(*2H*)-one (7g)

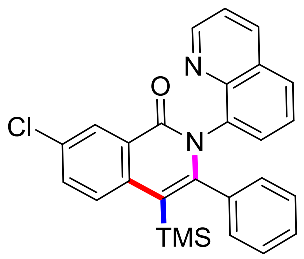

Rf 0.20 (hexane/EtOAc = 5/1). 36%. 16.3 mg. White solid; ^{1}H NMR ($CDCl_3$, 400 MHz) δ 8.90 (dd, J_1 = 1.6 Hz; J_2 = 4.0 Hz, 1H), 8.50 (d, J = 2.4 Hz, 1H), 8.03-8.06 (m, 1H), 7.94 (d, J = 8.8 Hz, 1H), 7.63-7.66 (m, 2H), 7.32-7.38 (m, 3H), 7.16 (d, J = 7.6 Hz, 1H), 7.04-7.08 (m, 1H), 6.93-6.97 (m, 1H), 6.82 (d, J = 7.6 Hz, 1H), 6.60-6.64 (m, 1H), -0.02 (s, 9H). ^{13}C NMR ($CDCl_3$, 100 MHz) δ 160.0, 148.7, 147.7, 142.6, 137.0, 135.4, 135.0, 134.0, 130.0, 129.2, 128.9, 128.0, 127.1, 126.6, 126.4, 126.1, 125.4, 124.9, 124.6, 123.7, 119.5, 109.1, 0.006. HRMS (EI-TOF) calcd for $C_{27}H_{23}ClN_2OSi$ (M^+): 454.1268, found: 454.1276.

6-phenyl-5-(quinolin-8-yl)-7-(trimethylsilyl)thieno[3,2-c]pyridin-4(*5H*)-one (7h)

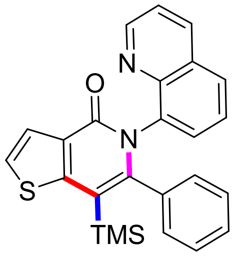

Rf 0.20 (hexane/EtOAc = 5/1). 76%. 32.4 mg. White solid; ^{1}H NMR ($CDCl_3$, 400 MHz) δ 8.91 (dd, J_1 = 1.6 Hz; J_2 = 4.0 Hz, 1H), 8.03 (dd, J_1 = 1.6 Hz; J_2 = 8.8 Hz, 1H), 7.78 (d, J = 5.2 Hz, 1H), 7.63 (dd, J_1 = 1.6 Hz; J_2 = 8.0 Hz, 1H), 7.57 (d, J = 5.2 Hz, 1H), 7.31-7.38 (m, 3H), 7.15 (d, J = 7.6 Hz, 1H), 7.03-7.07 (m, 1H), 6.94 (t, J = 7.6 Hz, 1H), 6.85 (d, J = 7.6 Hz, 1H), 6.61-6.65 (m, 1H), -0.03 (s, 9H). ^{13}C NMR ($CDCl_3$, 100 MHz) δ 157.6, 149.4, 148.8, 147.5, 143.4, 135.9, 135.6, 134.7, 131.5, 129.8, 129.8, 128.5, 127.3, 126.9, 125.6, 125.3, 125.0, 124.3, 120.2, 109.2, 0.017. HRMS (EI-TOF) calcd for $C_{25}H_{22}N_2OSSi$ (M^+): 426.1222, found: 426.1222

3-methyl-6-phenyl-1-(quinolin-8-yl)-5-(trimethylsilyl)pyridin-2(*1H*)-one (7i)

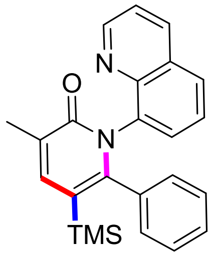

Rf 0.20 (hexane/EtOAc = 5/1). 52%. 20.0 mg. White solid; ^{1}H NMR ($CDCl_3$, 400 MHz) δ 8.92-8.93 (m, 1H), 8.02-8.04 (m, 1H), 7.62 (dd, J_1 = 1.6 Hz; J_2 = 8.0 Hz, 1H), 7.53 (s, 1H), 7.32-7.38 (m, 3H), 7.05-7.13 (m, 2H), 6.96 (t, J = 7.6 Hz, 1H), 6.82 (d, J = 8.0 Hz, 1H), 6.68 (t, J = 7.6 Hz, 1H), 2.26 (s, 3H), -0.11 (s, 9H). ^{13}C NMR ($CDCl_3$, 100 MHz) δ 163.3, 152.5, 150.7, 144.2, 142.2, 137.5, 136.1, 135.9, 130.3, 129.2, 128.4, 128.1, 127.6, 126.8, 126.6, 125.6, 121.4, 112.9, 17.1, 0.015. HRMS (EI-TOF) calcd for $C_{24}H_{24}N_2OSi$ (M^+): 384.1658, found: 384.1655.

3-methyl-4,6-diphenyl-1-(quinolin-8-yl)-5-(trimethylsilyl)pyridin-2(*1H*)-one (7j)

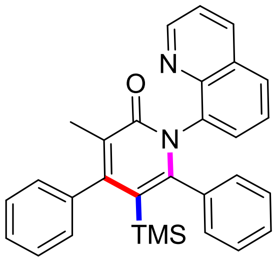

Rf 0.20 (hexane/EtOAc = 5/1). 52%. 23.9 mg. White solid; ^{1}H NMR ($CDCl_3$, 400 MHz) δ 9.00-9.01 (m, 1H), 8.06 (dd, J_1 = 1.2 Hz; J_2 = 8.4 Hz, 1H), 7.63 (dd, J_1 = 4.0 Hz; J_2 = 5.6 Hz, 1H), 7.37-7.47 (m, 5H), 7.32-7.33 (m, 3H), 7.20 (d, J = 8.0 Hz, 1H), 7.07 (t, J = 7.6 Hz, 1H), 6.95 (t, J = 7.2 Hz, 1H), 6.86 (d, J = 8.0 Hz, 1H), 6.62 (t, J = 7.6 Hz, 1H), 1.94 (s, 3H), -0.59 (s, 9H). ^{13}C NMR ($CDCl_3$, 100 MHz) δ 160.6, 153.2, 149.8, 148.4, 142.2, 139.1, 135.6, 134.6, 133.6, 128.6, 128.0, 127.6, 127.2, 126.5, 125.9, 125.6, 125.2, 124.4, 124.2, 123.3, 119.0, 111.6, 12.2, 0.014. HRMS (EI-TOF) calcd for $C_{30}H_{28}N_2OSi$ (M^+): 460.1971, found: 460.1971.

Gram-Scale Synthesis of 3a and 7a

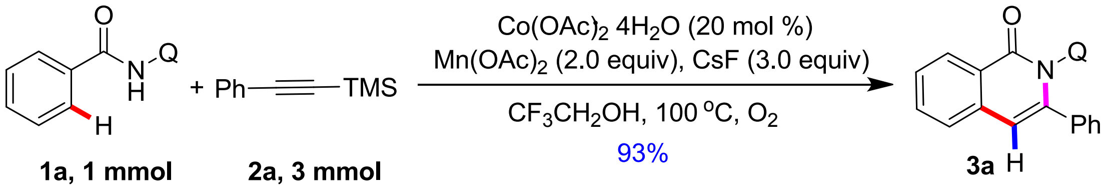

A 25 mL sealed tube was charged with amide **1a** (1 mmol), alkynylsilanes **2a** (3 mmol), $Co(OAc)_2 \cdot 4H_2O$ (50 mg, 0.2 mmol), $Mn(OAc)_2$ (346 mg, 2 mmol), CsF (456 mg, 3 mmol) and CF_3CH_2OH (10 mL). The vial was evacuated and filled with O_2 atmosphere for five times, and stirred at 100 °C for 24 h. The mixture was then cooled to room temperature, diluted with EtOAc, filtered through a celite pad, and concentrated in vacuo. The residue was purified by flash column chromatography on silica gel, eluting with EtOAc/hexane (1:5 ~ 1:1, v/v), to afford the desired product **3a** (yield = 93%).

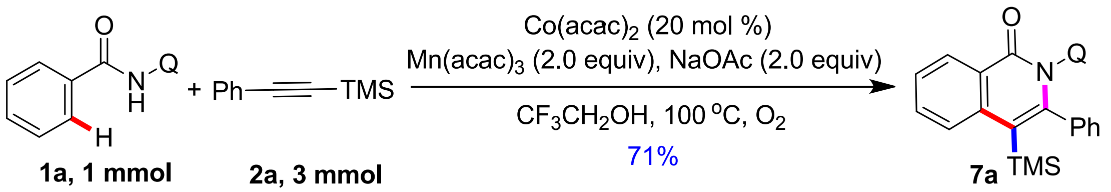

A 25 mL sealed tube was charged with amide **1a** (1 mmol), alkynylsilanes **2a** (3 mmol), $Co(acac)_2$ (52 mg, 0.2 mmol), $Mn(acac)_3$ (704 mg, 2 mmol), NaOAc (164 mg, 2 mmol) and CF_3CH_2OH (10 mL). The vial was evacuated and filled with O_2 atmosphere for five times, and stirred at 100 °C for 24 h. The mixture was then cooled to room temperature, diluted with EtOAc, filtered through a celite pad, and concentrated in vacuo. The residue was purified by flash column chromatography on silica gel, eluting with EtOAc/hexane (1:5 ~ 1:2, v/v), to afford the desired product **7a** (yield = 71%).

The H/D Exchange Experiments

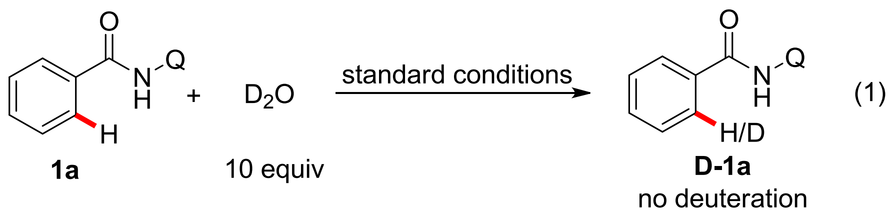

A 25 mL sealed tube was charged with amide **1a** (0.1 mmol), D_2O (20 mg, 1 mmol), $Co(OAc)_2 \cdot 4H_2O$ (5 mg, 0.02 mmol), $Mn(OAc)_2$ (34.6 mg, 0.2 mmol), CsF (45.6 mg, 0.3 mmol) and CF_3CH_2OH (1.0 mL). The vial was evacuated and filled with O_2 atmosphere for five times, and stirred at 100 °C for 24 h. The mixture was then cooled to room temperature, diluted with EtOAc, filtered through a celite pad, and concentrated in vacuo. The residue was purified by flash column chromatography on silica gel, eluting with EtOAc/hexane (1:5, v/v), to afford the desired product **D-1a**.

2-methyl-*N*-(quinolin-8-yl)benzamide 1a

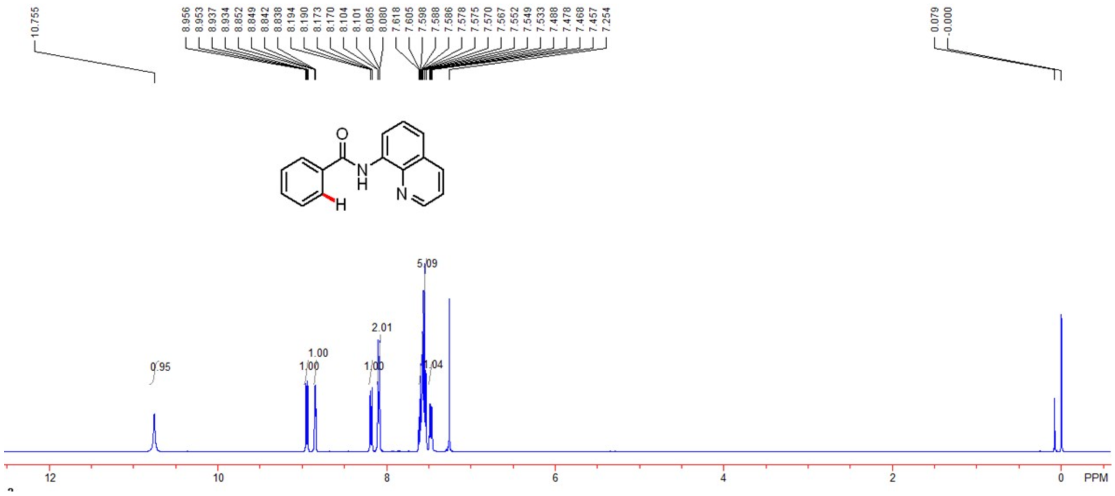

D-1a

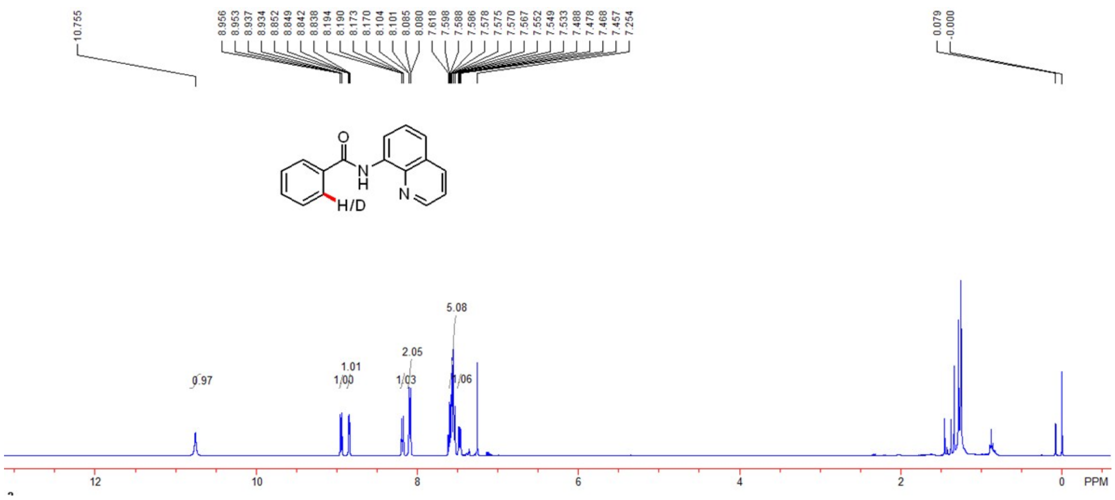

The Kinetic Isotope Experiments

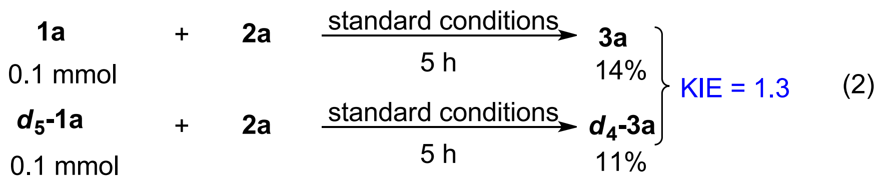

A 25 mL sealed tube was charged with amide **1a** (0.1 mmol), alkynylsilanes **2a** (0.3 mmol), $Co(OAc)_2 \cdot 4H_2O$ (5 mg, 0.02 mmol), $Mn(OAc)_2$ (34.6 mg, 0.2 mmol), CsF (45.6 mg, 0.3 mmol) and CF_3CH_2OH (1.0 mL). The vial was evacuated and filled with O_2 atmosphere for five times, and stirred at 100 °C for 5 h. The mixture was then cooled to room temperature, diluted with EtOAc, filtered through a celite pad, and concentrated in vacuo. The residue was purified by flash column chromatography on silica gel, eluting with EtOAc/hexane (1:5 ~ 1:1, v/v), to afford the desired product **3a** (yield = 14%).

A 25 mL sealed tube was charged with amide ***d*$_5$-1a** (0.1 mmol), alkynylsilanes **2a** (0.3 mmol), $Co(OAc)_2 \cdot 4H_2O$ (5 mg, 0.02 mmol), $Mn(OAc)_2$ (34.6 mg, 0.2 mmol), CsF (45.6 mg, 0.3 mmol) and CF_3CH_2OH (1.0 mL). The vial was evacuated and filled with O_2 atmosphere for five times, and stirred at 100 °C for 5 h. The mixture was then cooled to room temperature, diluted with EtOAc, filtered through a celite pad, and concentrated in vacuo. The residue was purified by flash column chromatography on silica gel, eluting with EtOAc/hexane (1:5 ~ 1:1, v/v), to afford the desired product

***d_4*-3a** (yield = 11%).

***d_5*-1a**

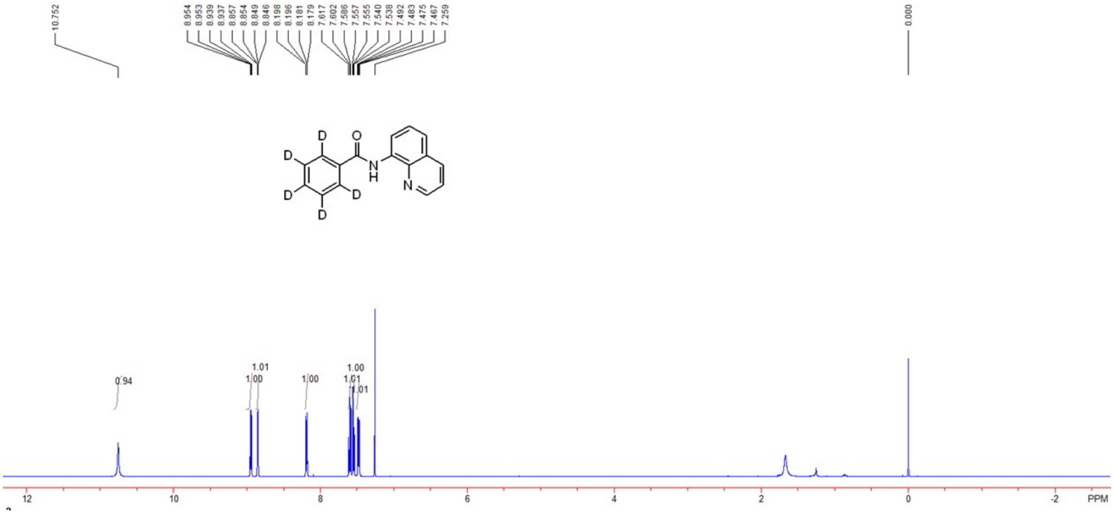

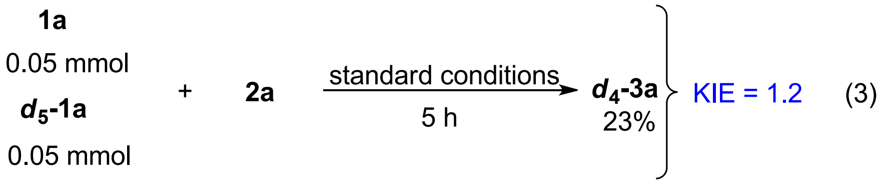

A 25 mL sealed tube was charged with amide **1a** (0.05 mmol), amide ***d_5*-1a** (0.05 mmol), alkynylsilanes **2a** (0.3 mmol), $Co(OAc)_2{\cdot}4H_2O$ (5 mg, 0.02 mmol), $Mn(OAc)_2$ (34.6 mg, 0.2 mmol), CsF (45.6 mg, 0.3 mmol) and CF_3CH_2OH (1.0 mL). The vial was evacuated and filled with O_2 atmosphere for five times, and stirred at 100 °C for 5 h. The mixture was then cooled to room temperature, diluted with EtOAc, filtered through a celite pad, and concentrated in vacuo. The residue was purified by flash column chromatography on silica gel, eluting with EtOAc/hexane (1:5 ~ 1:1, v/v), to afford the desired product **3a** and ***d_4*-3a** (yield = 23%).

***d_4*-3d**

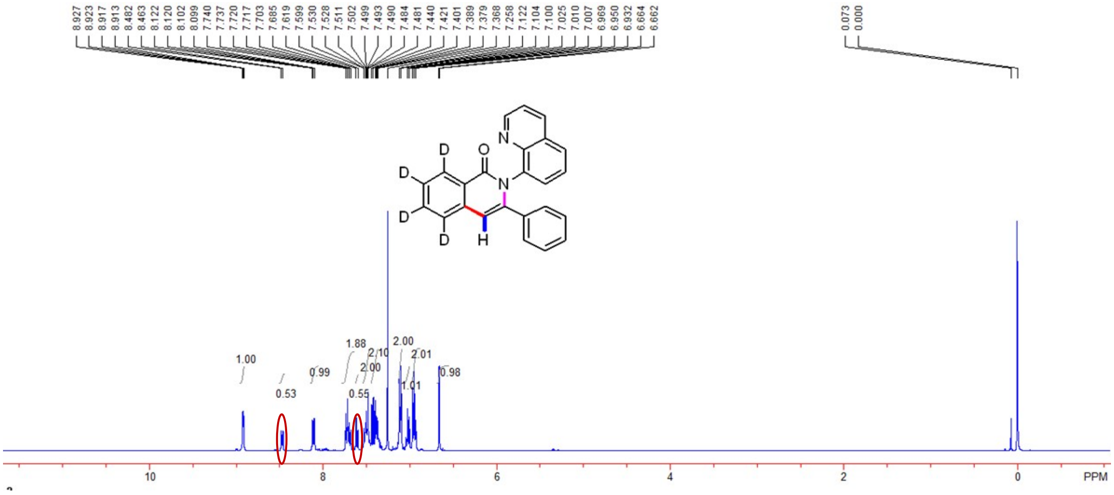

References

1. L. D.Tran, I. Popov and O. Daugulis, *J. Am. Chem. Soc.,* 2012, **134**, 18237.
2. L. Grigorjeva and O. Daugulis, *Angew. Chem., Int. Ed.,* 2014, **53**, 10209
3. Y. Ano, M. Tobisu and N. Chatani, *Org. Lett.,* 2012, **14**, 354.
4. X. Cong, Y. Li, Y. Wei and X. Zeng, *Org. Lett.,* 2014, **16**, 3926.

Copies of ^{1}H and ^{13}C NMR Spectra

3-phenyl-2-(quinolin-8-yl)isoquinolin-1(*2H*)-one (3a)

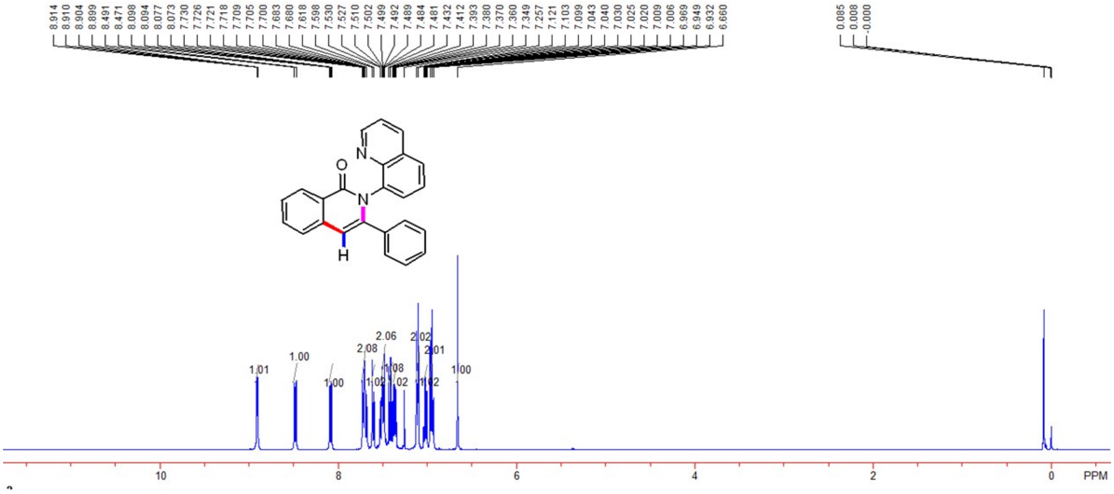

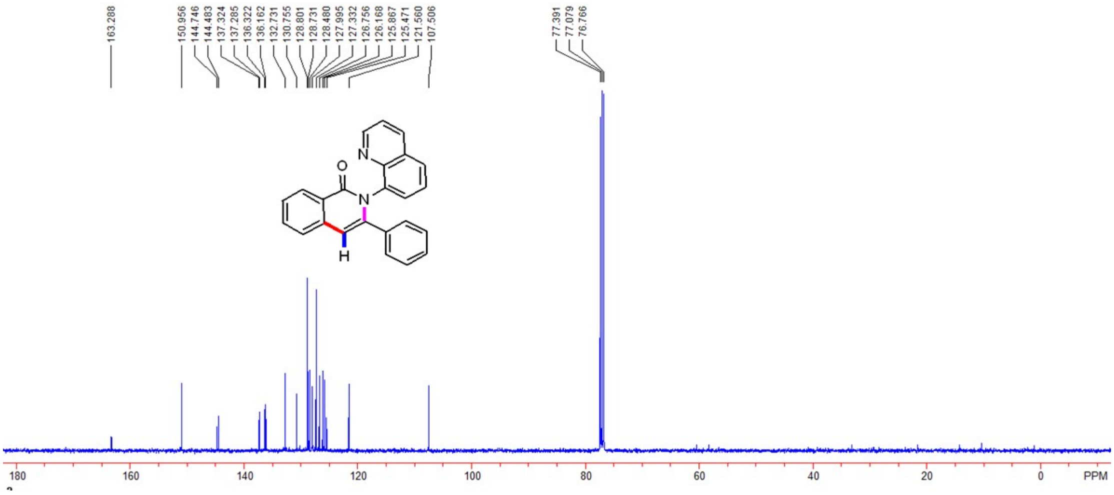

3-(4-bromophenyl)-2-(quinolin-8-yl)isoquinolin-1(*2H*)-one (3b)

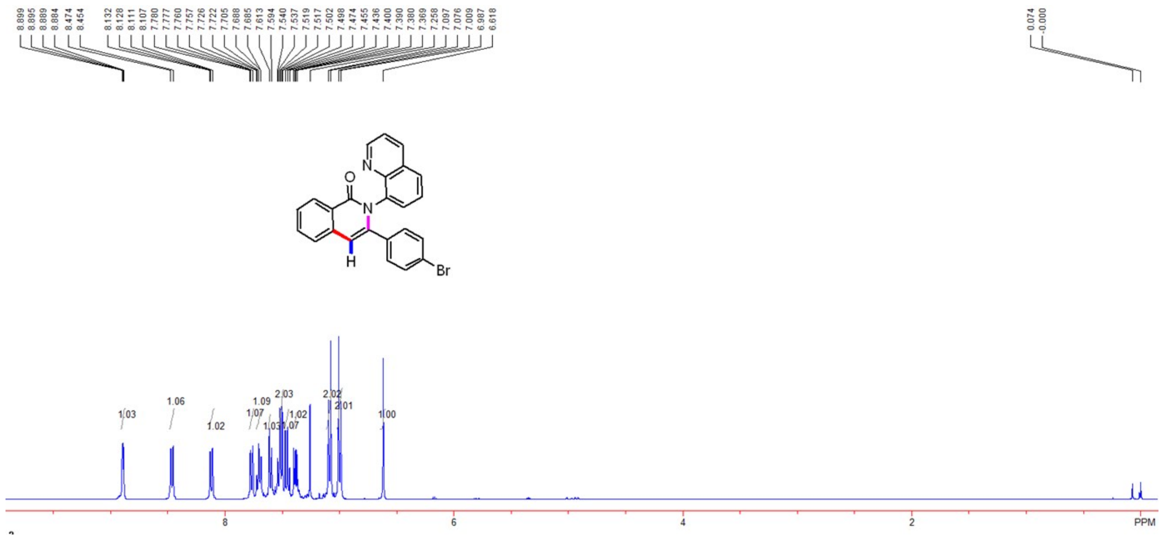

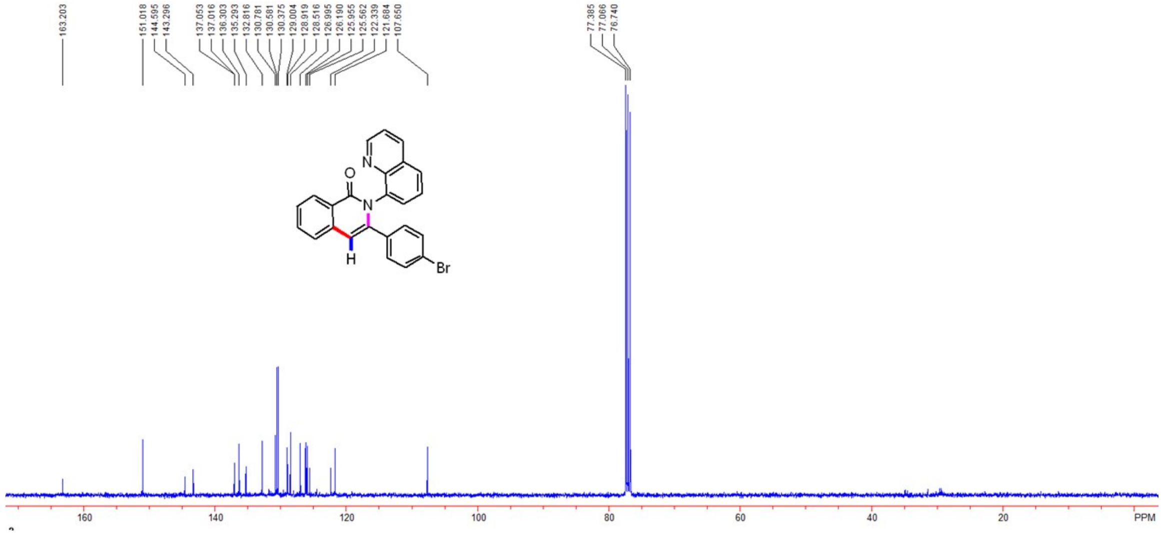

3-(pyridin-2-yl)-2-(quinolin-8-yl)isoquinolin-1(*2H*)-one (3c)

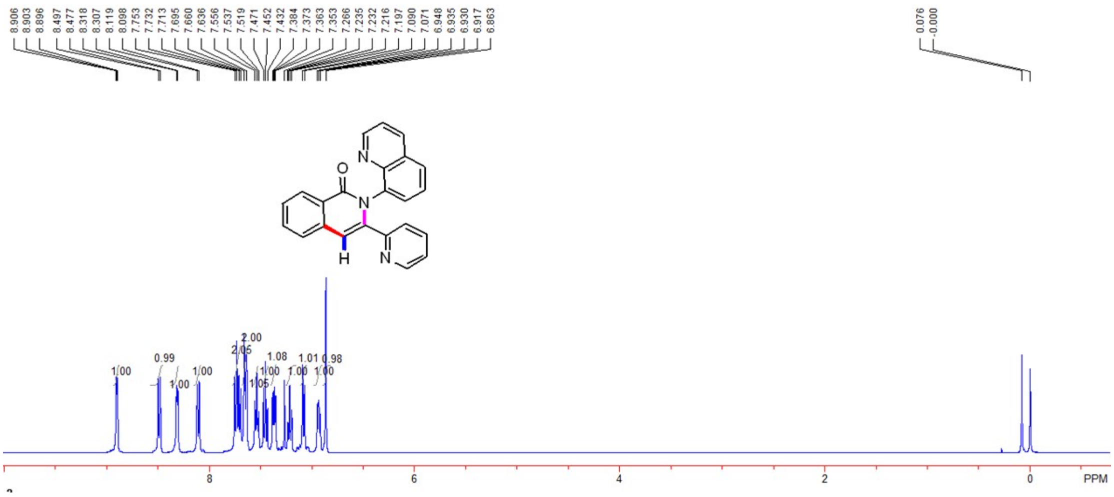

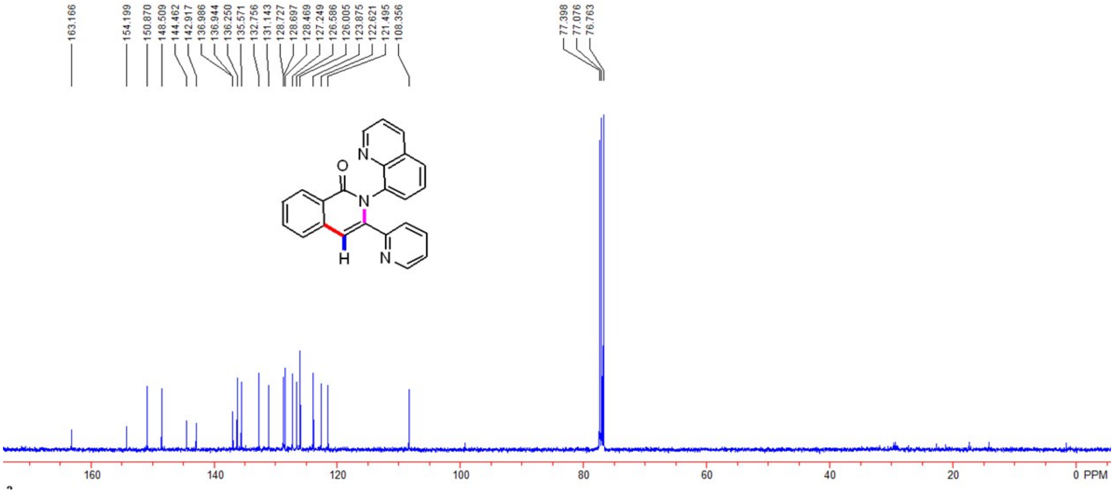

3-cyclohexyl-2-(quinolin-8-yl)isoquinolin-1(*2H*)-one (3d)

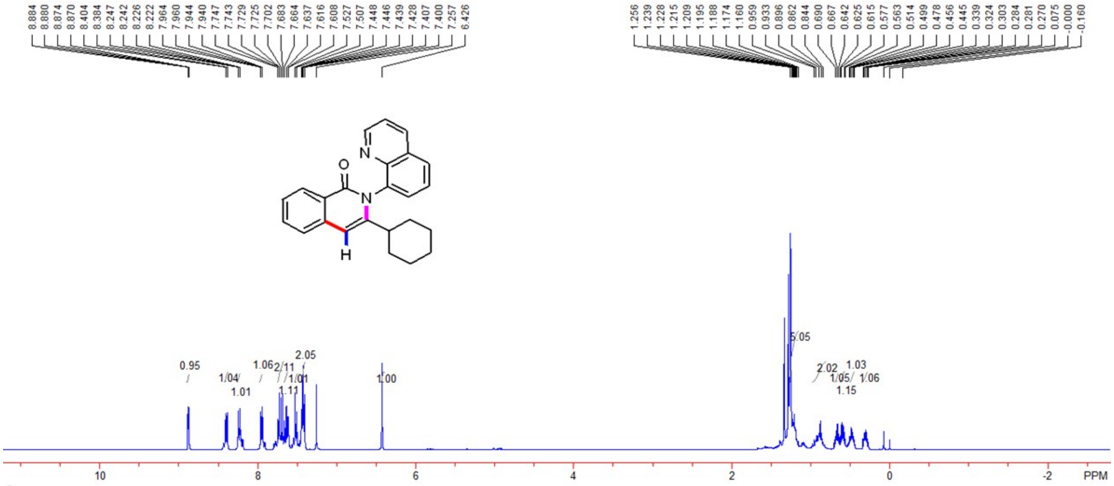

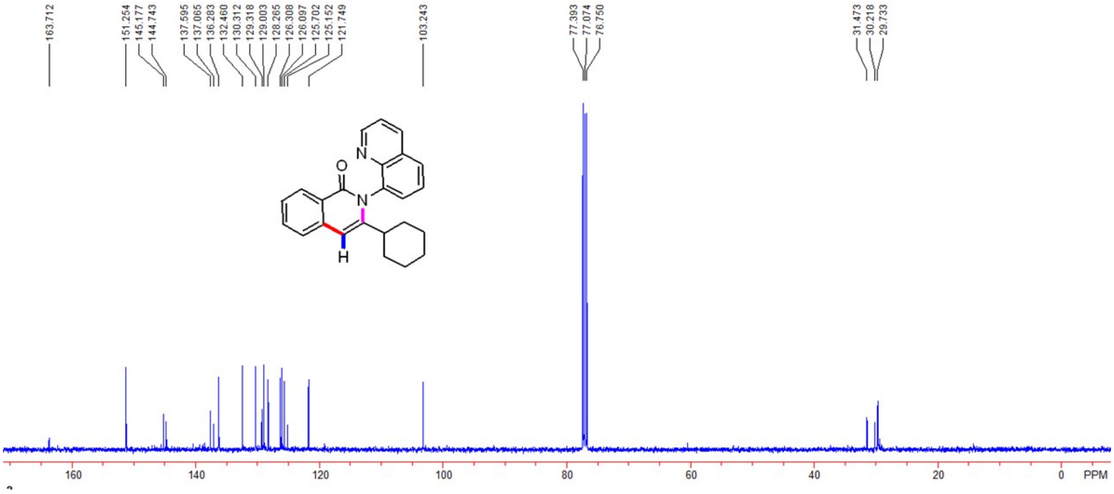

3-methyl-2-(quinolin-8-yl)isoquinolin-1(*2H*)-one (3e)

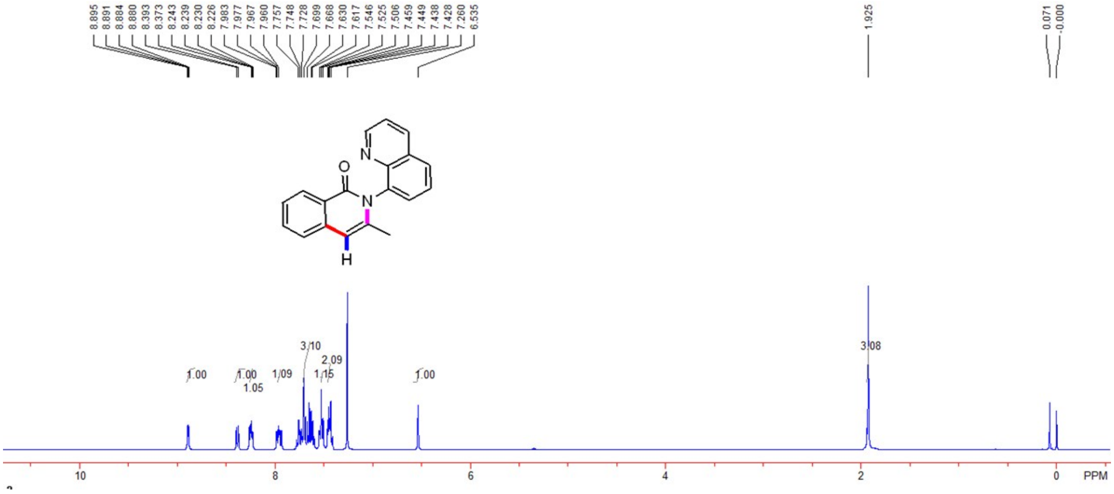

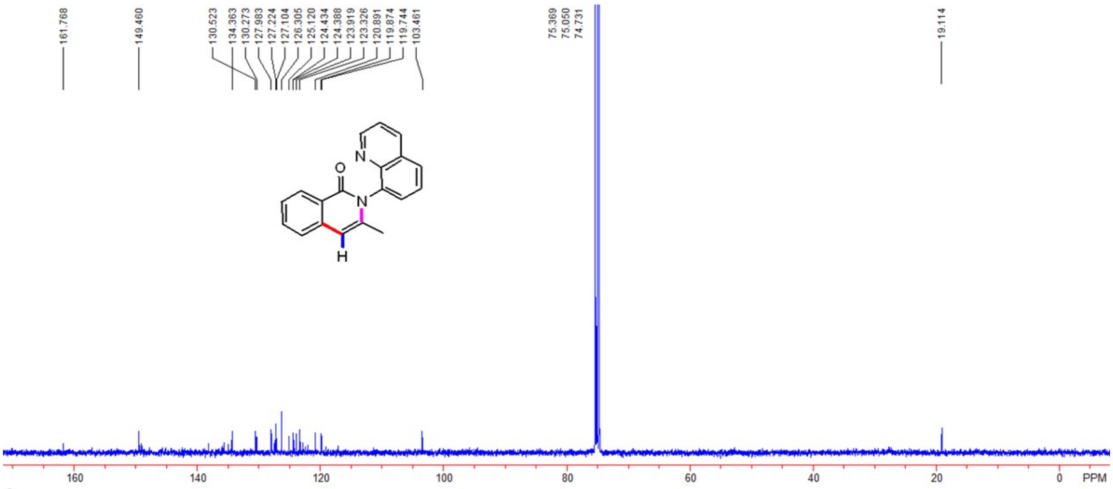

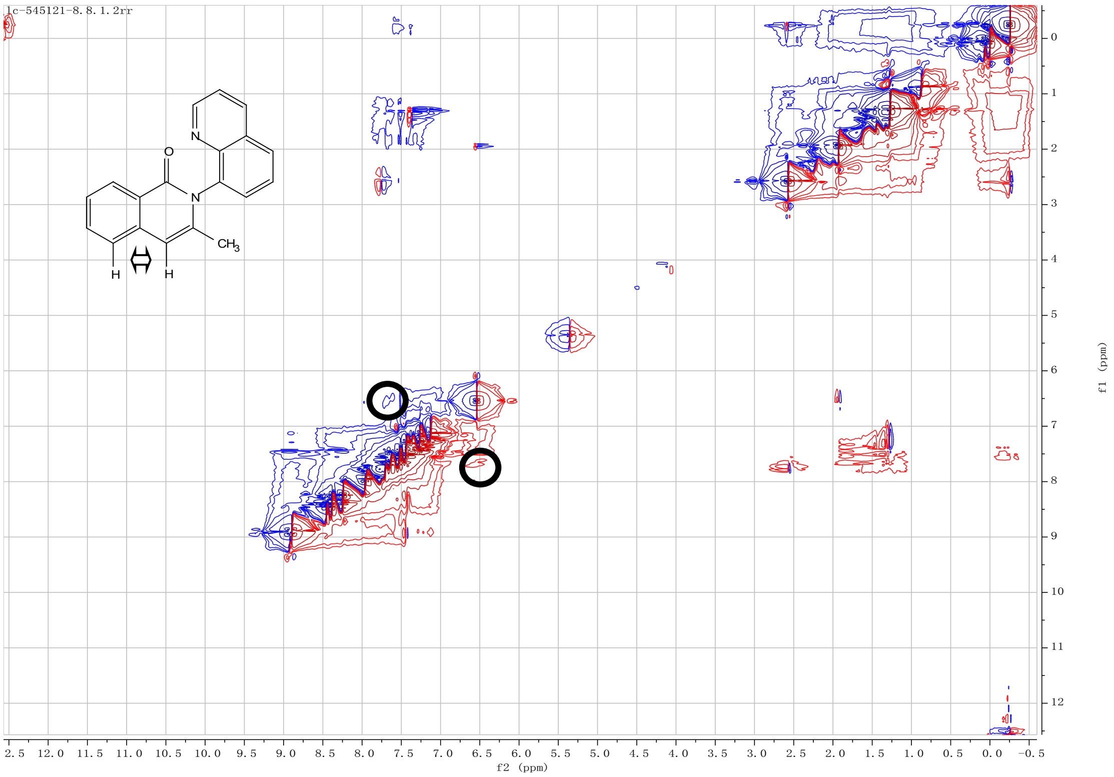

2-(quinolin-8-yl)-3-(trimethylsilyl)isoquinolin-1(*2H*)-one (3f)

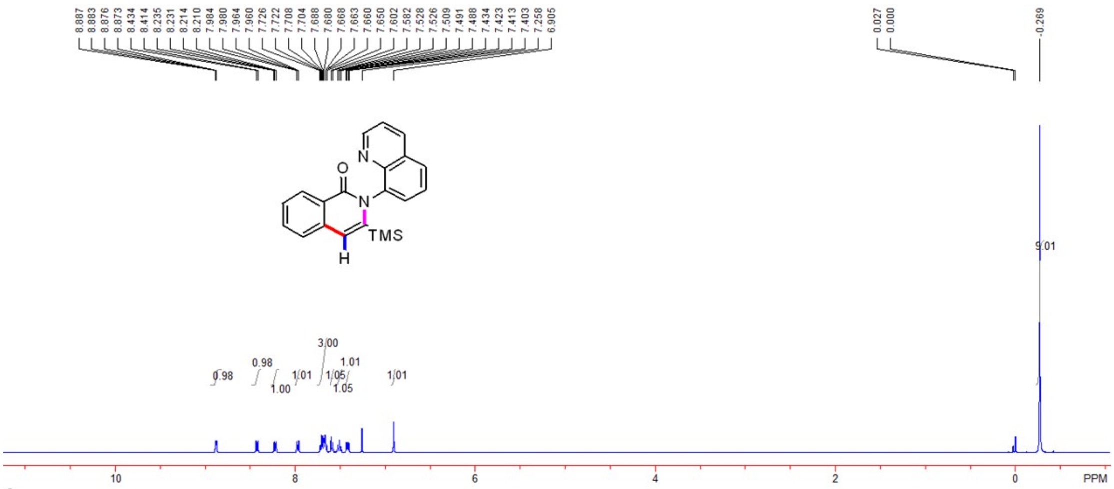

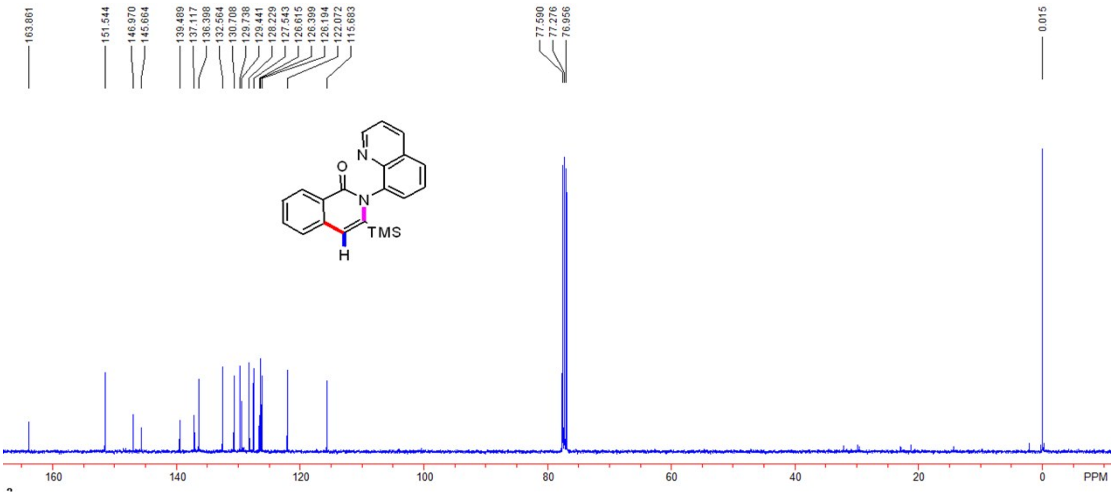

8-methyl-3-phenyl-2-(quinolin-8-yl)isoquinolin-1(*2H*)-one (4a)

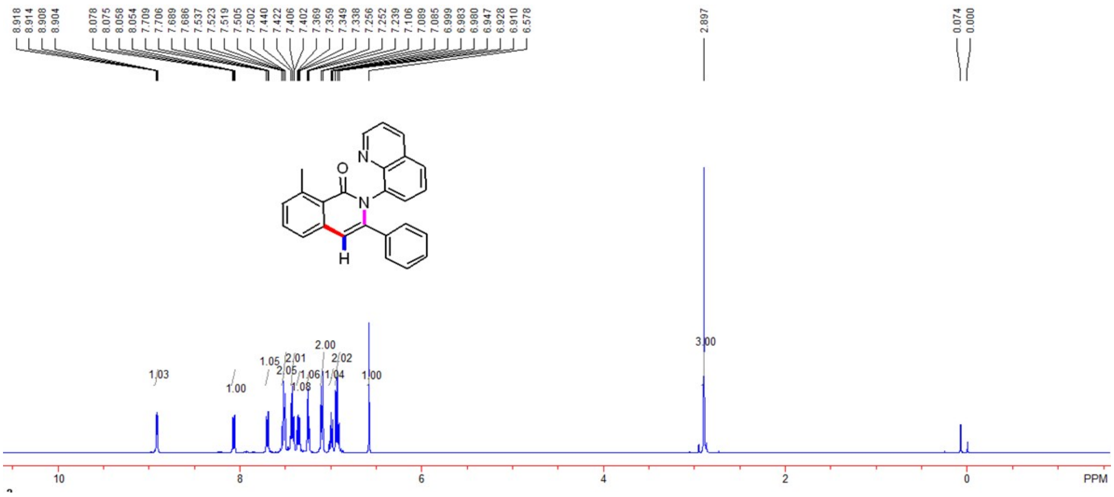

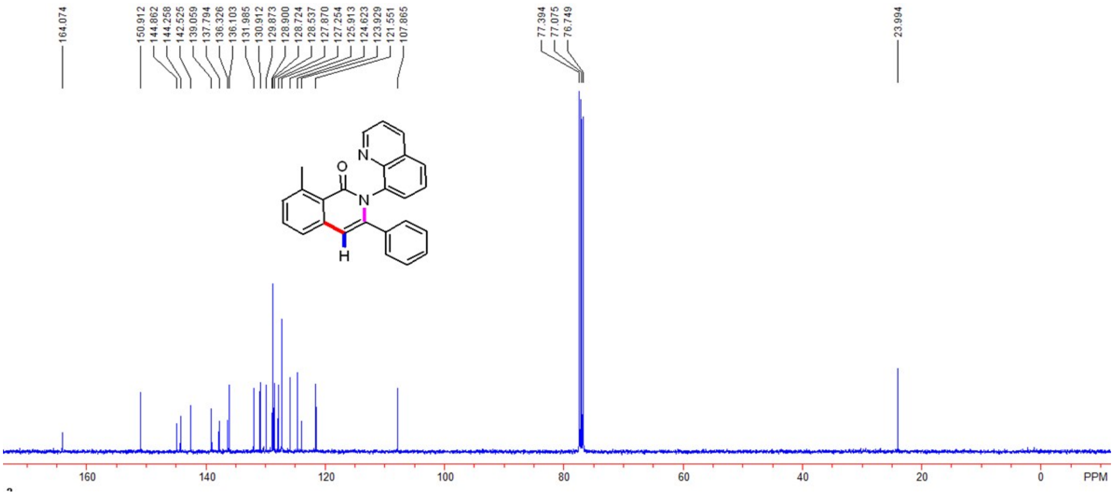

7-methyl-3-phenyl-2-(quinolin-8-yl)isoquinolin-1(*2H*)-one (4b)

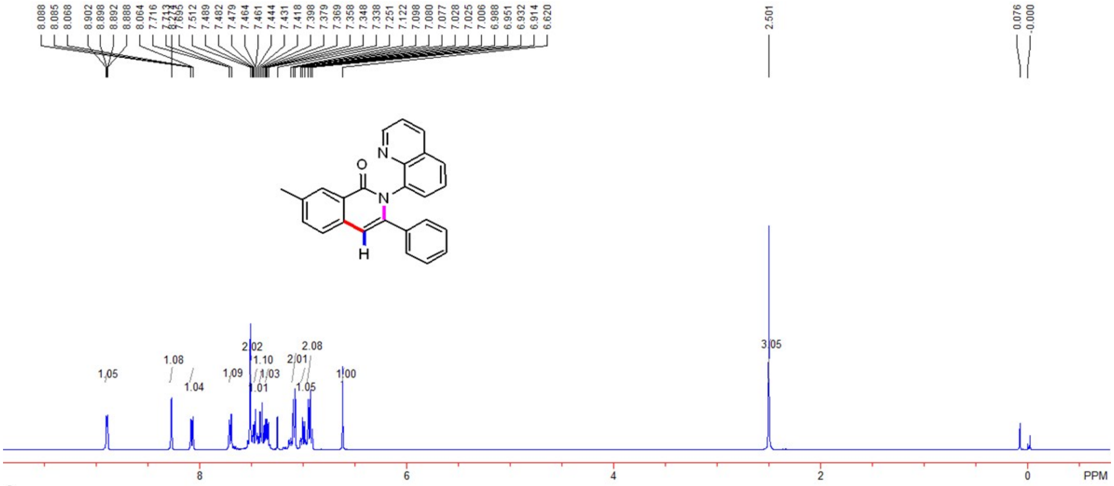

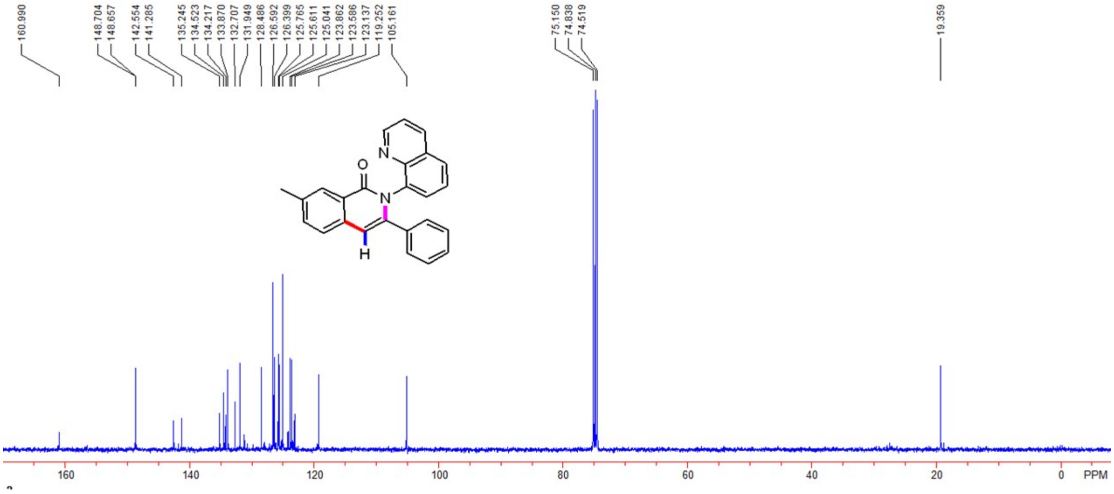

6-methyl-3-phenyl-2-(quinolin-8-yl)isoquinolin-1(*2H*)-one (4c)

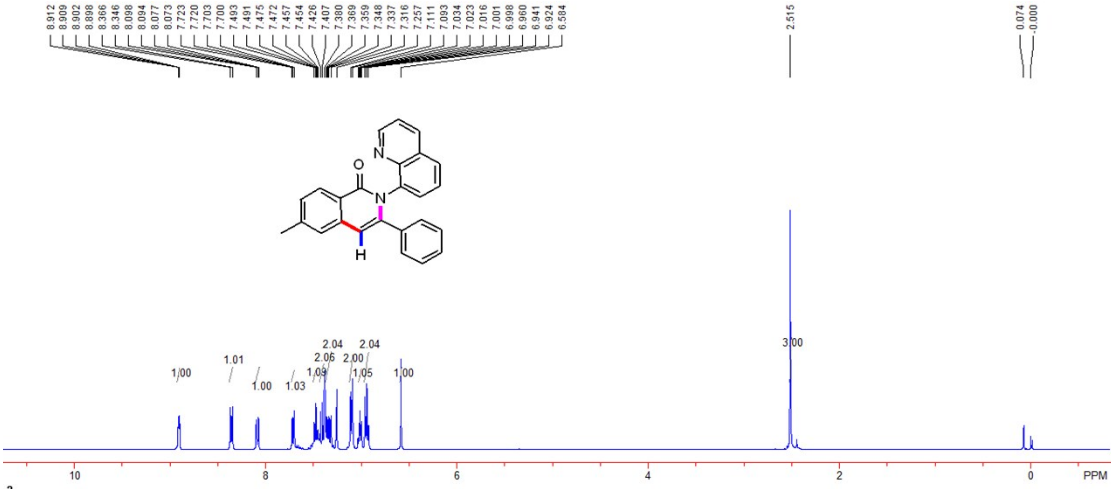

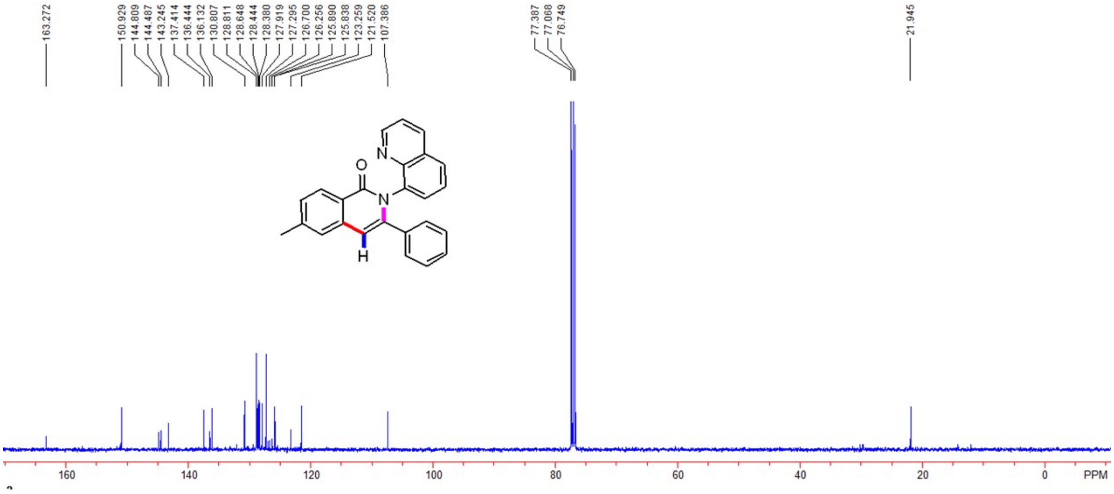

6-methoxy-3-phenyl-2-(quinolin-8-yl)isoquinolin-1(*2H*)-one (4d)

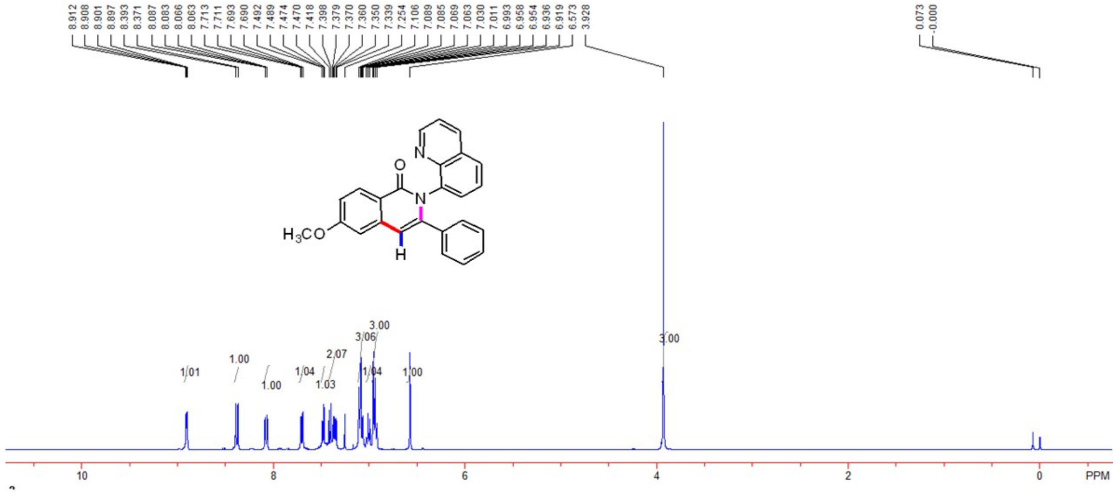

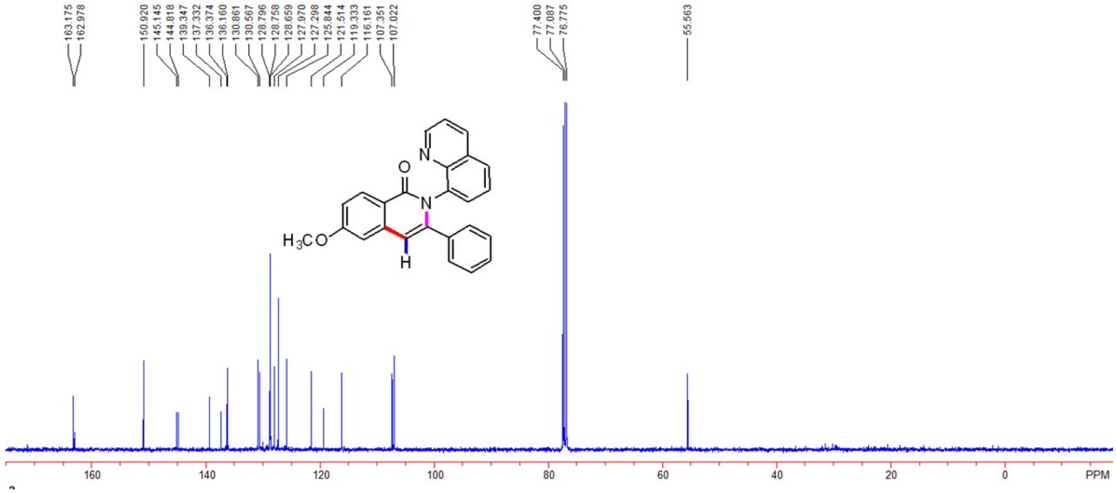

6-(tert-butyl)-3-phenyl-2-(quinolin-8-yl)isoquinolin-1(*2H*)-one (4e)

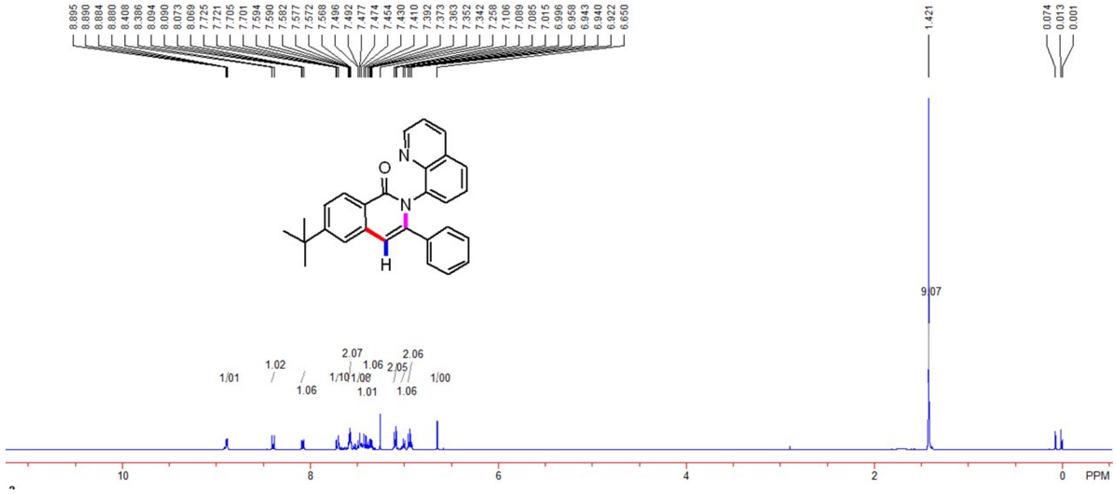

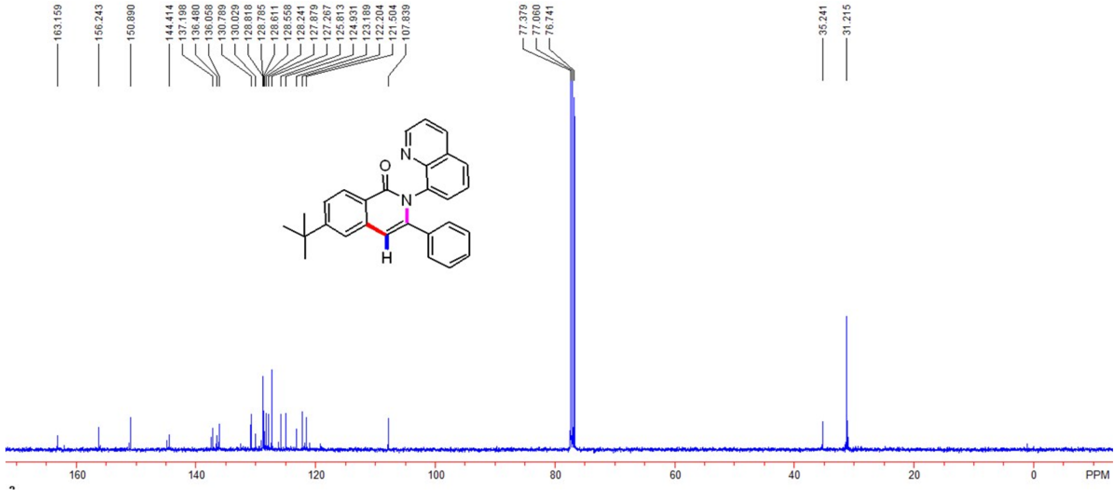

6,7-dimethoxy-3-phenyl-2-(quinolin-8-yl)isoquinolin-1(*2H*)-one (4f)

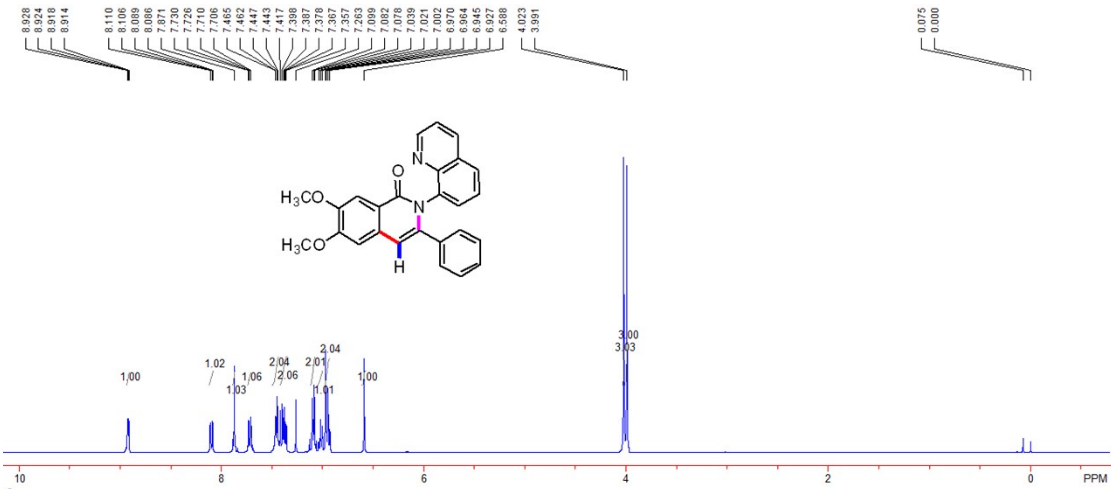

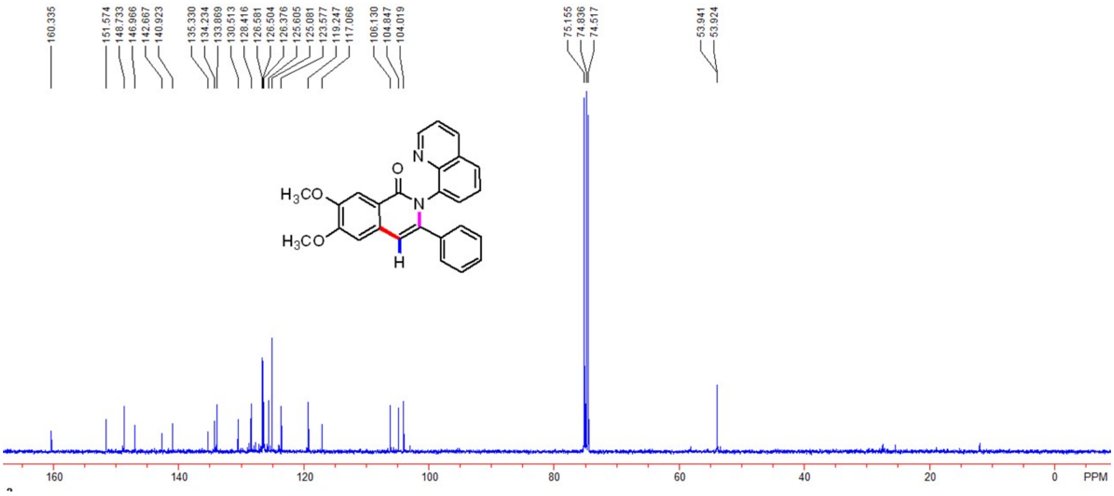

7-iodo-3-phenyl-2-(quinolin-8-yl)isoquinolin-1(*2H*)-one (4g)

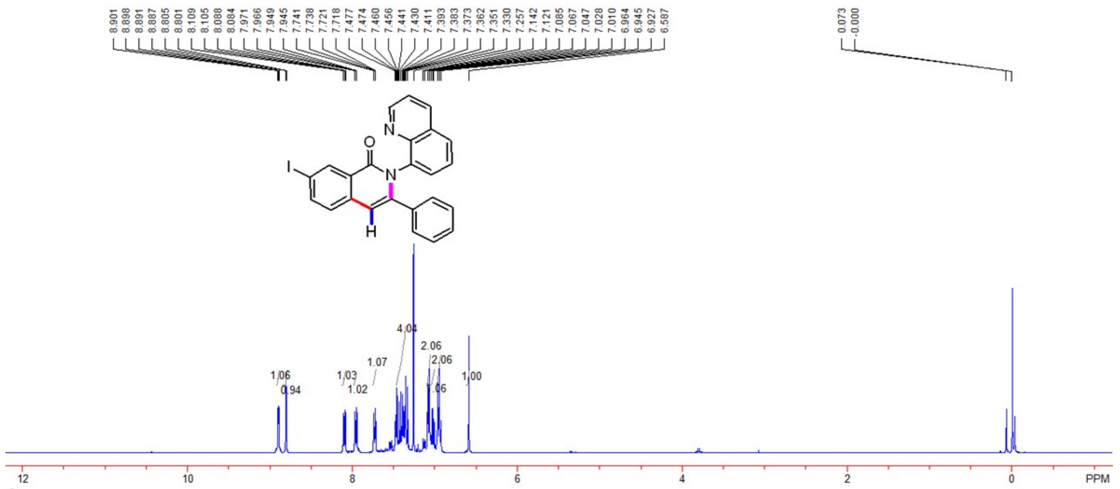

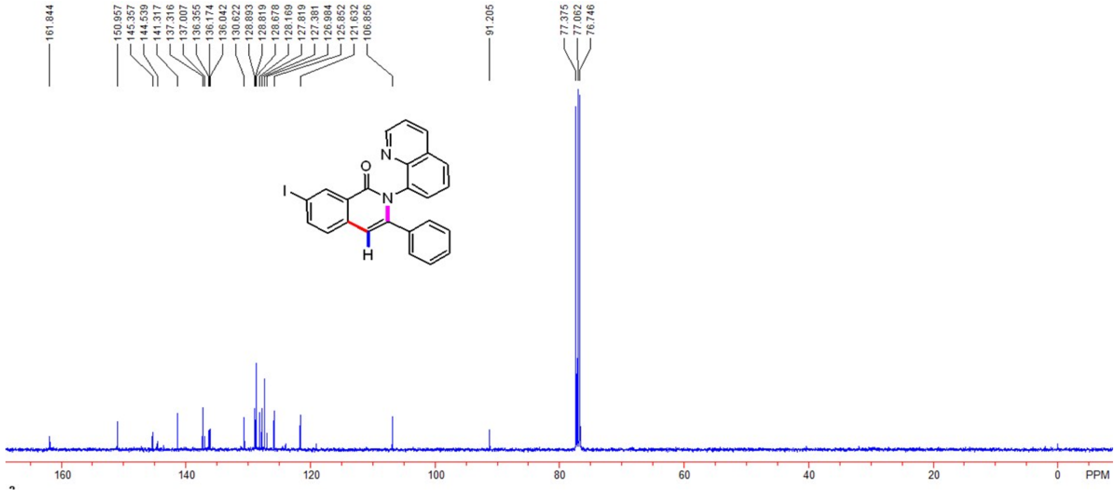

7-bromo-3-phenyl-2-(quinolin-8-yl)isoquinolin-1(*2H*)-one (4h)

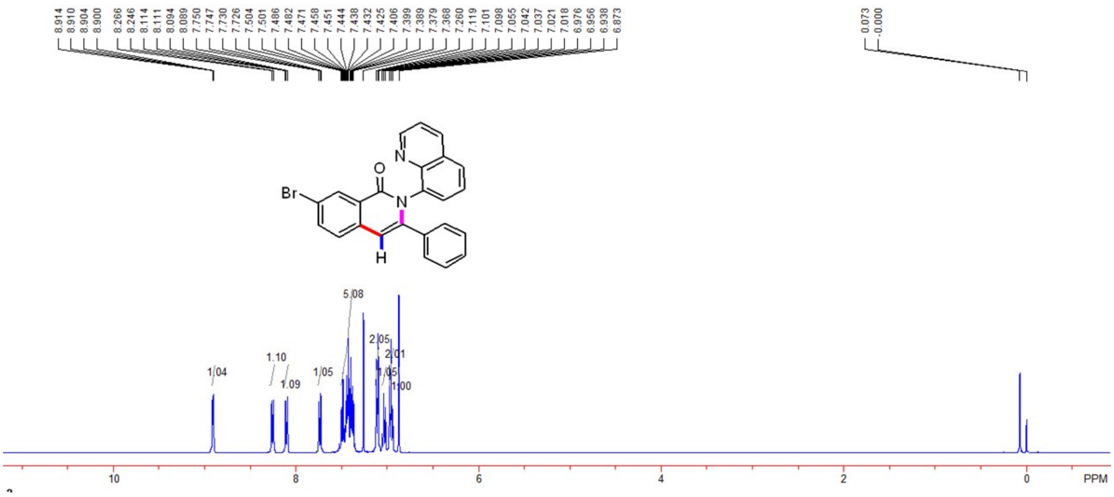

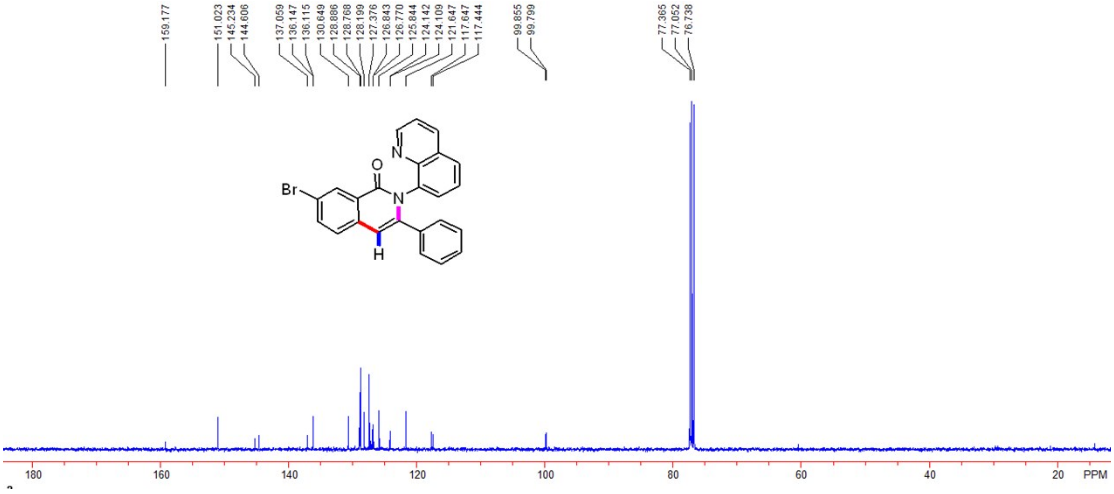

6-chloro-3-phenyl-2-(quinolin-8-yl)isoquinolin-1(*2H*)-one (4i)

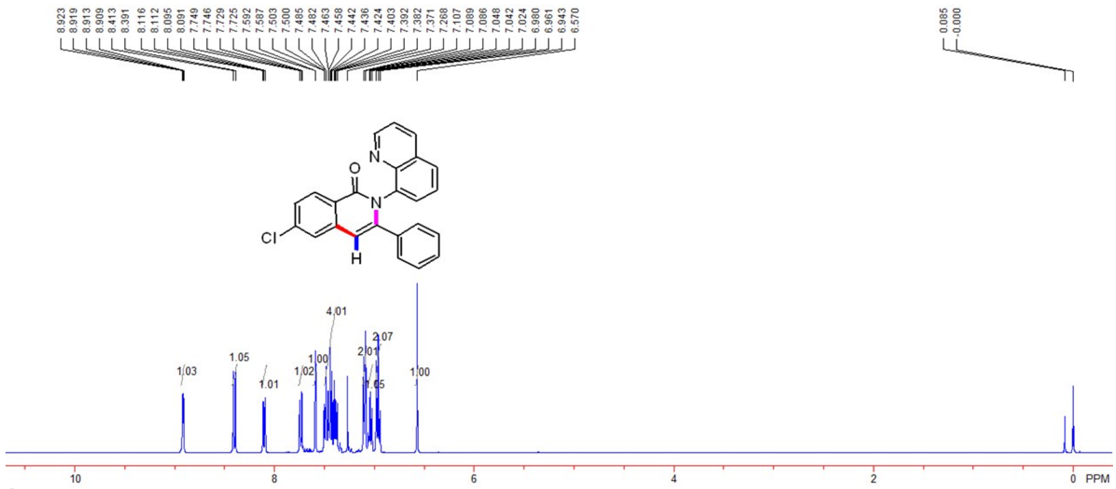

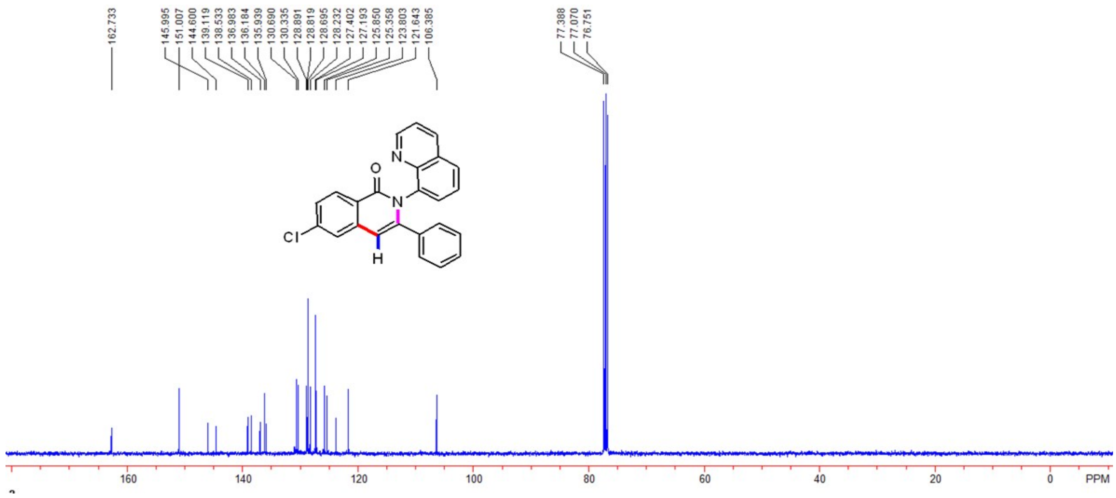

7-chloro-3-phenyl-2-(quinolin-8-yl)isoquinolin-1(*2H*)-one (4j)

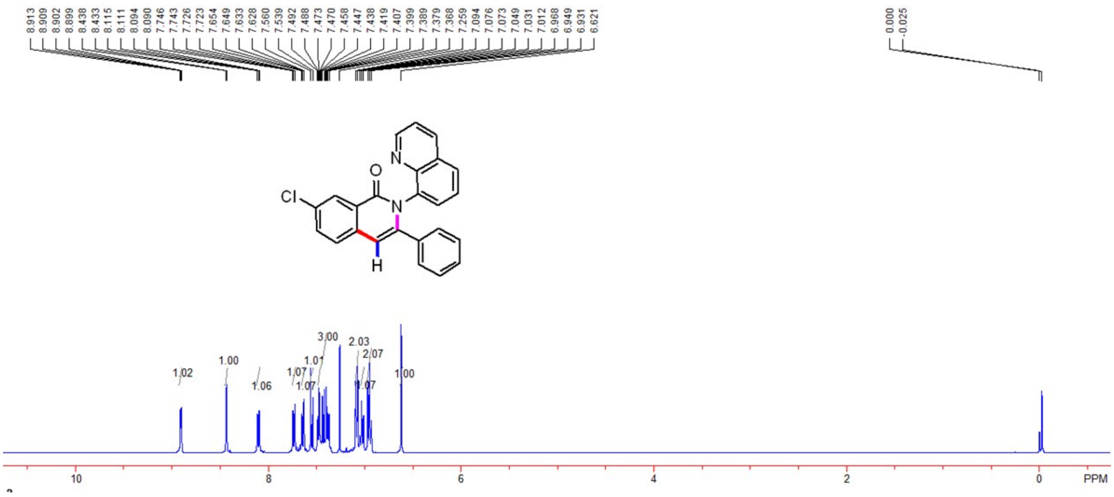

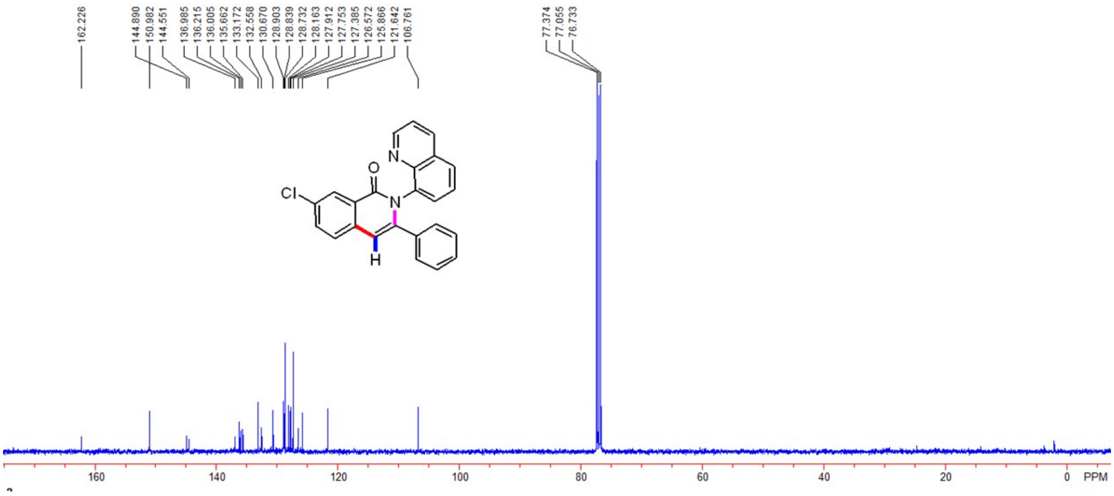

8-chloro-3-phenyl-2-(quinolin-8-yl)isoquinolin-1(*2H*)-one (4k)

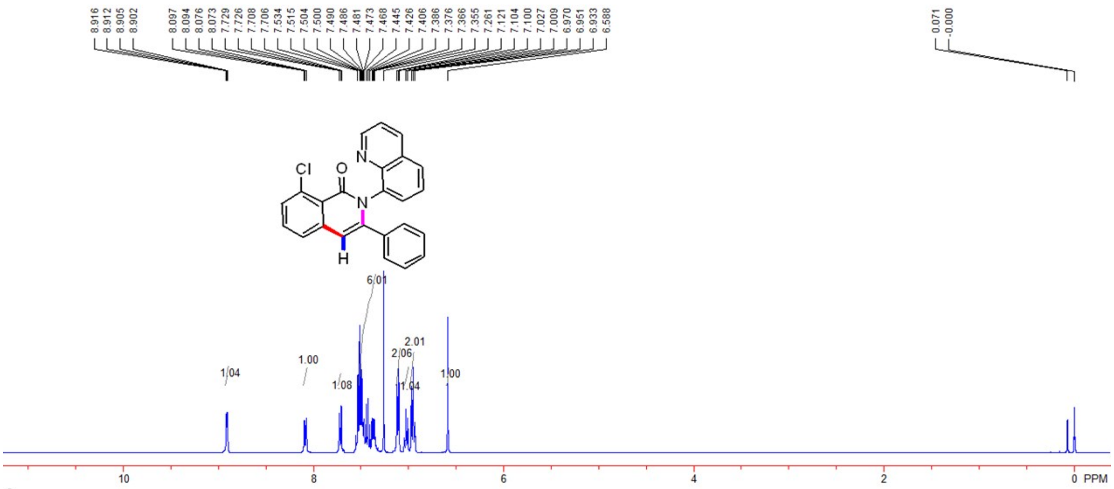

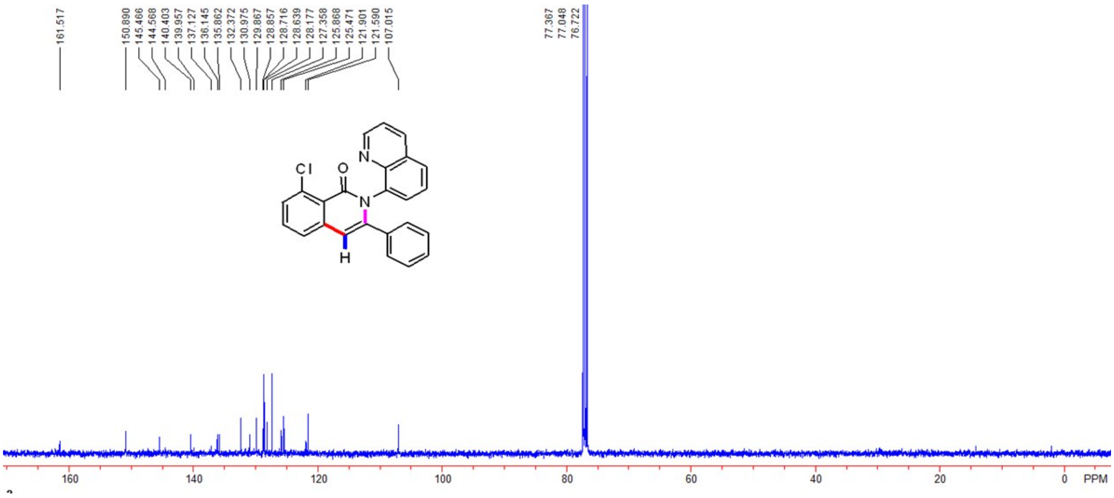

5-fluoro-3-phenyl-2-(quinolin-8-yl)isoquinolin-1(*2H*)-one **(4l)**

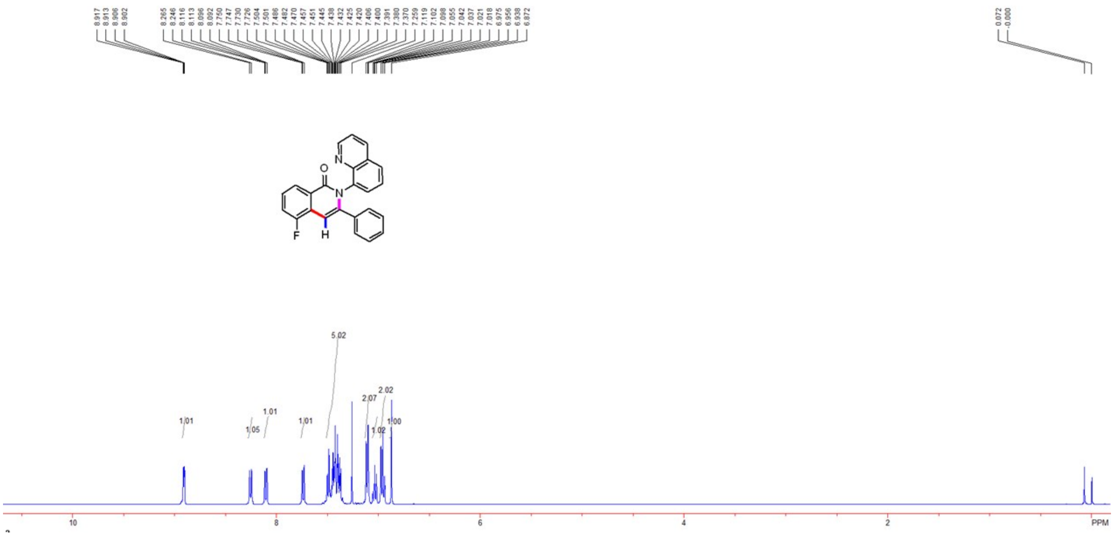

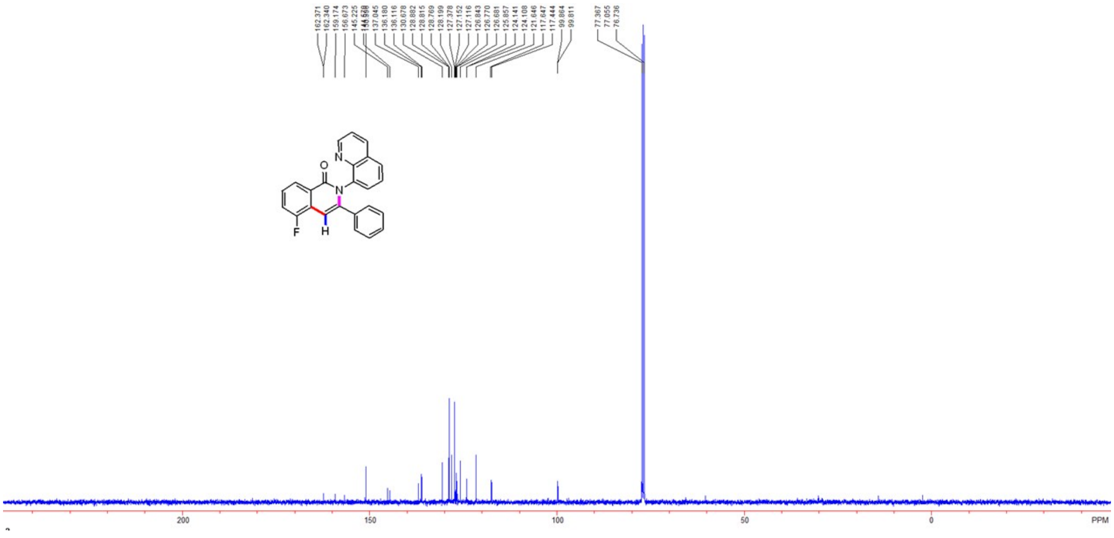

3-phenyl-2-(quinolin-8-yl)benzo[*h*]isoquinolin-1(*2H*)-one (4m)

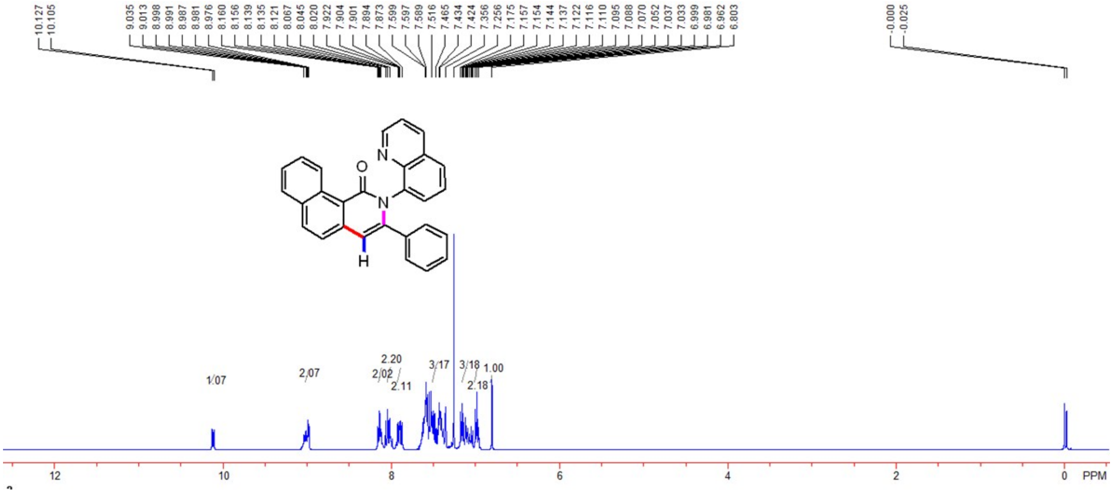

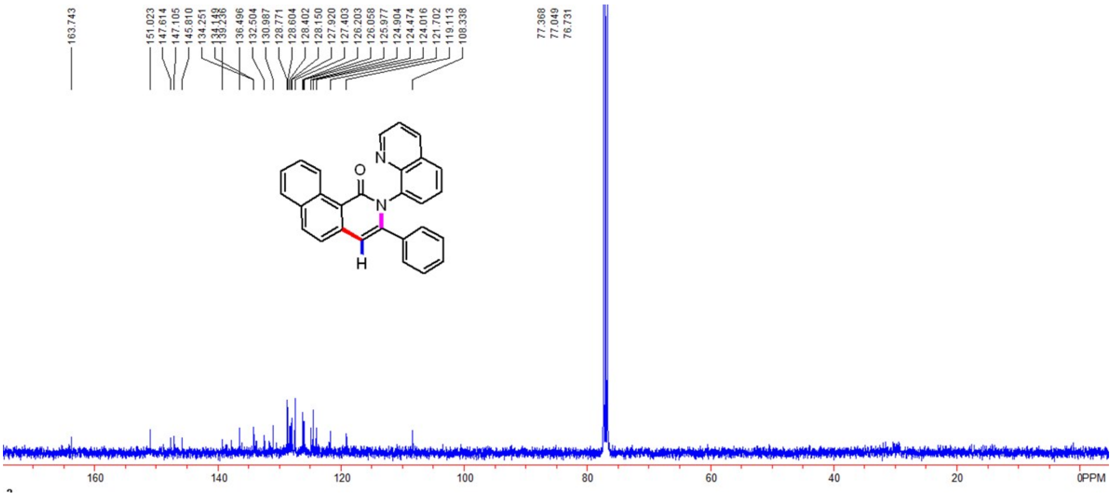

3-phenyl-2-(quinolin-8-yl)benzo[*g*]isoquinolin-1(*2H*)-one (4n)

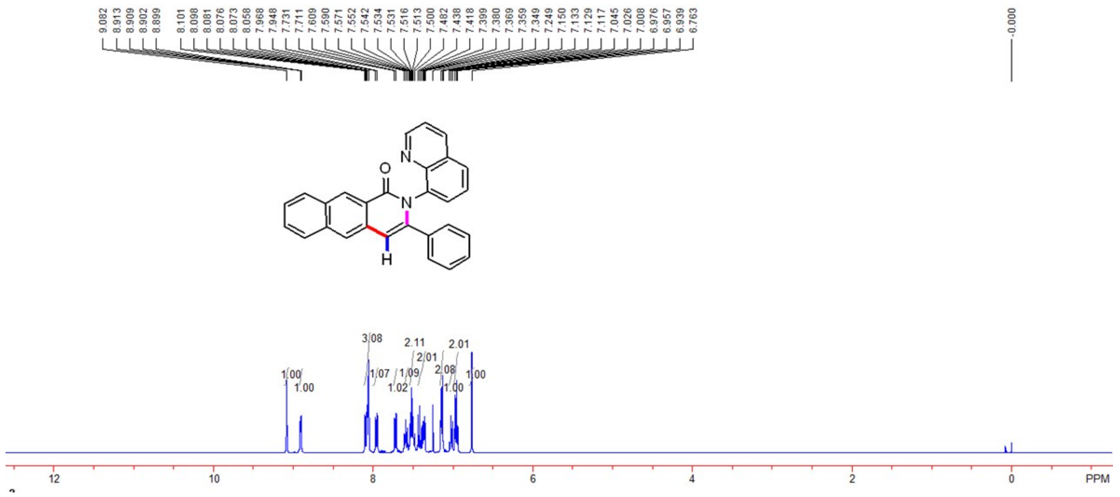

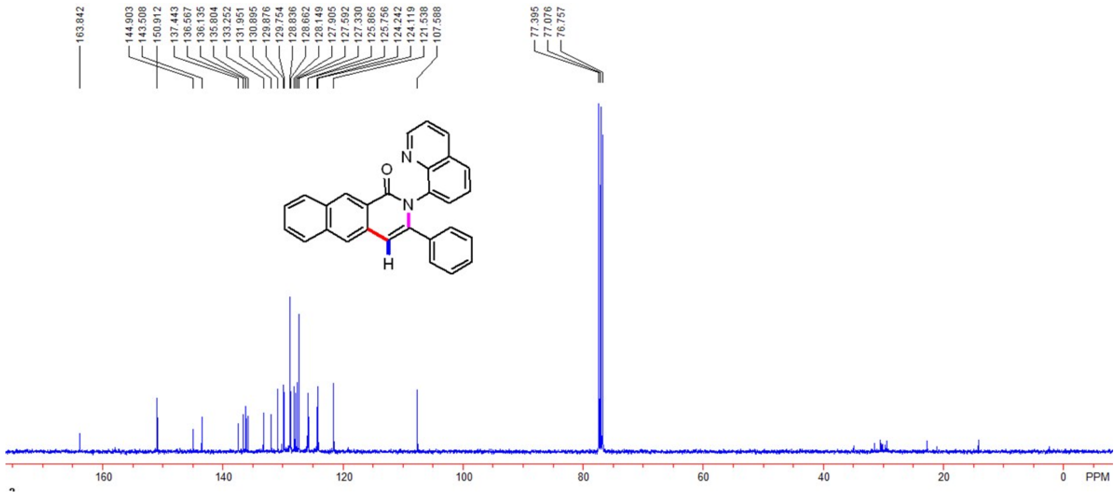

5-phenyl-6-(quinolin-8-yl)thieno[2,3-c]pyridin-7(*6H*)-one (4o)

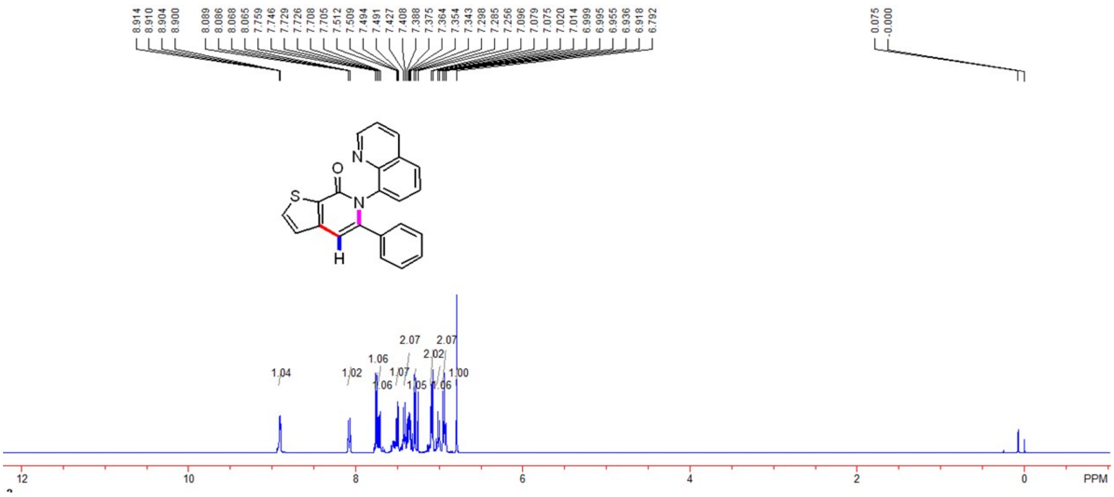

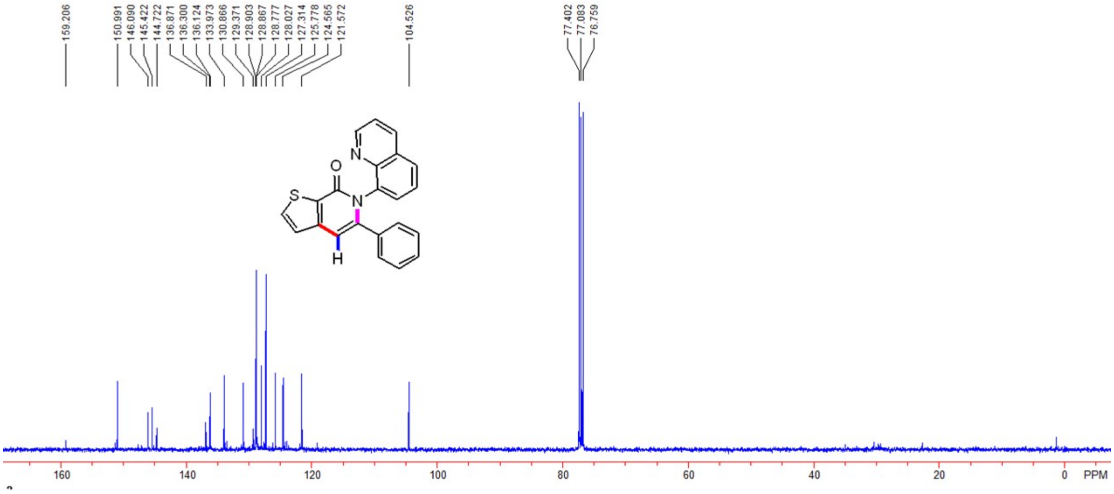

5-phenyl-6-(quinolin-8-yl)furo[2,3-c]pyridin-7(*6H*)-one (4p)

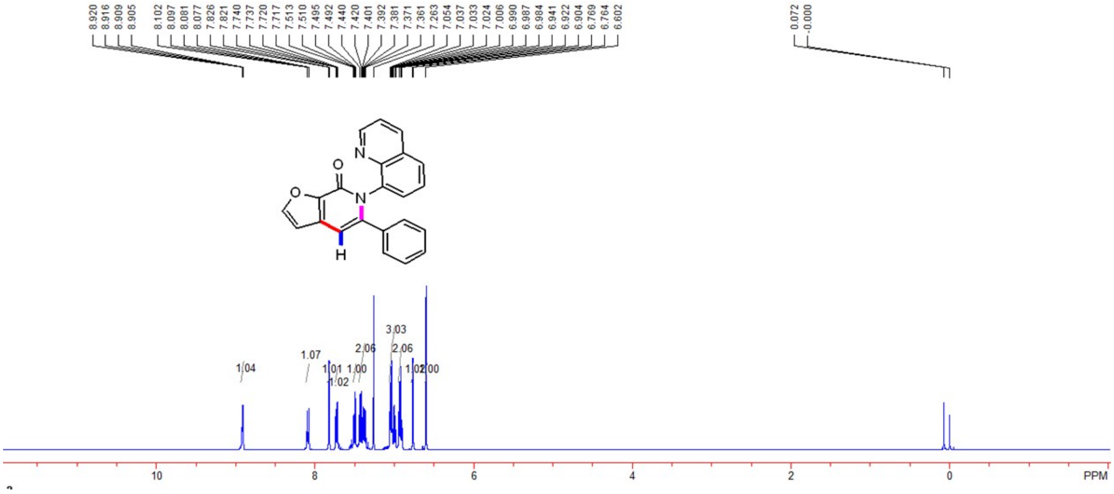

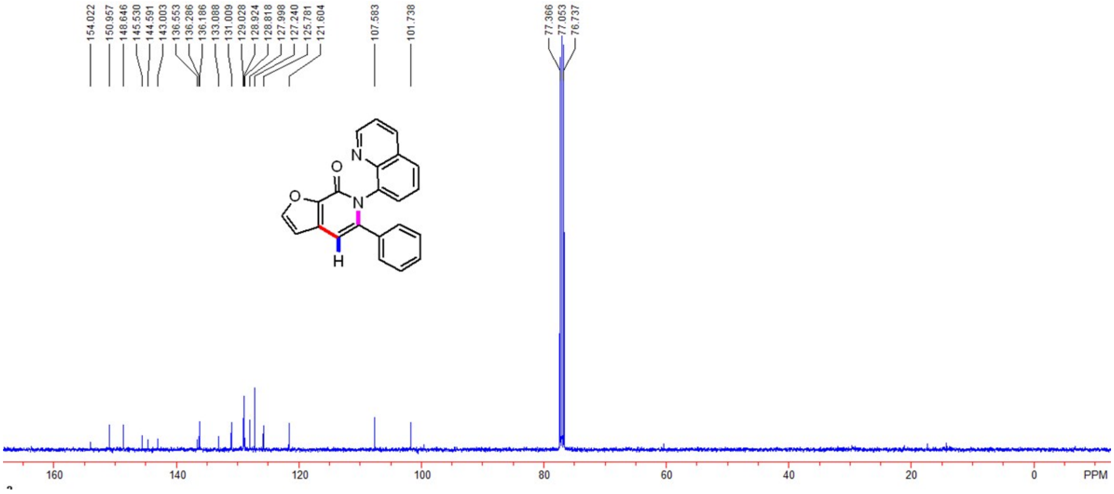

3-phenyl-2-(quinolin-8-yl)-5,6,7,8-tetrahydroisoquinolin-1(*2H*)-one (6a)

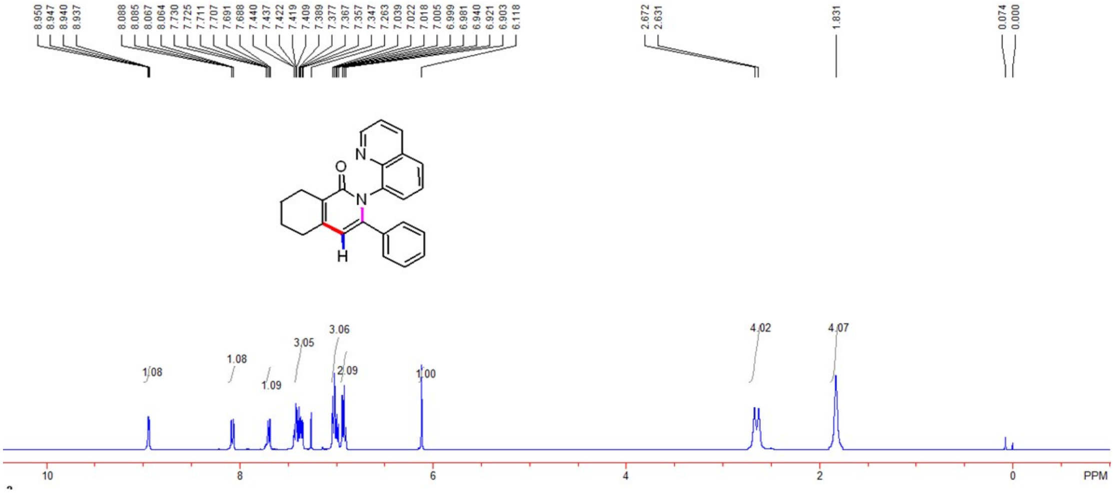

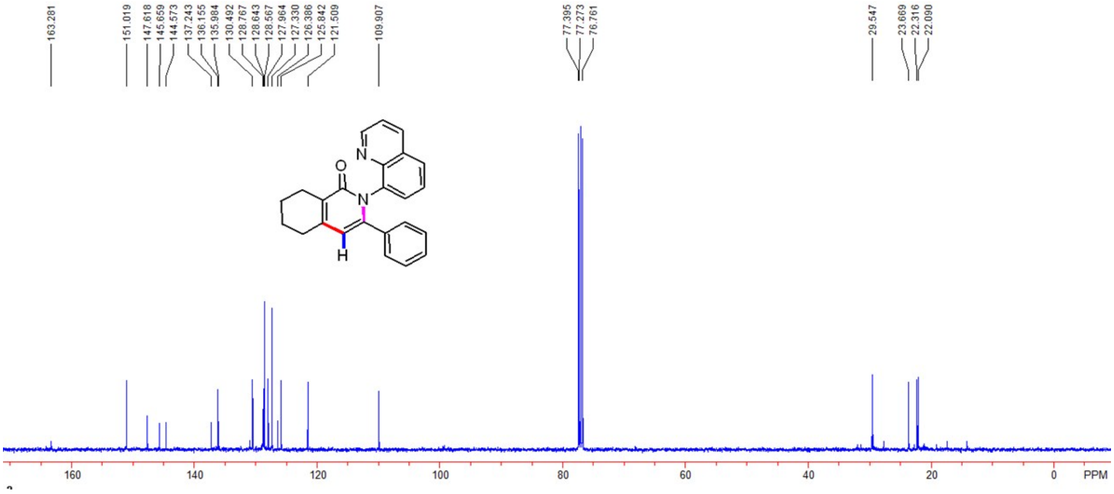

3,4-dimethyl-6-phenyl-1-(quinolin-8-yl)pyridin-2(*1H*)-one (6b)

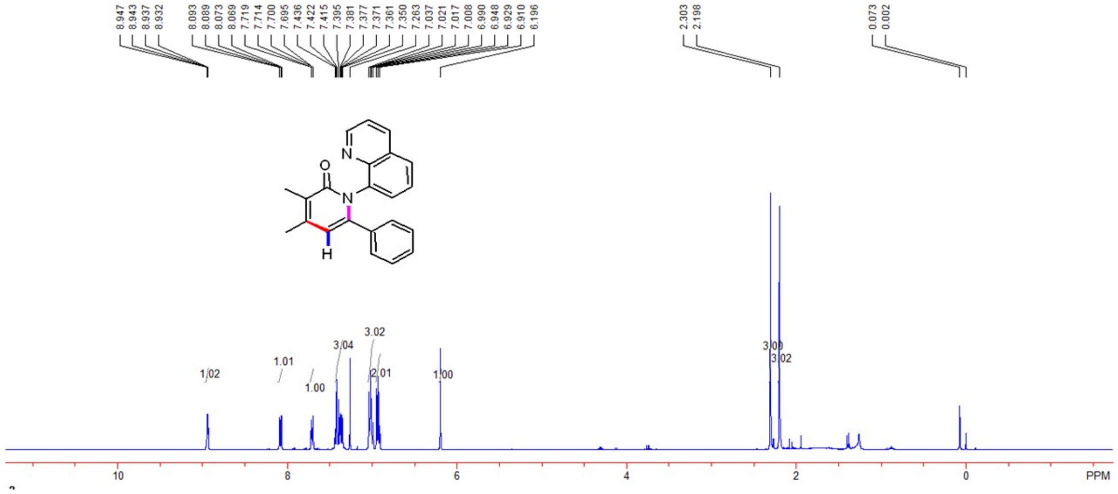

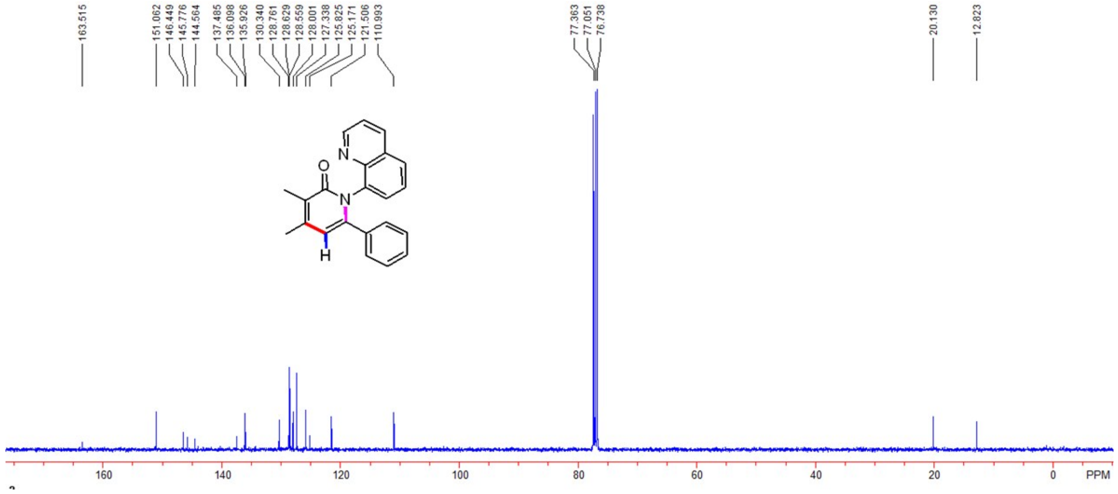

4-ethyl-3-methyl-6-phenyl-1-(quinolin-8-yl)pyridin-2(*1H*)-one (6c)

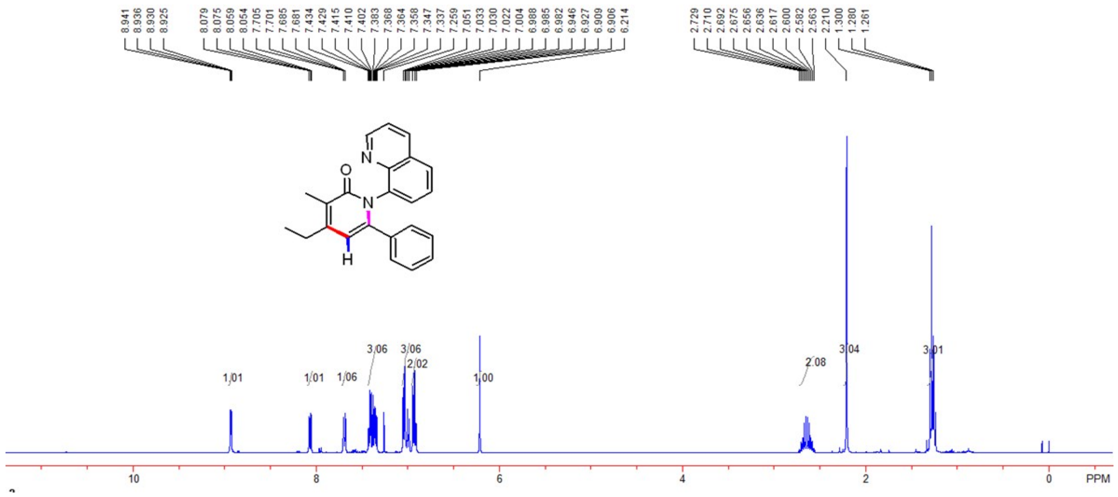

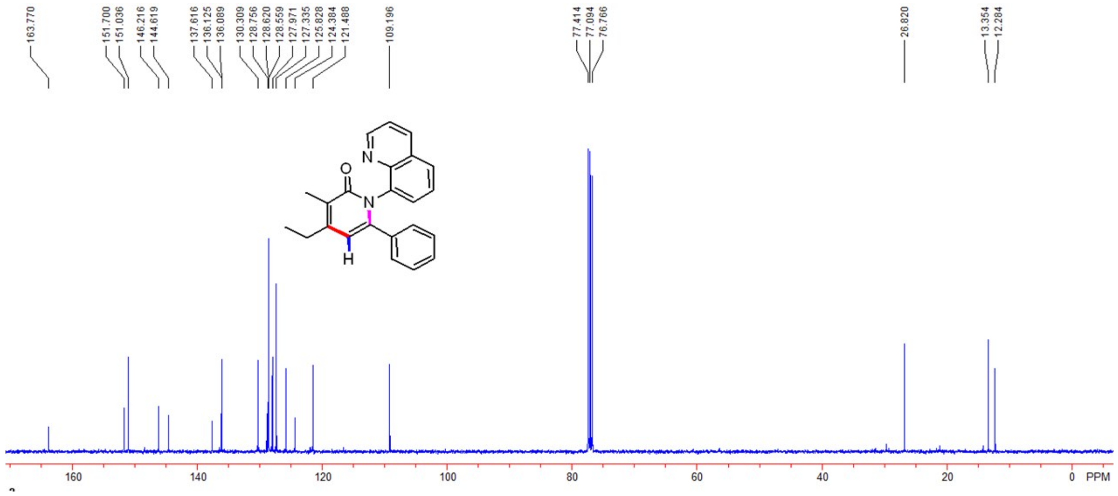

3-methyl-4,6-diphenyl-1-(quinolin-8-yl)pyridin-2(*1H*)-one **(6d)**

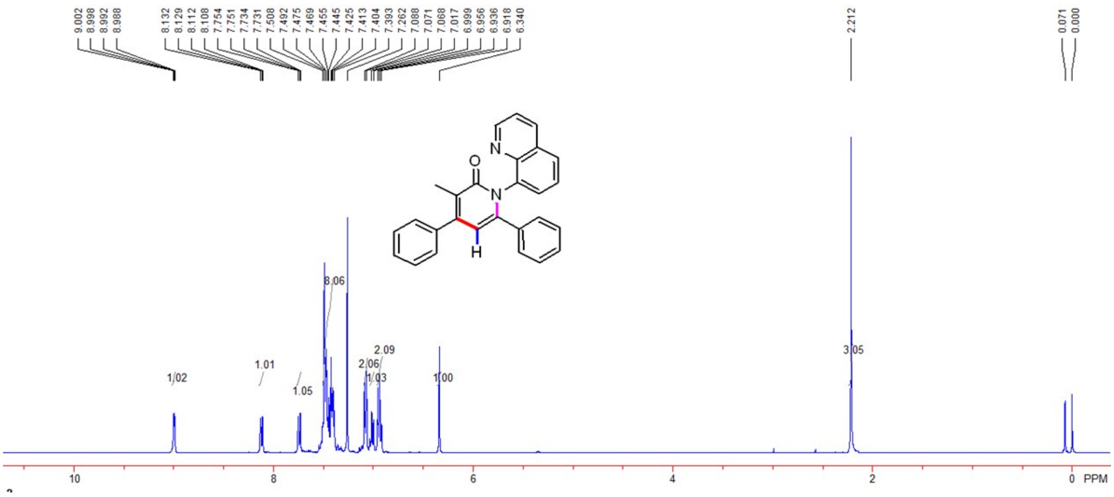

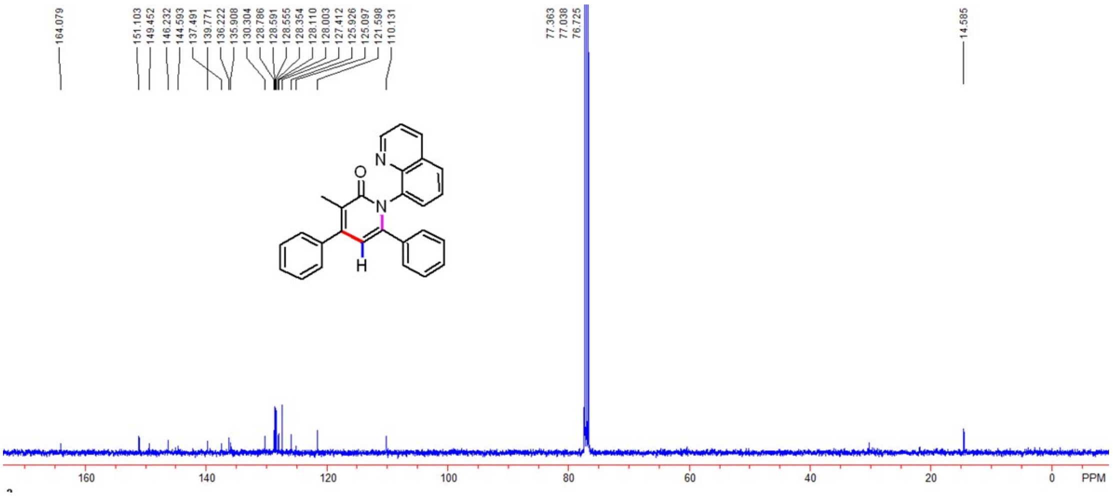

3-(2-chlorophenyl)-6-phenyl-1-(quinolin-8-yl)pyridin-2(*1H*)-one (6e)

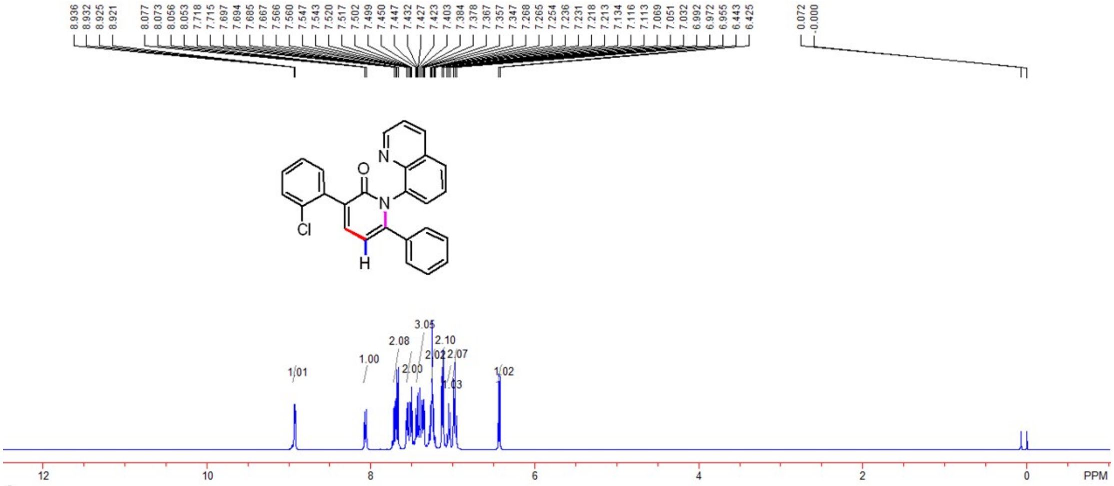

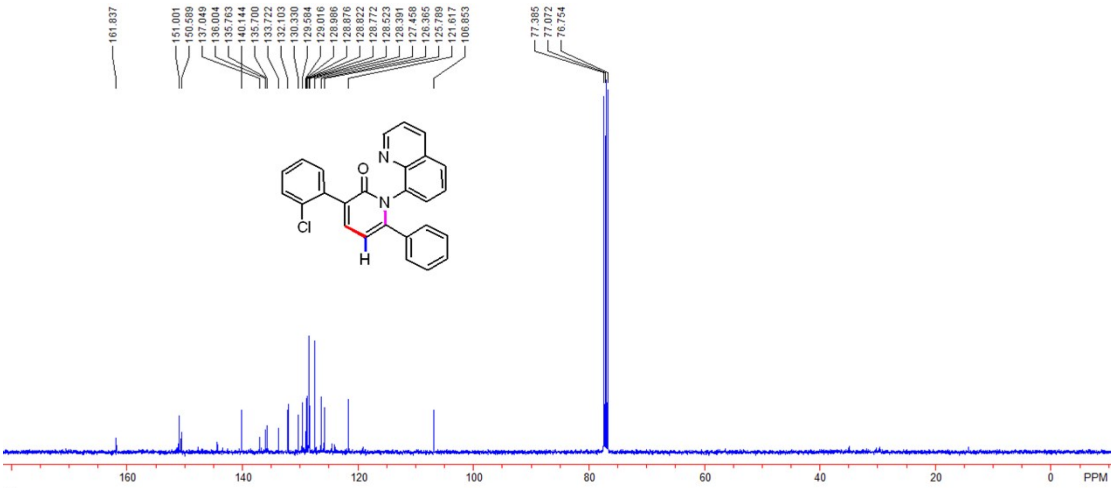

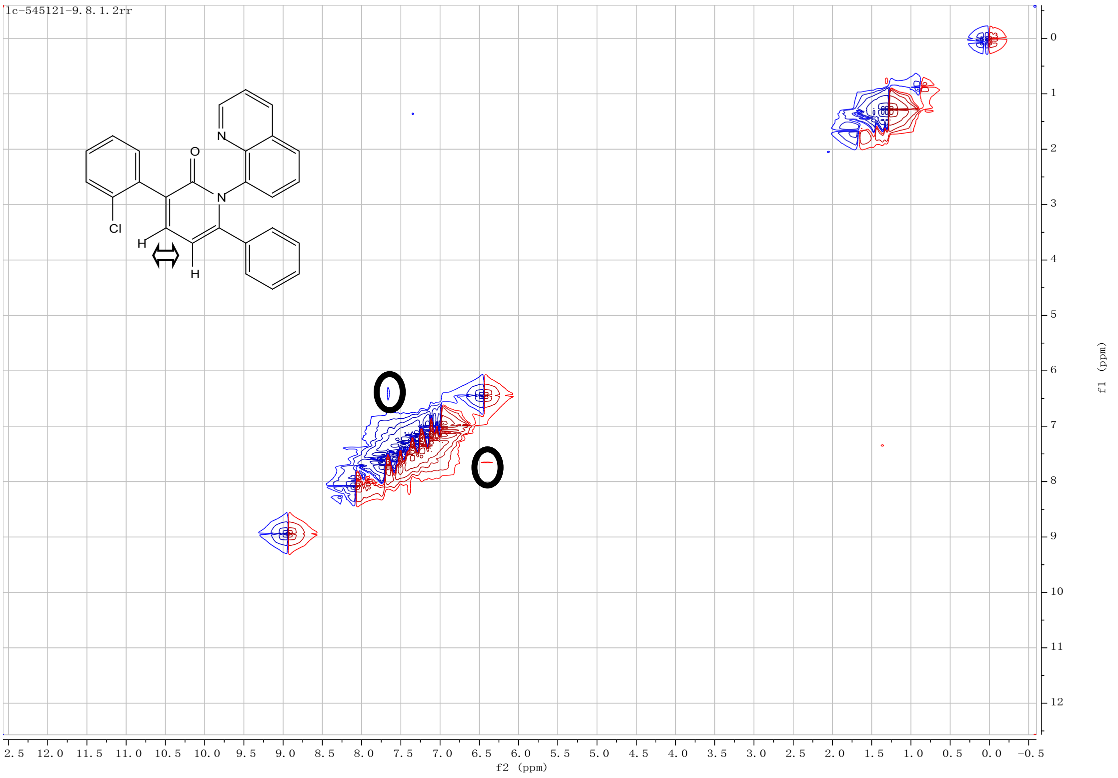

3-(3-chlorophenyl)-6-phenyl-1-(quinolin-8-yl)pyridin-2(*1H*)-one (6f)

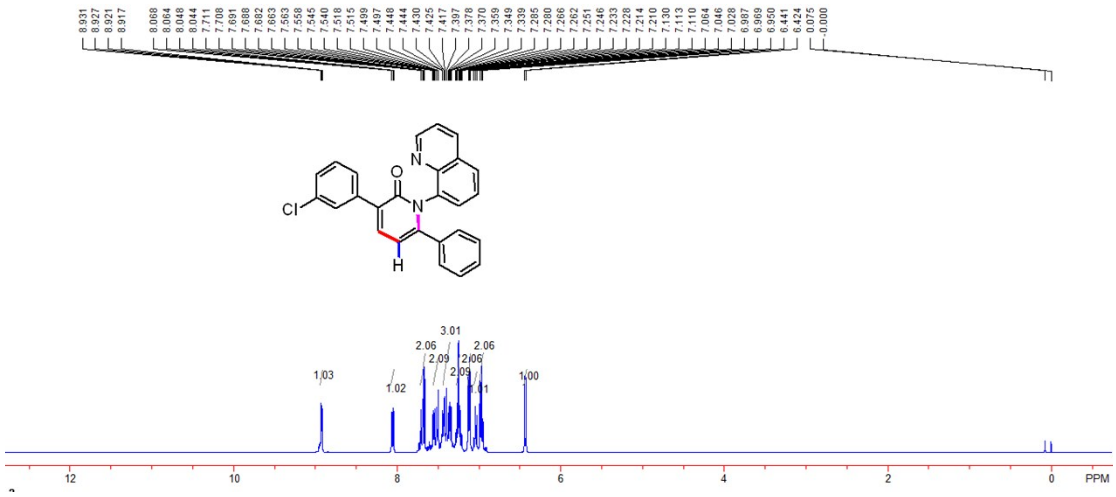

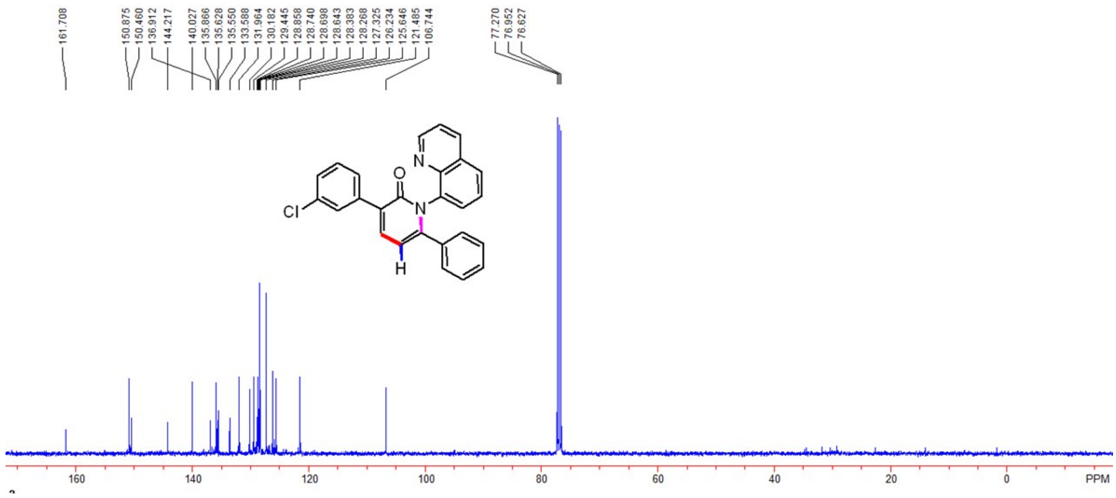

3-(4-chlorophenyl)-6-phenyl-1-(quinolin-8-yl)pyridin-2(*1H*)-one (6g)

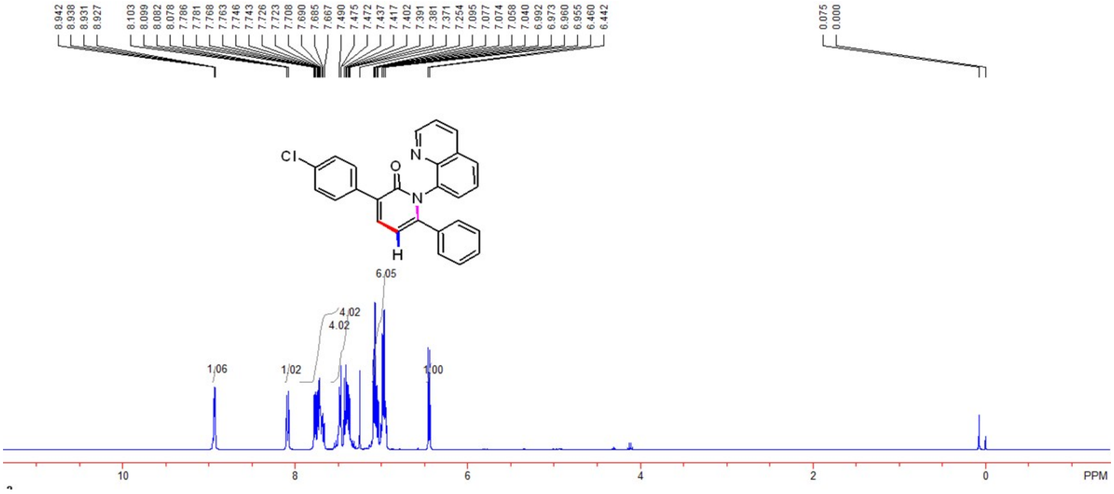

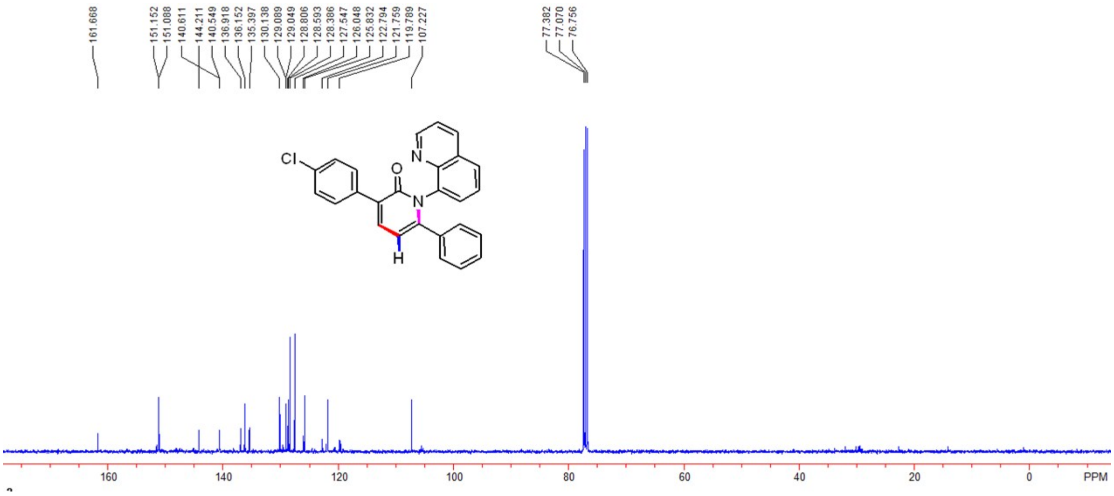

4,6-diphenyl-1-(quinolin-8-yl)pyridin-2(*1H*)-one (6h)

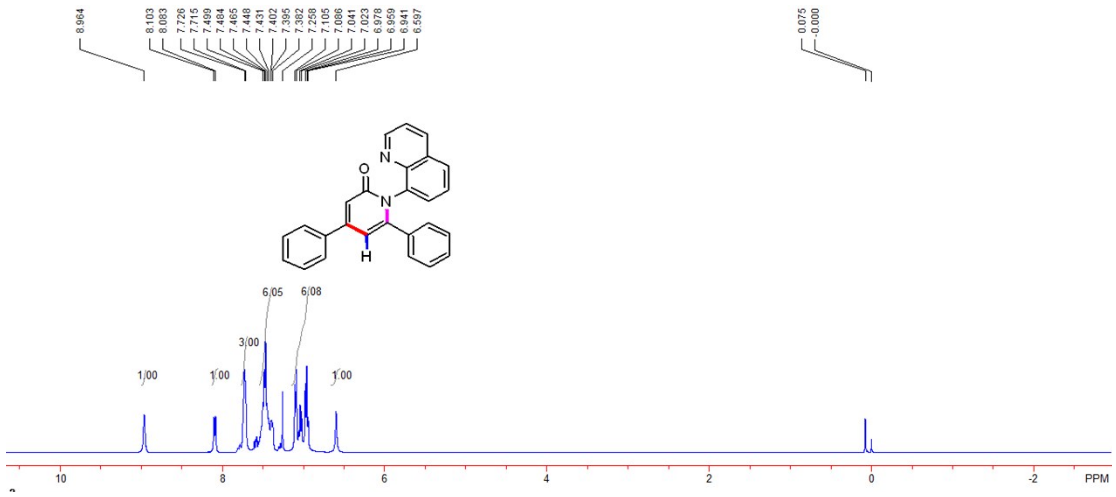

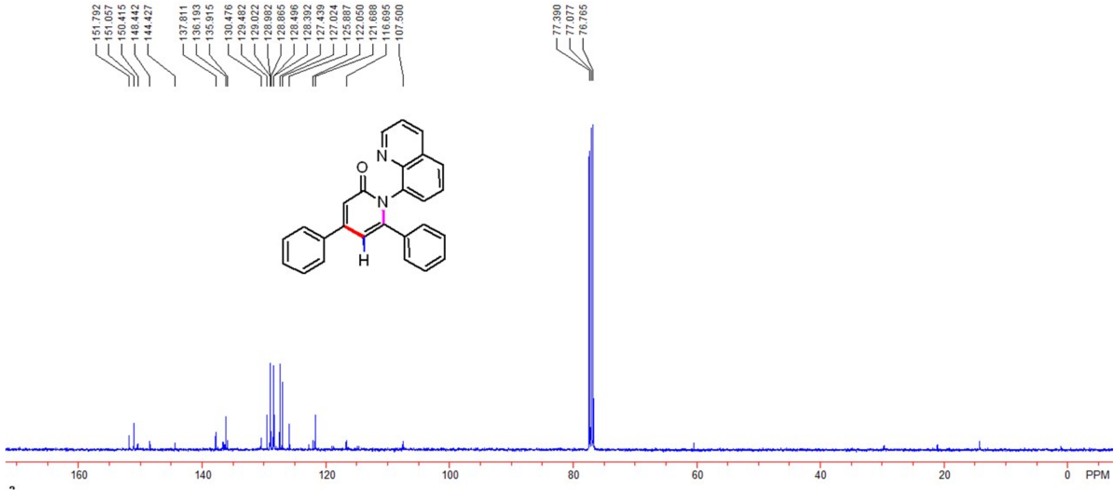

3,6-diphenyl-1-(quinolin-8-yl)pyridin-2(*1H*)-one (6i)

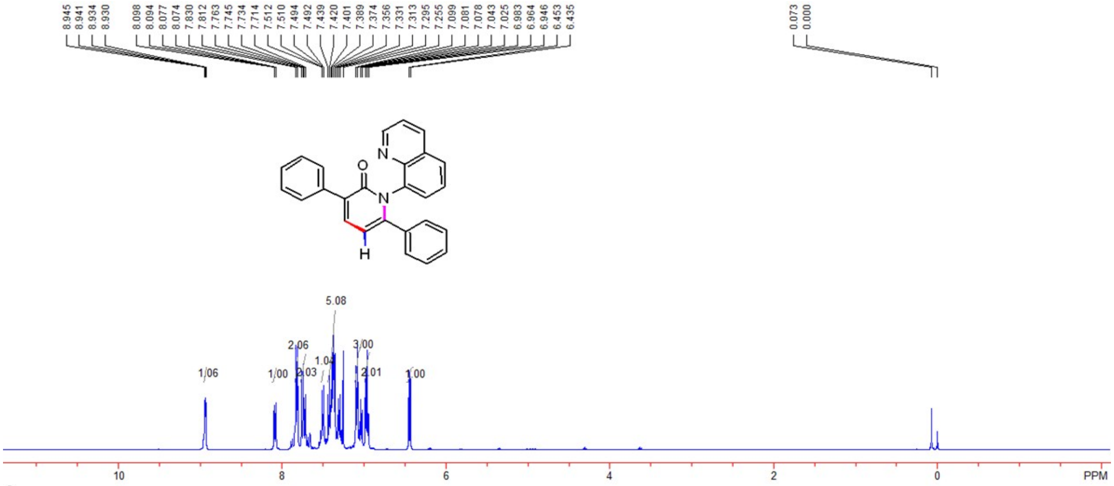

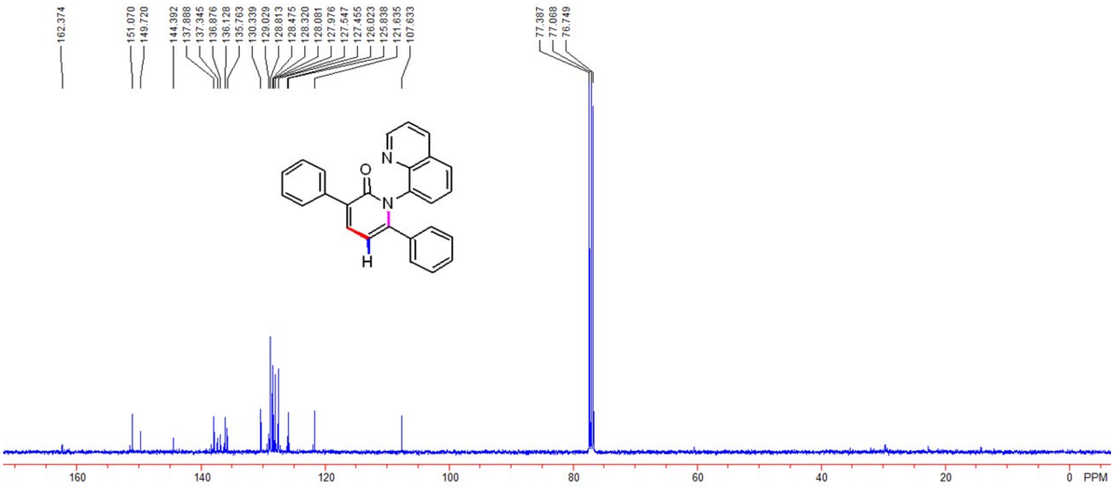

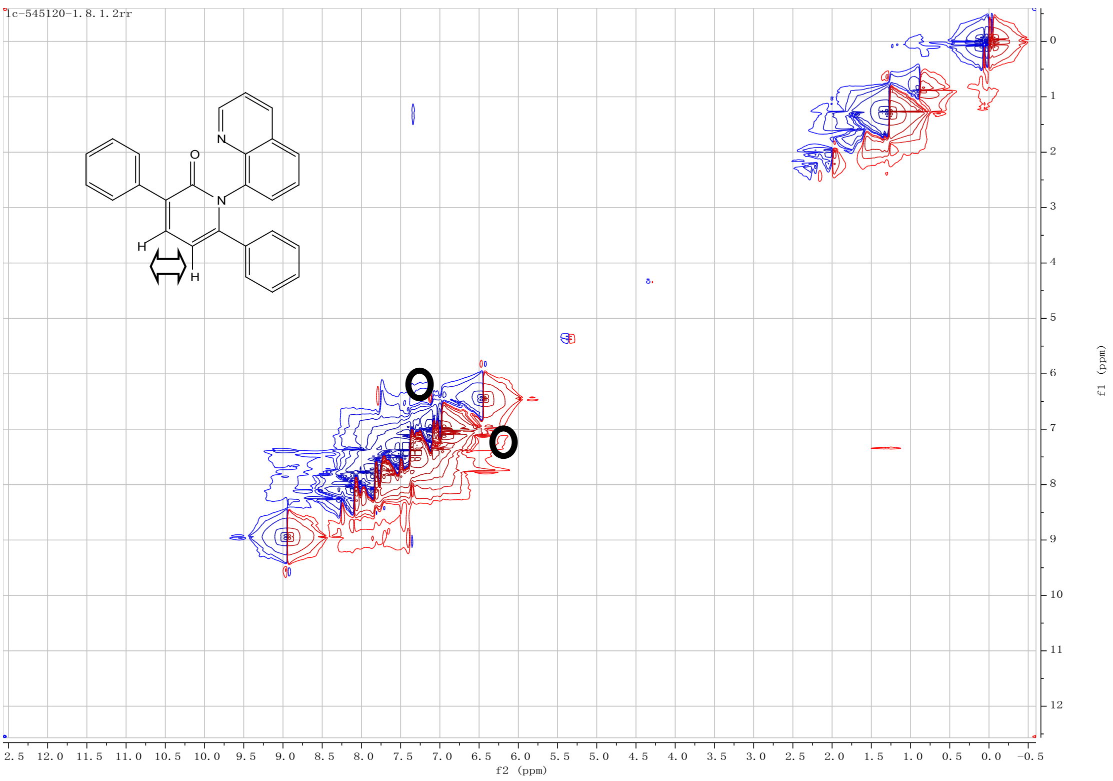

3-methyl-6-phenyl-1-(quinolin-8-yl)pyridin-2(*1H*)-one **(6j)**

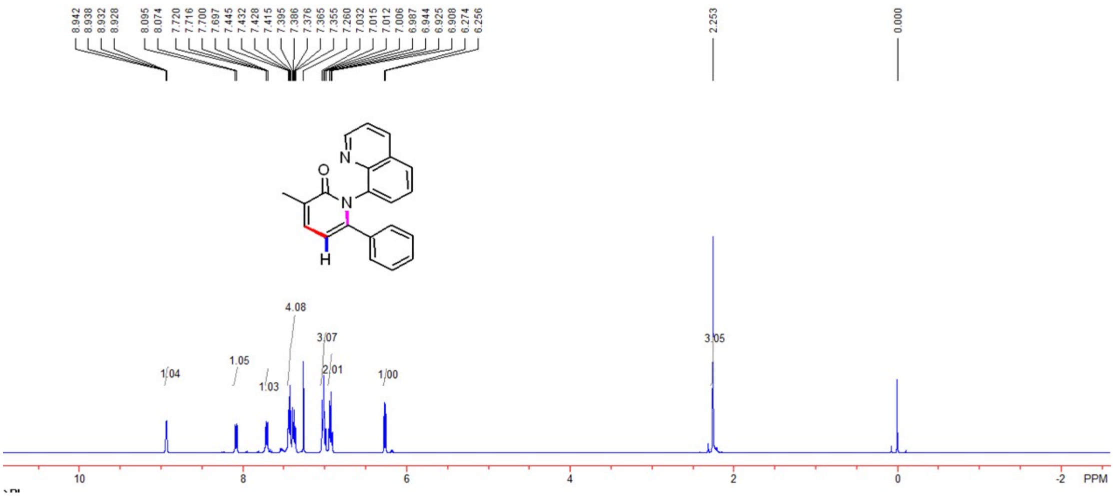

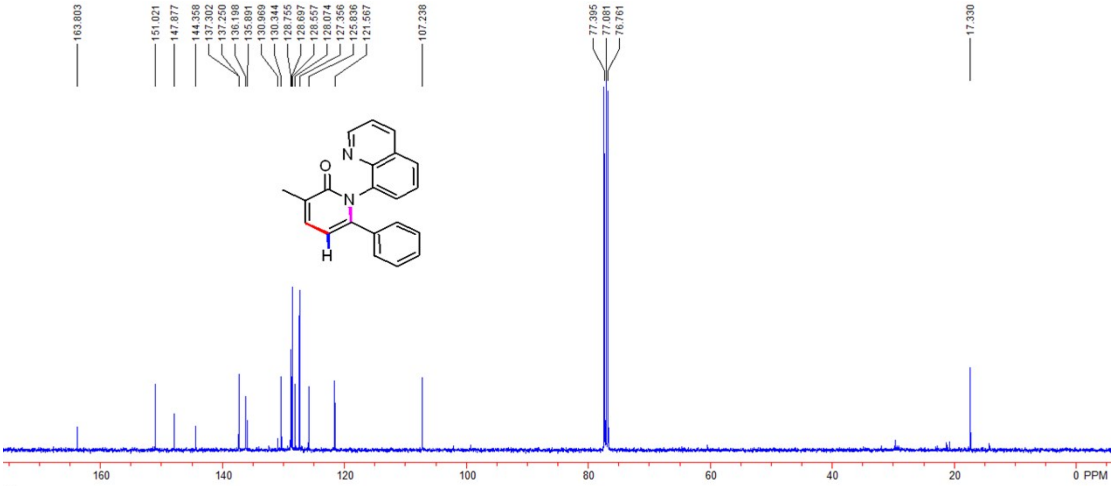

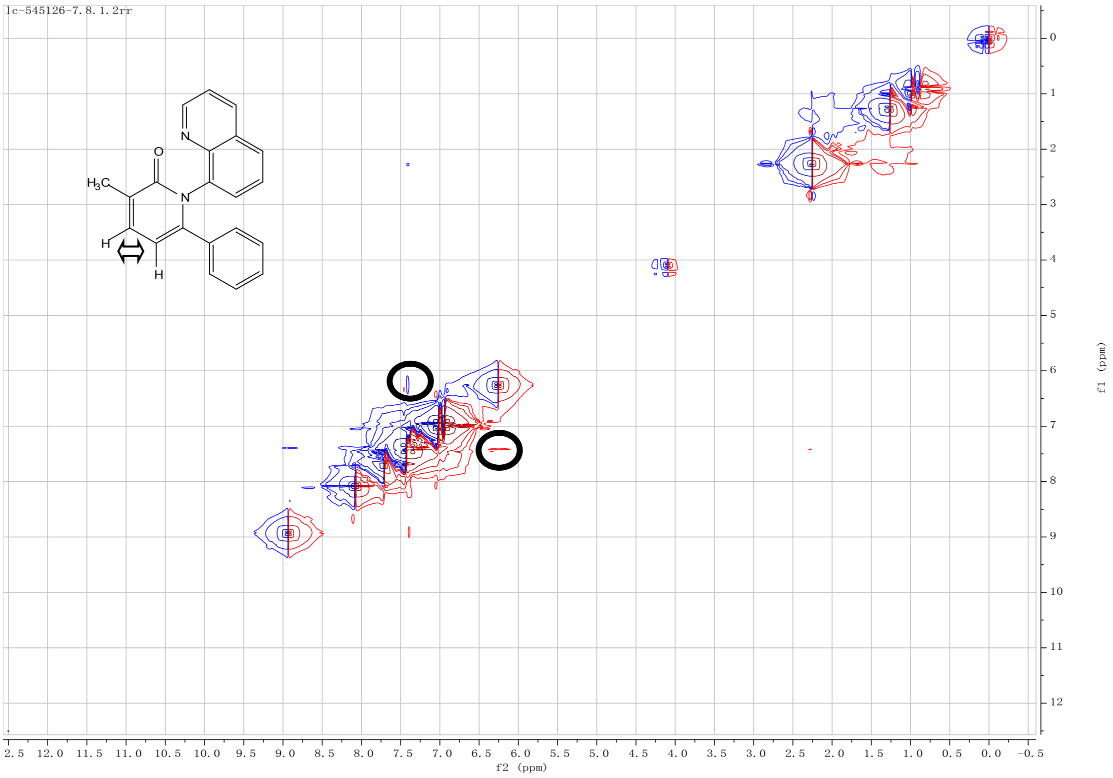

3-phenyl-2-(quinolin-8-yl)-4-(trimethylsilyl)isoquinolin-1(*2H*)-one (7a)

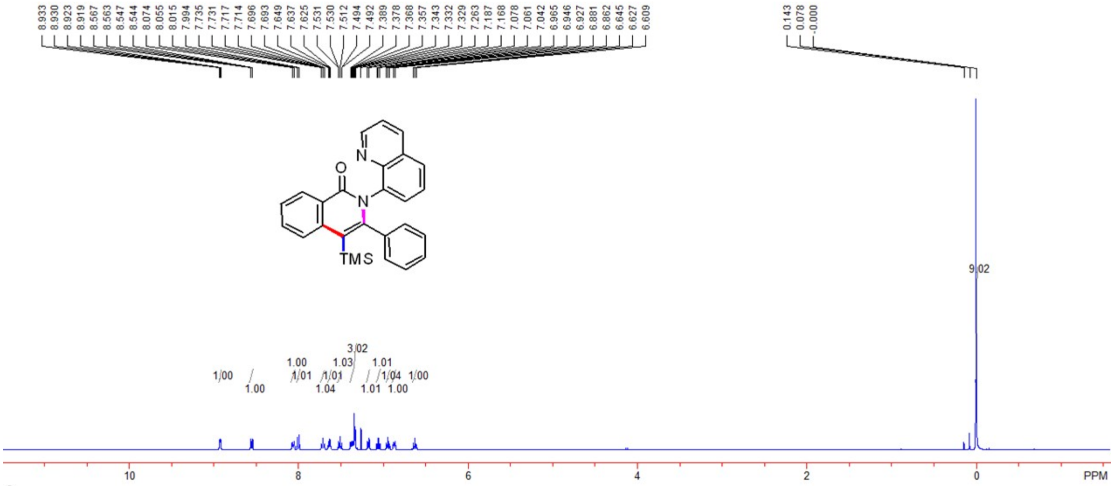

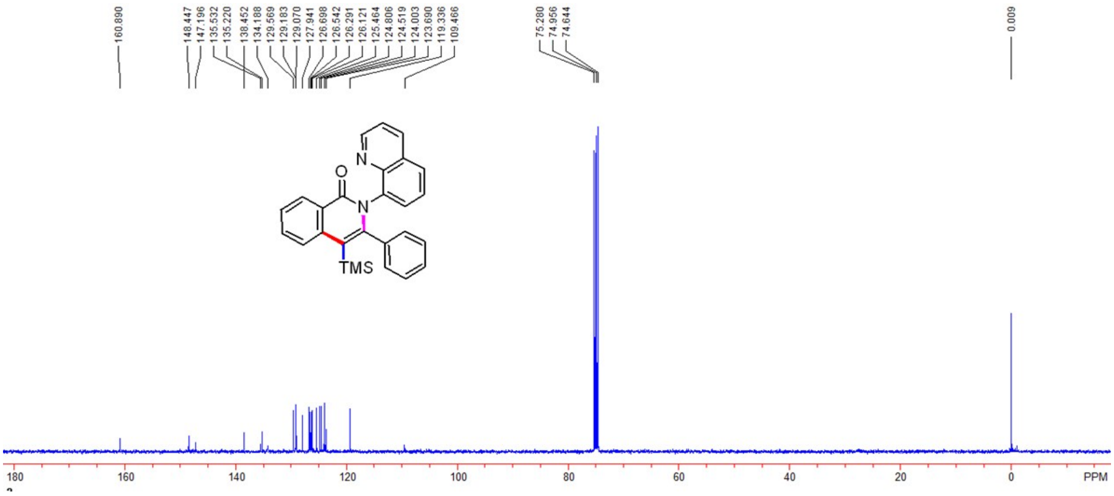

3-(4-bromophenyl)-2-(quinolin-8-yl)-4-(trimethylsilyl)isoquinolin-1(*2H*)-one (7b)

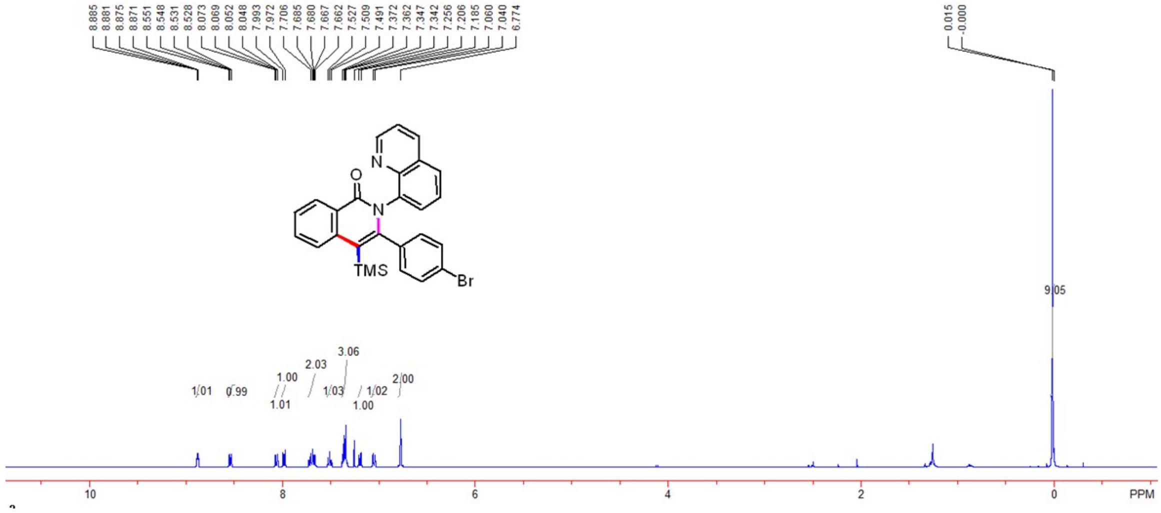

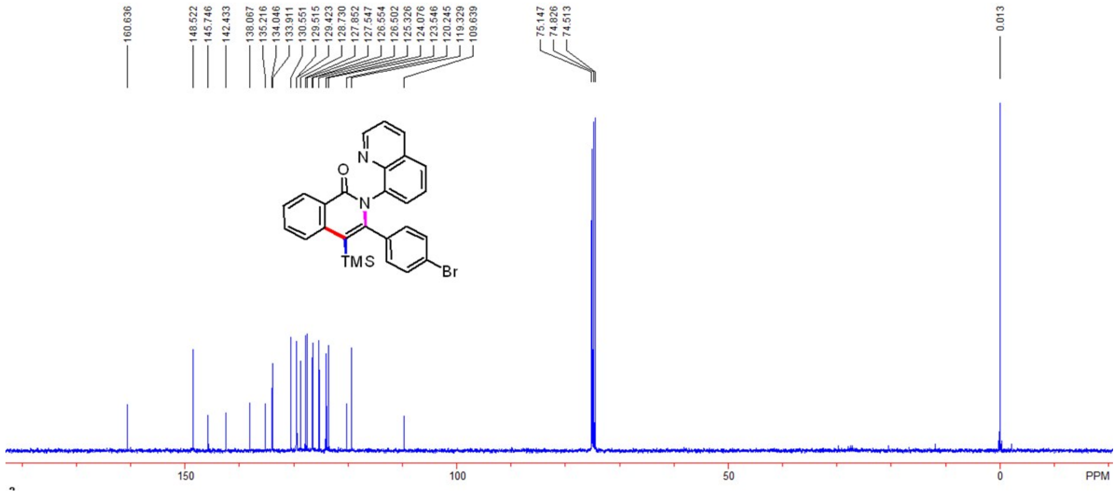

3-(3-chlorophenyl)-2-(quinolin-8-yl)-4-(trimethylsilyl)isoquinolin-1(*2H*)-one (7c)

4-(3-chlorophenyl)-2-(quinolin-8-yl)-3-(trimethylsilyl)isoquinolin-1(2H)-one (7c′)

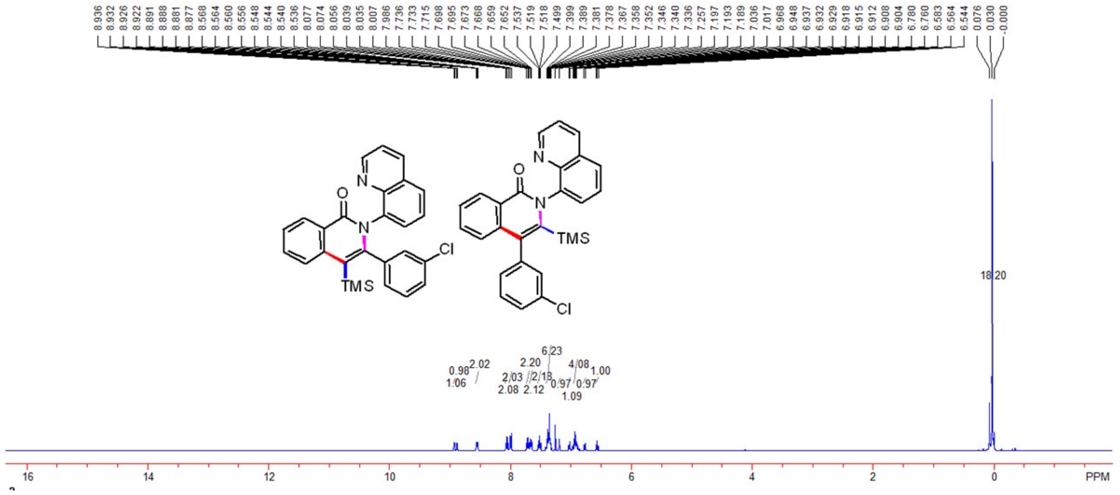

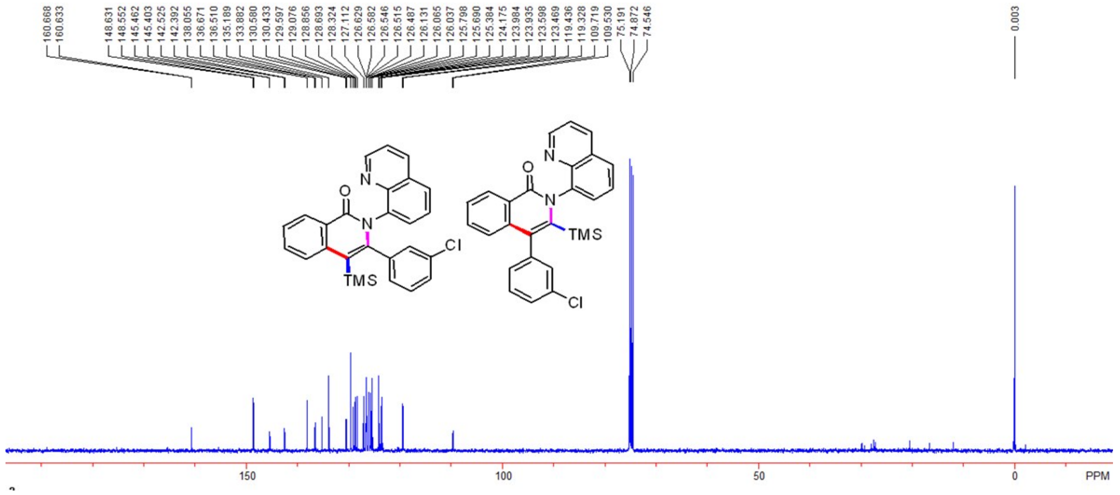

3-(pyridin-2-yl)-2-(quinolin-8-yl)-4-(trimethylsilyl)isoquinolin-1(*2H*)-one (7d)

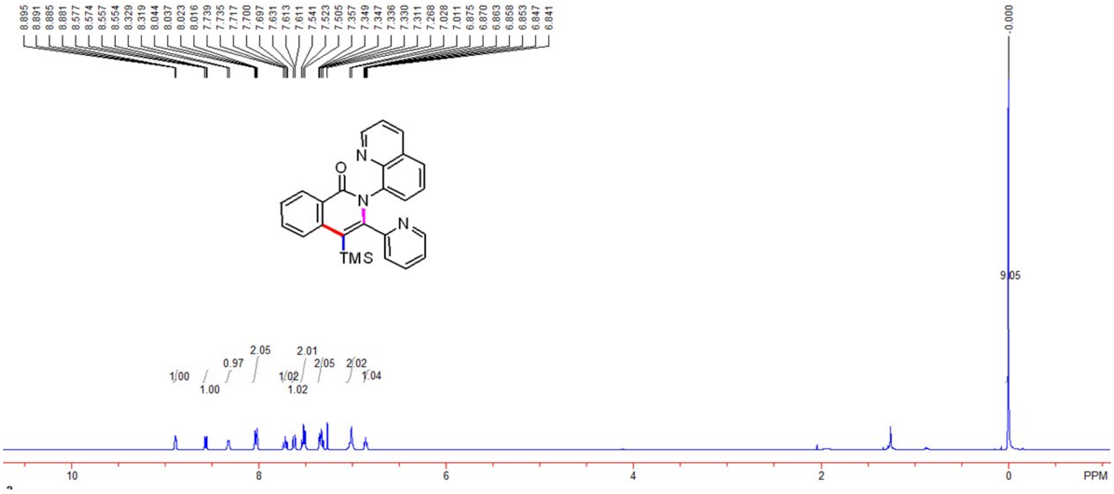

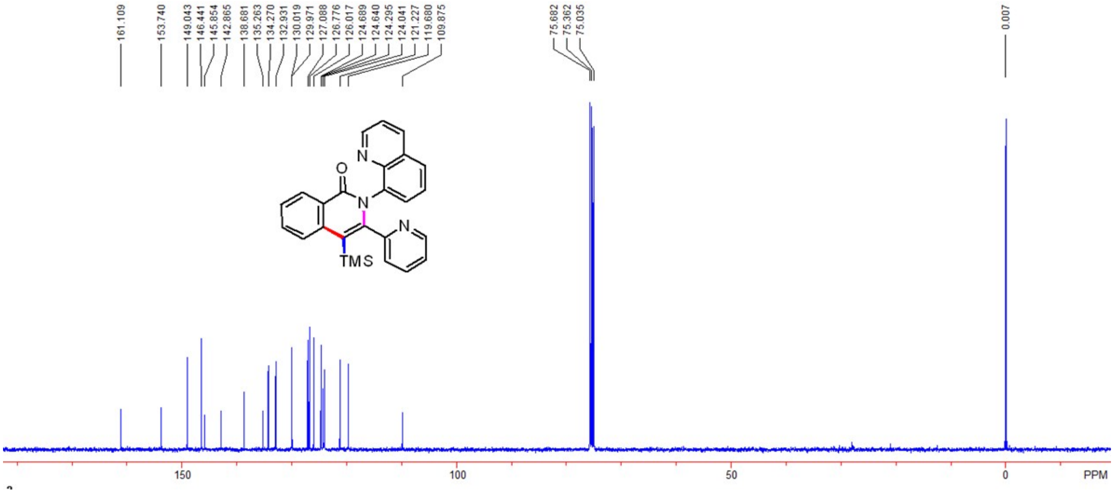

3-(prop-1-en-2-yl)-2-(quinolin-8-yl)-4-(trimethylsilyl)isoquinolin-1(*2H*)-one (7e)
4-(prop-1-en-2-yl)-2-(quinolin-8-yl)-3-(trimethylsilyl)isoquinolin-1(*2H*)-one (7e′)

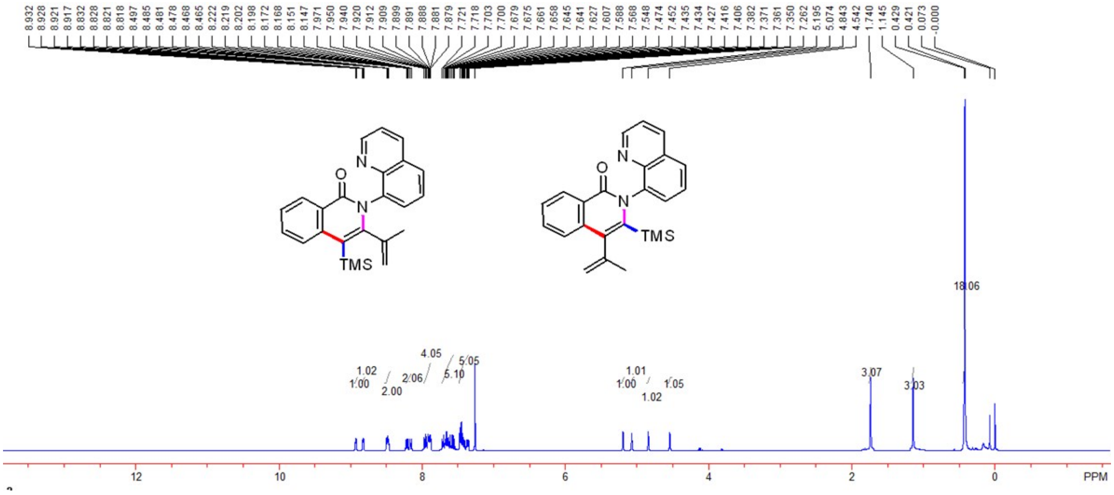

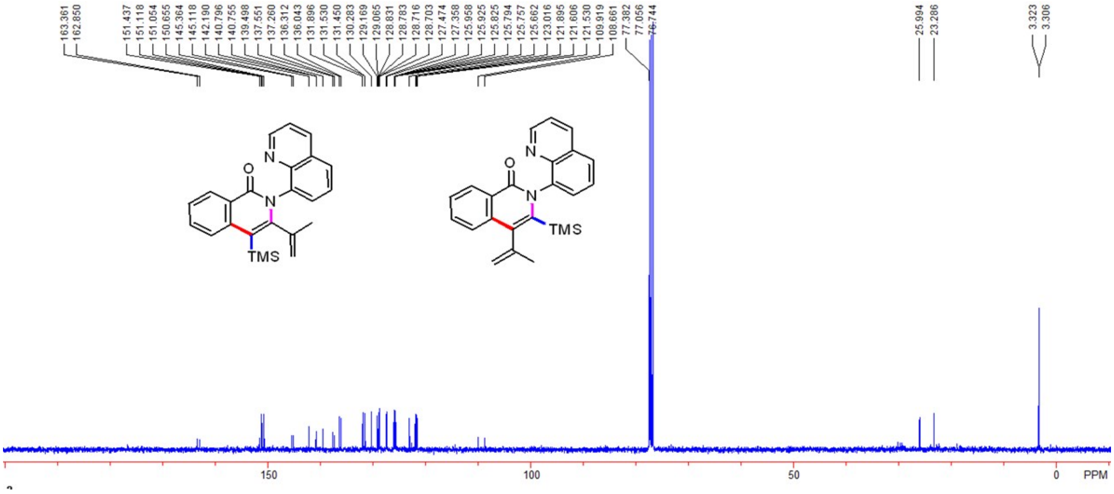

7-iodo-3-phenyl-2-(quinolin-8-yl)-4-(trimethylsilyl)isoquinolin-1(*2H*)-one (7f)

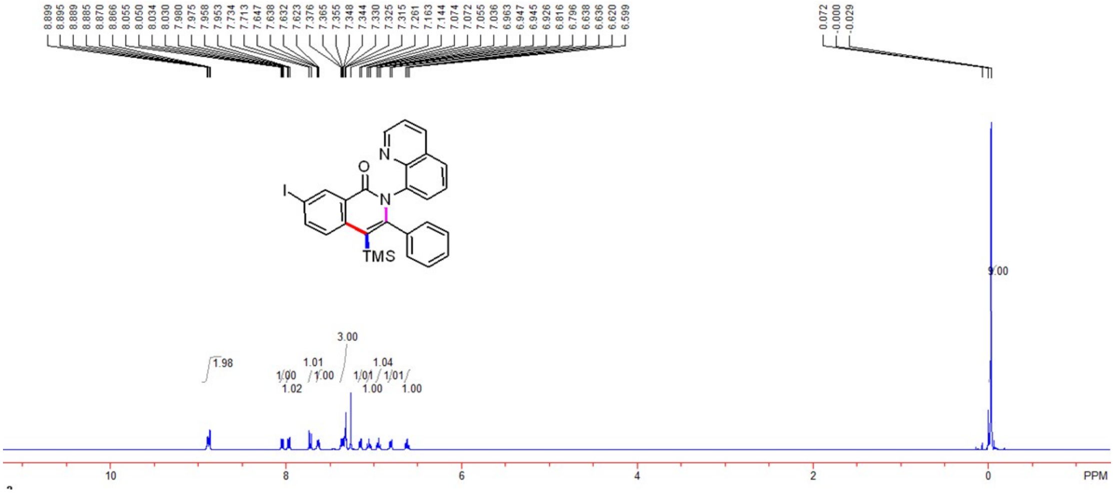

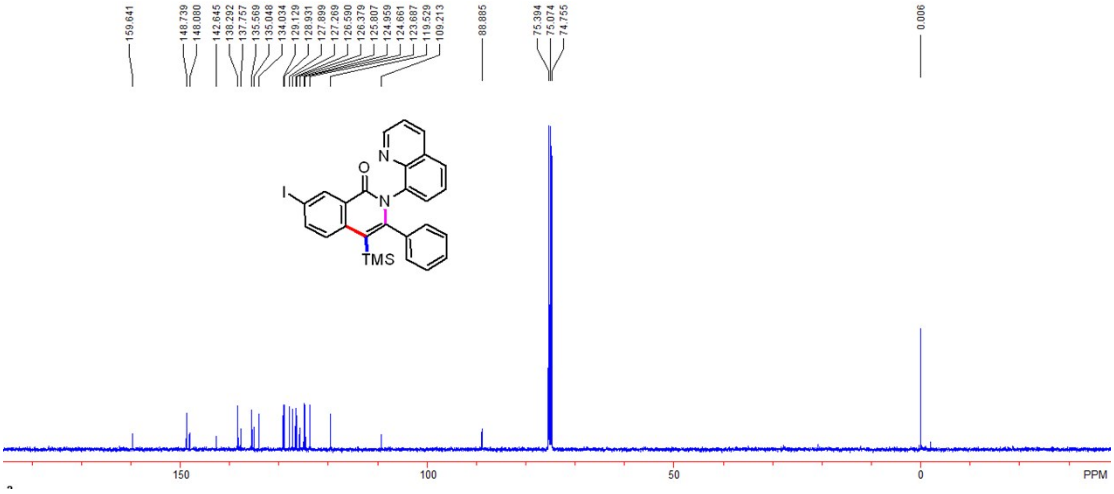

7-chloro-3-phenyl-2-(quinolin-8-yl)-4-(trimethylsilyl)isoquinolin-1(*2H*)-one (7g)

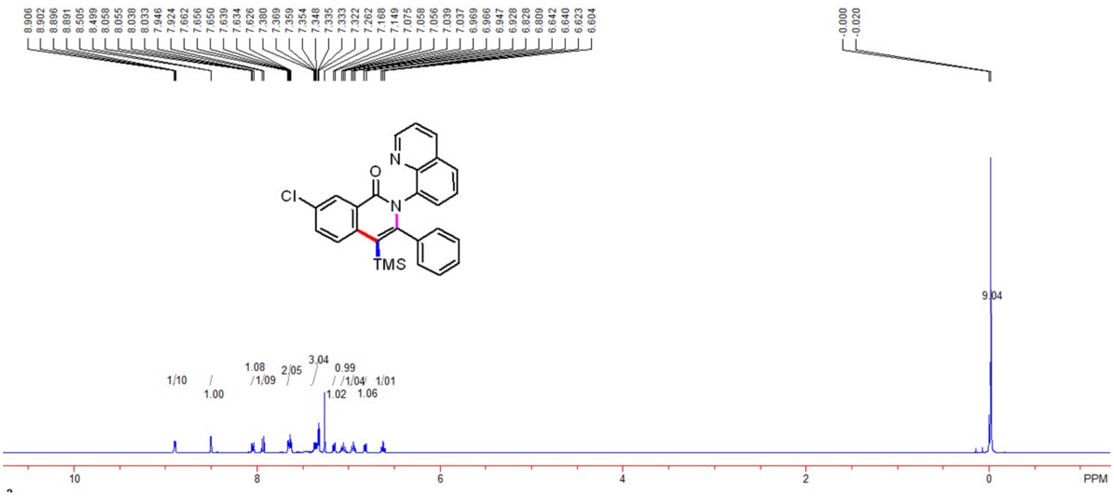

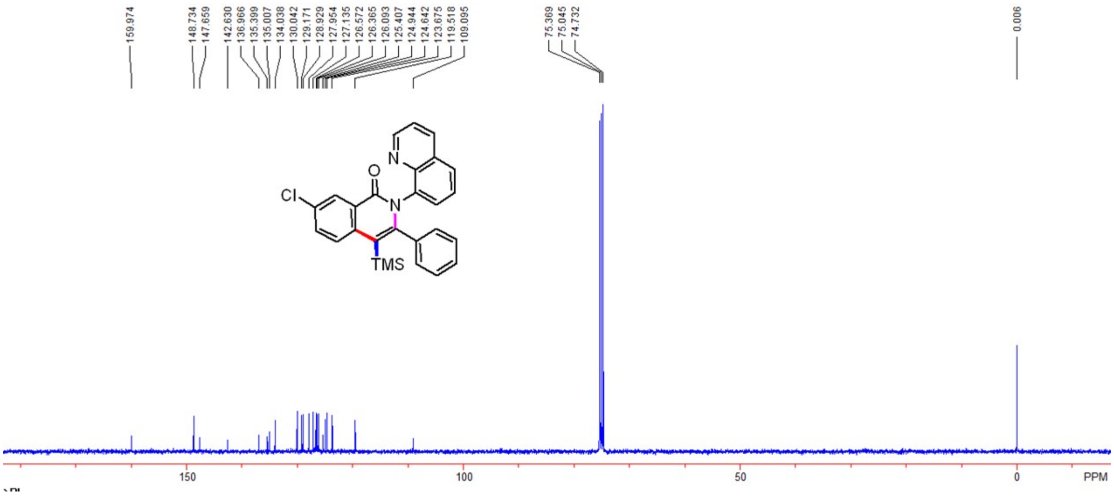

6-phenyl-5-(quinolin-8-yl)-7-(trimethylsilyl)thieno[3,2-c]pyridin-4(*5H*)-one (7h)

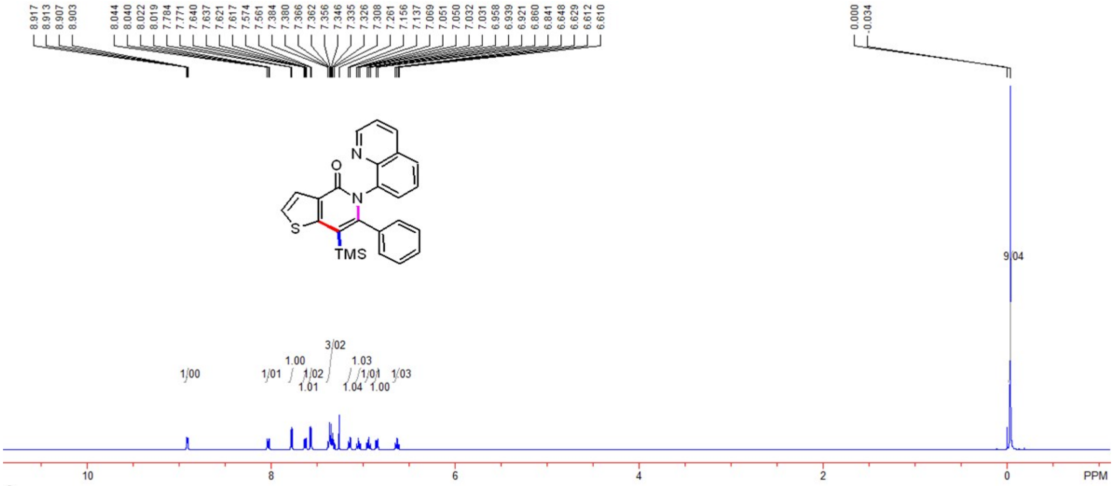

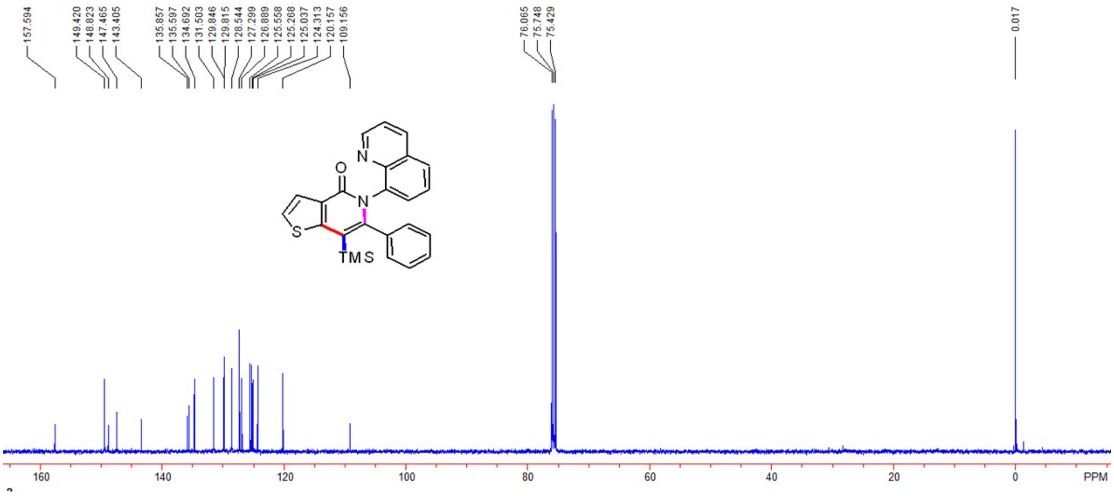

3-methyl-6-phenyl-1-(quinolin-8-yl)-5-(trimethylsilyl)pyridin-2(*1H*)-one (7i)

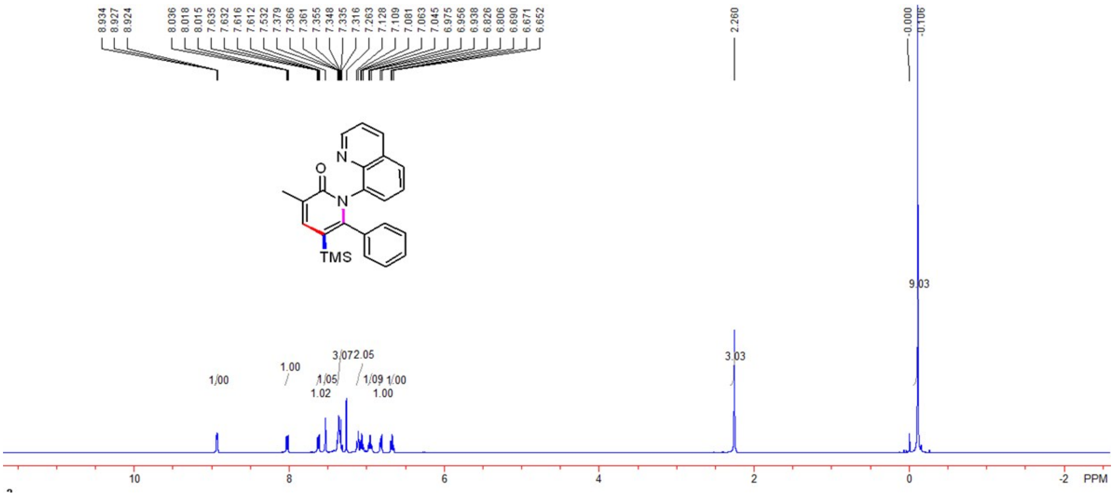

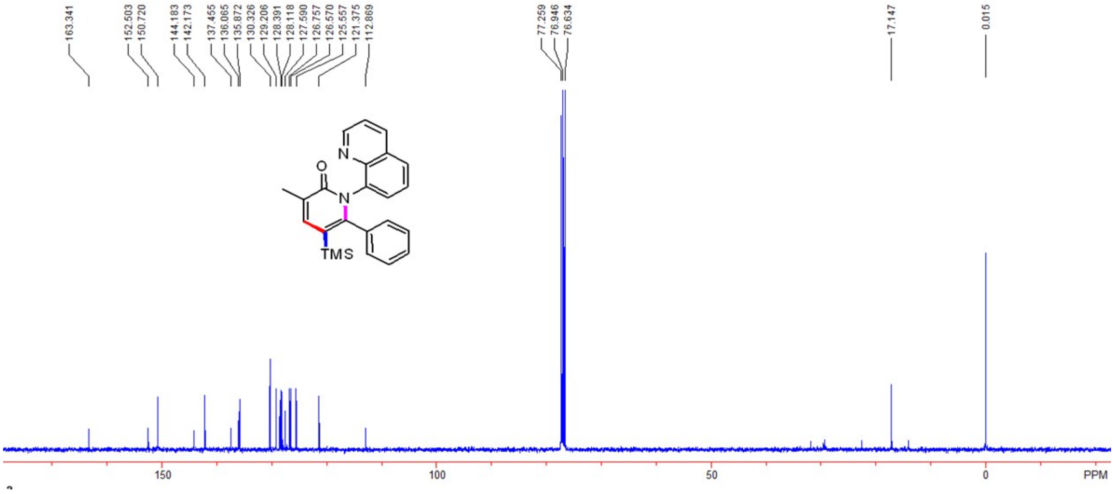

3-methyl-4,6-diphenyl-1-(quinolin-8-yl)-5-(trimethylsilyl)pyridin-2(*1H*)-one (7j)

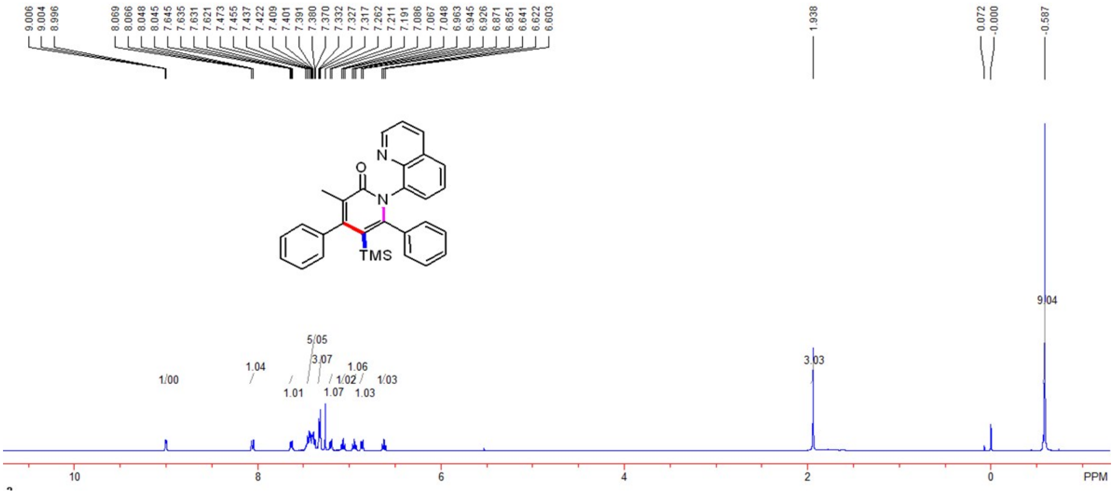

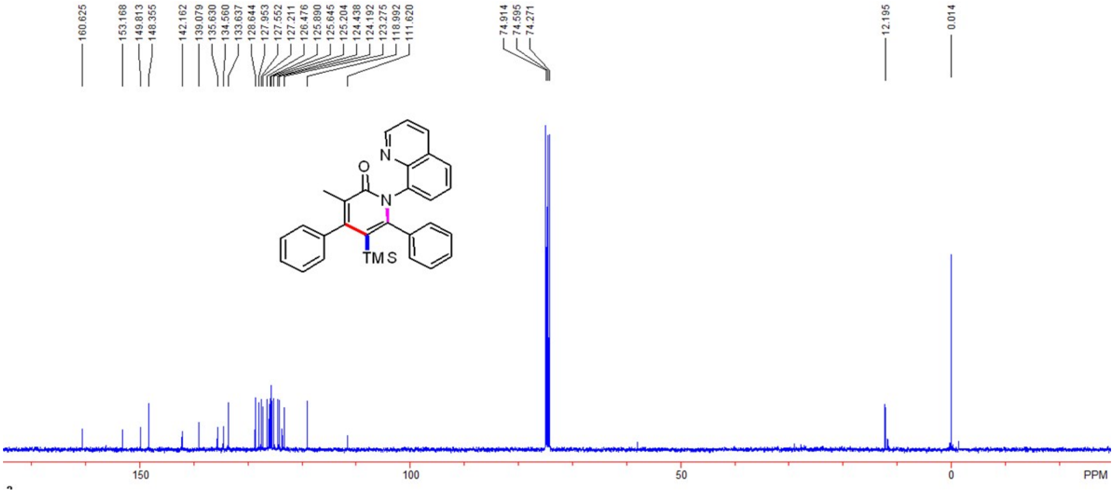

Supplement: RA-009-C9RA06963A-s001 [file RA-009-C9RA06963A-s001.pdf]
